# A comprehensive custom panel design for routine hereditary cancer testing: preserving control, improving diagnostics and revealing a complex variation landscape

**DOI:** 10.1038/srep39348

**Published:** 2017-01-04

**Authors:** Elisabeth Castellanos, Bernat Gel, Inma Rosas, Eva Tornero, Sheila Santín, Raquel Pluvinet, Juan Velasco, Lauro Sumoy, Jesús del Valle, Manuel Perucho, Ignacio Blanco, Matilde Navarro, Joan Brunet, Marta Pineda, Lidia Feliubadaló, Gabi Capellá, Conxi Lázaro, Eduard Serra

**Affiliations:** 1 Hereditary Cancer Group, Program on Predictive and Personalized Medicine of Cancer (PMPPC), Germans Trias i Pujol Research Institute (IGTP), Can Ruti Campus, Badalona, Barcelona, Spain; 2 Hereditary Cancer Program, Joint Program on Hereditary Cancer, Catalan Institute of Oncology, IDIBELL campus in Hospitalet de Llobregat, Catalonia, Spain; 3 Genomics and Bioinformatics Unit, Program on Predictive and Personalized Medicine of Cancer (PMPPC), Germans Trias i Pujol Research Institute (IGTP), Can Ruti Campus, Badalona, Barcelona, Spain; 4 Clinical Genetics and Genetic Counseling Program, Germans Trias i Pujol Hospital, Can Ruti Campus, Badalona, Barcelona, Spain; 5 Hereditary Cancer Program, Joint Program on Hereditary Cancer, Catalan Institute of Oncology, IdibGi in Girona, Catalonia, Spain

## Abstract

We wanted to implement an NGS strategy to globally analyze hereditary cancer with diagnostic quality while retaining the same degree of understanding and control we had in pre-NGS strategies. To do this, we developed the I2HCP panel, a custom bait library covering 122 hereditary cancer genes. We improved bait design, tested different NGS platforms and created a clinically driven custom data analysis pipeline. The I2HCP panel was developed using a training set of hereditary colorectal cancer, hereditary breast and ovarian cancer and neurofibromatosis patients and reached an accuracy, analytical sensitivity and specificity greater than 99%, which was maintained in a validation set. I2HCP changed our diagnostic approach, involving clinicians and a genetic diagnostics team from panel design to reporting. The new strategy improved diagnostic sensitivity, solved uncertain clinical diagnoses and identified mutations in new genes. We assessed the genetic variation in the complete set of hereditary cancer genes, revealing a complex variation landscape that coexists with the disease-causing mutation. We developed, validated and implemented a custom NGS-based strategy for hereditary cancer diagnostics that improved our previous workflows. Additionally, the existence of a rich genetic variation in hereditary cancer genes favors the use of this panel to investigate their role in cancer risk.

Genetic diagnostic laboratories testing for cancer predisposition have rapidly integrated next-generation sequencing (NGS) technologies into their diagnostic workflows^1^,^2^,^3^,^4^. More genes have gradually been included to solve problems like genetic heterogeneity or overlapping clinical manifestations among distinct cancer predisposition syndromes^5^,^6^,^7^,^8^,^9^,^10^. Today, gene panels represent a good compromise between testing just a few genes and obtaining information from the whole exome for routine hereditary cancer testing^11^,^12^,^13^. Comprehensive guidelines, recommendations and standards have been published to guide the development, validation and implementation of NGS-based clinical genetic testing^15^,^16^,^17^,^18^,^19^,^20^,^21^,^22^. This material has aided the adaptation of NGS to the standardized frameworks developed for clinical genetic tests^23^. Despite these advances, for a genetic diagnostic laboratory mastering pre-NGS techniques, the biggest challenge when adopting NGS is to maintain the same degree of understanding and control over the whole process of detecting and interpreting genetic changes^22^,^24^.

The diagnostic activity of the ICO-IMPPC Joint Program for Hereditary Cancer focuses on the detection and interpretation of all inherited genetic variants that confer a higher risk of developing cancer. This activity encompasses all types of hereditary cancer (HC) syndromes, although we work mainly with hereditary colorectal cancer (Familial Adenomatous Polyposis, FAP, and Hereditary Non-Polyposis Colorectal Cancer, HNPCC), hereditary breast and ovarian cancer (HBOC) and neurofibromatoses Type 1 and Type 2 (NF1, NF2) and related disorders such as RASopathies and Phakomatoses. Due to genetic heterogeneity, clinical heterogeneity and overlapping clinical manifestations, diagnostic activity in the field of hereditary cancer requires multiple gene testing^12^,^25^,^26^.

To cover diagnostic necessities, homogenize diagnostic procedures for different conditions and preserve understanding and control over the complete workflow, we developed a comprehensive custom NGS-based diagnostic strategy to be implemented in the routine diagnostics scenario applicable to most of the genes involved in hereditary cancer and related disorders, similarly to other initiatives (Mainstreaming Cancer Genetics)^27^. Our aim was to develop a tool and a diagnostic strategy that would ensure the desired sequencing quality, provide great flexibility in the bioinformatic analysis to enhance clinical utility, and enable us to gather global data on the genetic variation of hereditary cancer.

## Results

### Development and validation of a hereditary cancer NGS panel

#### The I2HCP diagnostics NGS panel design and set up

**Figure 1:**
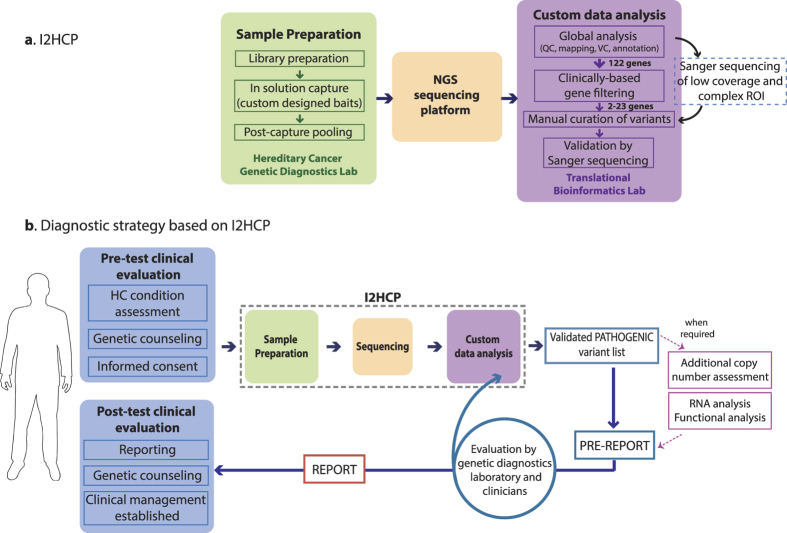
New genetic diagnostic workflow for hereditary cancer. (**a**) Detailed view of the ICO-IMPPC Hereditary Cancer Panel (I2HCP) including the three main steps: sample preparation, sequencing and data analysis. (**b**) The panel is part of the I2HCP diagnostic strategy, which also includes pre- and post-test clinical evaluation, optional additional analysis and evaluation of the pre-report by a multidisciplinary team.

We have developed the ICO-IMPPC Hereditary Cancer Panel (I2HCP), which comprises 122 genes associated with cancer predisposition syndromes and RASopathies (Supplementary Table S1) and a custom data analysis pipeline. All genes had been previously identified as germline-mutated in relation to hereditary cancer (Cancer Census, OMIM or Orphanet). Combining all genes of interest in a single panel simplifies and unifies laboratory procedures in a single workflow when testing for the different conditions. The sequencing results are then filtered during the bioinformatic analysis and only selected genes are analyzed on the basis of the clinical indications (Fig. 1a).

**Figure 2:**
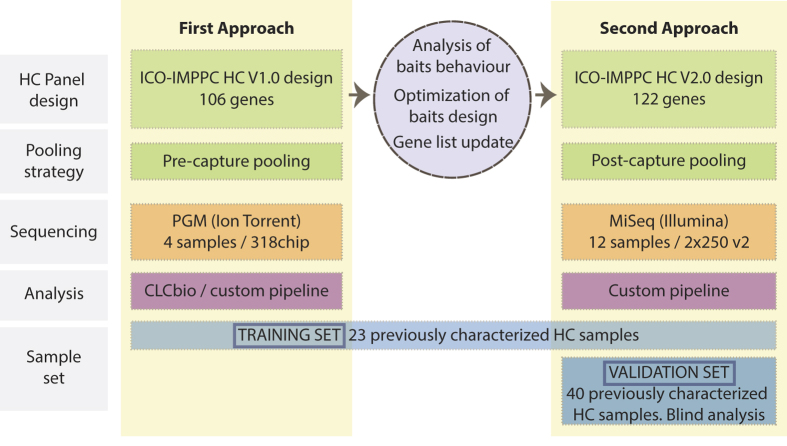
I2HCP set up and validation scheme. Summary of the different components of the two approaches developed. Training and validation sets are specified. The middle circle indicates improvements and changes made when designing the second approach.

To comply with the exhaustive analysis needed for diagnostics, the I2HCP regions of interest (ROIs) spanned all protein coding regions and intron-exon boundaries (−35/+20 bp), including all regions usually left untargeted for technical reasons such as repeats and homologous regions. Sufficient coverage was sought to ensure that all bases within ROIs were covered at a minimum of 30x. In a first approach, a training set (n = 23, see Materials and Methods and Supplementary Table S2) was used to evaluate the performance of a first version of the SureSelect bait library, consisting of 106 genes that were sequenced in an Ion Torrent PGM sequencer using a 318 chip, pooling 4 samples before enrichment. Data were analyzed using both CLC Genomics Workbench (Qiagen) and our custom NGS pipeline (Fig. 2). This approach showed a good capture yield and a low percentage of non-covered regions: 1.54 × 10^6^ ± 0.5 × 10^6^ SD reads per sample were produced, the mean depth of coverage was 490.7 ± 221.5 SD, and more than 86% ± 4% SD of the targeted ROI bases was covered ≥ 30x. 123 out of 136 pathogenic and non-pathogenic variants previously identified in the training set were correctly detected, providing a sensitivity of 90.4% (84.2–94.8%). However, this first approach failed to identify 7 out of 23 pathogenic variants, mostly due to their location within homopolymers (Supplementary Table S2).

On the basis of these initial results, two changes were made: a) To improve the percentage of well-covered regions, we studied coverage distribution and performed a systematic bait library redesign to increase the coverage of poorly covered regions (Supplementary Fig S1). In addition, we increased to 122 the number of genes in the panel (Supplementary Table S1); b) To improve variant calling quality, we analyzed all data using only our custom NGS pipeline, to ensure greater control over variant calling algorithms and parameters. We also decided to test the performance of a second sequencing platform: Illumina MiSeq.

Using the same training set of samples and the new bait library design, we set up a second approach based on the SureSelect XT protocol for Illumina, pooling 12 samples after enrichment and sequencing them in a Miseq 2 × 250 v2 cartridge (see Materials and Methods). Sequencing data were analyzed using our custom NGS pipeline (Fig. 2). 3.3 × 10^6^ ± 0.83 × 10^6^ SD paired reads were obtained per sample, the mean depth of coverage was 495 ± 170 SD and the coverage uniformity was of 0.31 ± 0.009 SD. More than 98.9% ± 0.7% SD of the targeted bases was covered ≥ 30× (Supplementary Fig S2). Detailed sequencing statistics are given in Supplementary Table S3. This new approach correctly identified 22 of the 23 pathogenic variants in the training set. The missing pathogenic mutation was located in the first exon of *MSH6* gene and was not detected due to low coverage. As established in the I2HCP strategy (see implementation section), this exon was Sanger sequenced and the pathogenic variant identified (Supplementary Tables S2 and S4). In addition, all 113 previously identified non-pathogenic SNVs and indels were correctly detected (Supplementary Table S4). Taking into account all pathogenic and non-pathogenic variants previously identified in the training set, NGS data provided a sensitivity of 100% (97.3–100%). We also detected 78 additional SNV in the ROIs of the genes previously tested by different methodologies (CSCE, cDNA sequencing, etc.). Most of these variants were known SNPs and all of them were either homozygous or located in intronic regions and thus not detected by pre-NGS analysis (Supplementary Table S4). Overall, no false positive was identified, resulting in a specificity of 100% (98.3–100%) for the training set.

#### Data analysis pipeline for NGS-based genetic diagnostics

NGS data were processed using a custom diagnostics-oriented data analysis pipeline, based on standard tools (BWA, VarScan2 and Annovar) and an extensive set of custom R scripts (see Material and Methods and Supplementary Figure S4). By creating a custom pipeline we were able to include several specific quality controls and measures such as targeted bases not reaching diagnostic quality, global and exon coverage measures, specific measures of variant quality for difficult regions, etc. We also ensured full control over the software used: the algorithms, the software versions and their specific parameters, which are locked for each version of the pipeline, and the data sources used for the basic analysis (genome version, gene models…) and annotations (dbSNP, ClinVar…). All data were stored in a relational database and a custom pipeline management system was used to keep track of the analyses carried out. Finally, the full pipeline is under version control on our institution’s Git servers, enabling us to keep track of every modification and to revert to a previous version if necessary. In addition, in contrast to research-oriented NGS data analysis pipelines, our pipeline focuses on reducing false negatives at the expense of potentially increasing false positives, the latter of which would be uncovered in the Sanger variant validation step of the I2HCP strategy. Finally, we developed the first version of an exon-level copy-number calling algorithm based on successive steps of coverage depth normalization (see Material and methods). Although this is a preliminary version and not yet validated for diagnostics, it allowed us to correctly identify the true copy number changes in the training set (Supplementary Table S2) and therefore to increase the mutation detection yield.

#### Validation

**Figure 3:**
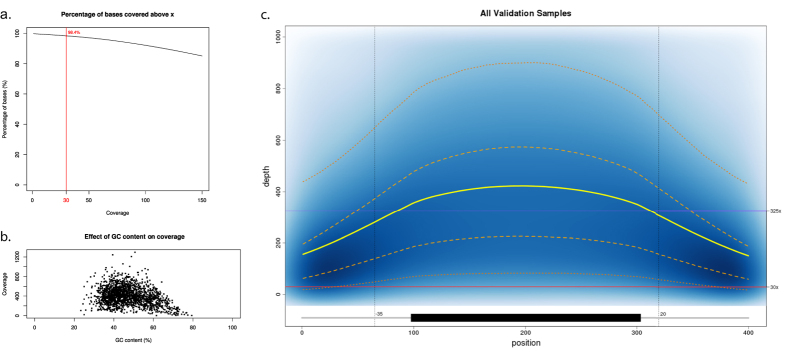
Coverage Analysis. Analysis of the depth of coverage of the samples in the validation set. (**a**) The percentage of bases of the ROIs above a given coverage level. The red vertical line corresponds to C30 (98.4%). (**b**) The relationship between GC content and depth of coverage. Each dot represents a single exon. GC content was computed as the percentage of G/C in the exon sequence. The coverage was computed as the mean coverage of the exon over all samples in the validation set. (**c**) Density plot showing the coverage distribution along all ROIs over all samples in the validation set. The exon is represented as a black box and intron/exon boundaries as grey lines. The exonic coverage was computed as the mean over 200 equally sized windows to normalize the exon length. Intronic coverage is plotted as one position per base. The blue background represents the density of exons with a given coverage at a given position. The yellow line represents the mean coverage, the dashed orange lines represent the Q1 and Q3 quartiles and the dotted orange lines the 5 and 95 percentiles. Finally, the purple horizontal line represents the mean coverage and the red line the minimum coverage threshold of 30x.

After the I2HCP panel had been set up using the training set, an independent group of 40 samples was used as a validation set (Material and Methods; Fig. 2; Supplementary Table S5). These samples had previously been genetically characterized, with 36 found to have a known disease-causing mutation and 4 no pathogenic variant. We focused the variant analysis only on those genes tested in the previous genetic diagnostic workflows (Supplementary Table S1). The whole validation process was performed blindly. Each sample produced 3.1 × 10^6^ ± 0.58 × 10^6^ SD paired reads, the mean depth of coverage was 398 ± 138 SD, the coverage uniformity was of 0.31 ± 0.02 SD, and 98.4% ± 1.0% SD of the targeted bases was covered ≥ 30× (Fig. 3; Supplementary Figure S2; Supplementary Table S3), similar to the performance achieved in the training set. This new approach correctly identified all 36 pathogenic variants in the validation set, including substitutions, small insertions/deletions (up to 19 bp), and deletions of a single exon and a whole gene. No pathogenic variants were identified in the 4 samples that previously tested negative (see Supplementary Table S5). In addition, 180 out of 186 previously identified variants were detected (VUS and polymorphisms). Of the 6 missing variants, one was located in a low-coverage region of the *MSH6* gene, and the remaining 5 in a highly complex region of *PMS2*. These regions are routinely re-analyzed by Sanger sequencing (see Implementation section; Supplementary Figure S3). Therefore, considering the 358 known variants in the training and validation sets, the analytical sensitivity of the panel was 98.4% (96.4–99.4%) and the analytical specificity was of 100% (99–100%). In addition, considering the whole set of 122 genes, within-run precision (repeatability) was 98% (97–98.7%) and between-run precision (reproducibility) was 95.7% (94.6–96.6%), both within the optimal range for diagnostic purposes (ref. 19; see Material and Methods for details).

### Implementation into routine genetic testing: an I2HCP-based diagnostic strategy

Once the I2HCP had been validated, we modified the overall HC diagnostic strategy to unlock the potential of the new panel and integrated it into routine genetic testing (Fig. 1b). As in pre-NGS diagnostics, the clinical presentation of patients drives the diagnostic approach, thus a pre-test clinical evaluation of the hereditary cancer condition in question is required before initiating genetic testing. Next, sample preparation, NGS sequencing and data analysis are performed for the whole set of 122 genes; only genes with current clinical utility for each hereditary cancer condition are then further analyzed up to variant interpretation. The groups of genes with clinical utility for HBOC, FAP, HNPCC and neurofibromatosis have been pre-defined by clinicians and the genetic diagnostics team (Supplementary Table S1). The panel format provides the flexibility to select *ad hoc* groups of genes for other conditions or particular clinical presentations or to broaden the test, even after a first round of analysis. Any ROI of the analyzed gene set with a single base below 30x coverage is Sanger sequenced to ensure diagnostic quality. The total number of Sanger sequencing required is low and the regions involved are recurrent (Supplementary Table S6 and Figure S3). We also use Sanger sequencing as an independent technique to validate all reportable variants.

Since the I2HCP diagnostic strategy was implemented into our laboratory routine, more than 150 samples have been processed. In general, quality parameters have improved with respect to the validation set (3.2 × 10^6^ ± 1.5 × 10^6^ SD paired reads; mean depth 452 ± 225 SD; uniformity 0.31 ± 0.02 SD; C30 99.2 ± 0.7 SD). At the same time, the custom development of I2HCP has provided the plasticity required for continuous development and rapid updates. Since its implementation in routine diagnostics, the sequencing kit has changed from v2 (2 × 250 cycles) to v3 (2 × 300 cycles), the number of genes has increased to 126 (including the genes *LZTR1, PIK3CA, RASA2* and *GRB2*), the number of samples per MiSeq run has increased from 12 to 16, and multiple upgrades in variant annotation have been made.

Despite the excellent analytical sensitivity and specificity it provides, the I2HCP NGS panel does not consistently detect some types of pathogenic variants present in the mutational spectrum of these genes and syndromes (e.g., deep intronic mutations, insertion of repetitive elements, etc.). Thus, other techniques such as mRNA analysis could be integrated to complement the I2HCP in particular cases. In addition, for some conditions, constitutional copy-number alterations (CNA) account for a considerable percentage of disease-causing mutations. A clear example is neurofibromatosis type 1, in which about 7% of mutations in the *NF1* gene are due to total gene deletions or intragenic CNAs^28^. Analysis of the CNAs of specific genes is part of the I2HCP diagnostic strategy and is mainly carried out by MLPA, depending on the condition and clinical status of each patient. Although the I2HCP is not yet validated for routine genetic diagnostics, it can be used for CNA analysis. Since the I2HCP strategy was introduced, 100 samples have been complemented by MLPA analysis of different genes (171 MLPA tests). We have also analyzed the presence of CNAs in these 100 cases by applying an exon-level copy-number calling algorithm as part of the I2HCP pipeline. Ninety-nine samples have been analyzed (1 sample, corresponding to 3 MLPA analyses, did not reach the minimum quality criteria for analysis). Of the remaining 168 CNAs analyses, 159 were negative and 9 positive (8 whole gene deletions and one two-exon deletion). In all cases, MLPA and I2HCP results were concordant (Supplementary Table S7).

The overall analysis, including genetic variant interpretation and complementary tests, is summarized in a pre-report that is evaluated by a multidisciplinary team. Depending on the results and the patient’s clinical presentation, clinicians may request the analysis of additional genes in the I2HCP panel. Since NGS data are already available and processed, the analysis can start at the gene-filtering step of the pipeline. Finally, a report compliant with EuroGentest recommendations is generated and clinicians perform a post-test clinical evaluation concerning genetic counseling and clinical management.

#### The new genetic testing strategy improves HC diagnostics

One of the questions raised by the enhanced testing potential of the I2HCP-based strategy is whether retrospective re-analysis is warranted for particular cases that tested negative in previous diagnostic workflows or for uncertain clinical presentations that did not fully meet testing criteria. As a pilot study, we selected a group of 14 cases for re-analysis with the new strategy (Material and methods; Table 1). This group comprised 2 HBOC, 10 HNPCC positive for the Amsterdam criteria, 1 Schwannomatosis and 1 patient with clinical suspicion of NF1. In all cases, in addition to the respective clinically-driven gene lists (Supplementary Figure S1), we analyzed all 122 genes in the I2HCP. As seen in Table 1, the re-analysis enabled us to solve one case with an uncertain clinical diagnosis: a whole *CDKN2A* deletion causing melanoma and neural system tumor syndrome (OMIM: 155755) was identified in the patient with clinical suspicion of NF1 (Sample R2) (Supplementary Figure S5). The re-analysis also allowed us to increase the sensitivity of mutation detection by analyzing the pre-defined sets of genes (e.g.: Samples R1 and R4). Finally, the analysis of the whole I2HCP identified variants in new genes with potential impact on the clinical phenotype (e.g., samples R3, R5 and R6).

### Variation landscape of hereditary cancer genes in hereditary cancer patients

**Figure 4:**
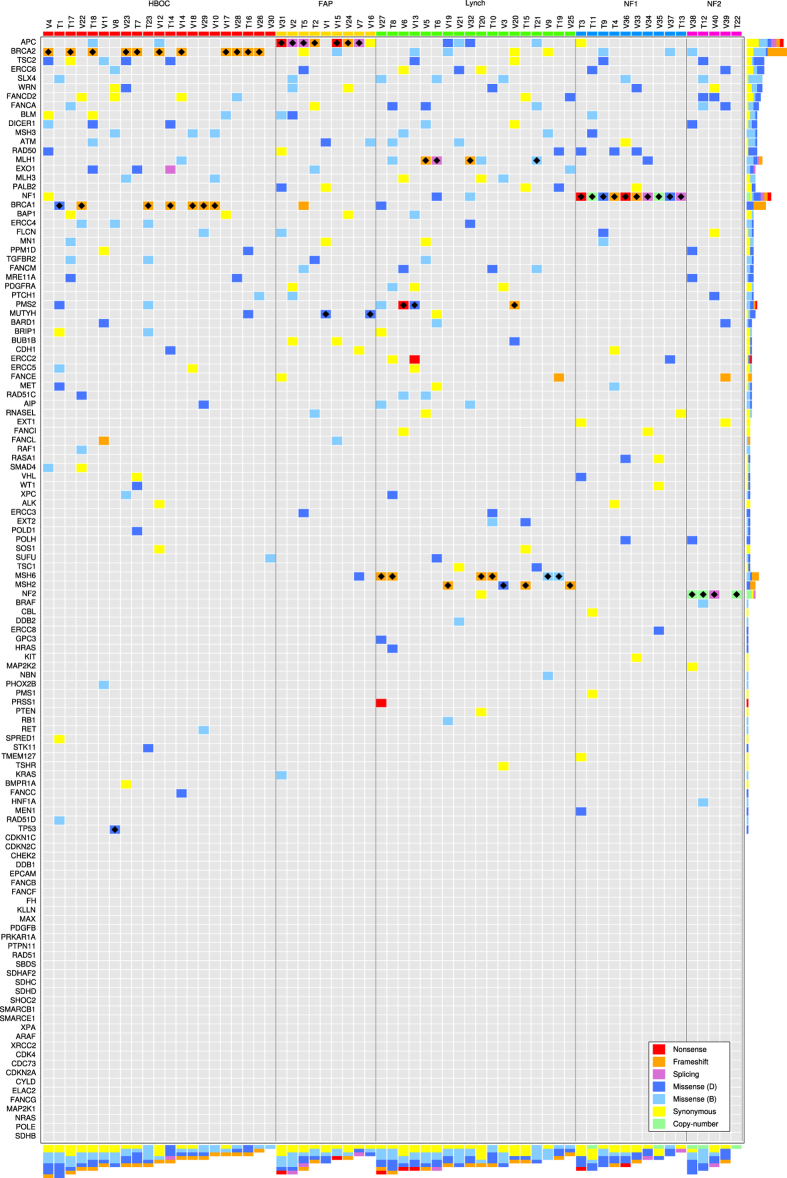
Variation landscape of hereditary cancer genes. Matrix representation of the rare variants found in the training and validation samples. Only variants with a MAF below 1% in all populations from 1000 Genomes or ExAc projects are included. Each row represents a gene and each column a sample. Samples are ordered by clinical condition and by total number of variants. Genes are ordered by total number of variants without taking into account the disease-causing mutation. Each cell of the matrix is colored according to the type of variant found: red for nonsense, orange for frameshift, purple for splicing, blue for missense, yellow for synonymous and green for copy-number variants. If more than one variant was found in a given gene on a given sample, the color of the most damaging variant was used. Missense variants were further separated into two groups according to *in-silico* functional impact predictions using data from Polyphen2 HDIV and HVAR, SIFT, PROVEAN and MutationTaster. If more than 3 of the algorithms classified the variant as neutral, it was considered a neutral variant and colored in light blue; otherwise it was considered a possibly damaging variant and colored with dark blue. Bars on the right hand side represent the total number of variants per gene and their type. The bottom bars represent the total number of variants per sample. Finally, black diamonds denote the disease-causing mutations.

I2HCP contains the majority of genes currently associated with hereditary cancer. The creation of a comprehensive panel was intended primarily to facilitate diagnostic activity, but it was also designed to facilitate the compilation of all genetic variants present in genes associated with cancer predisposition. Thus, it was possible to generate a global view of the constitutional variation present in these genes from the raw data produced for genetic testing. This opens the possibility of studying not only the disease-causing mutation but also the contribution of this variation to the presentation and evolution of the disease. Figure 4 shows the variation landscape of the genes in I2HCP for the 63 individuals in the training and validation sets. In particular, it contains the variants present in coding regions and canonical splice sites with a minor allele frequency (MAF) below 1%.

The global analysis revealed a complex variation landscape that coexists with the disease-causing mutation, including nonsense, frameshift, splicing, missense and synonymous variants. The number of variants per individual ranges from 1 to 9, with an average of 5, and presents no differences among HC conditions. Similarly, not taking into account the disease-causing mutation, there is diversity in the number of variants per gene, with the top 10% of genes accounting for around 40% of the genetic variation. In 27 cases a patient had more than one variant in the same gene, accounting for 55 out of 278 variants present. In 8 of the 27 cases these multiple variants accumulated in the disease-causing gene (data not shown).

Although this variation landscape represents only a small number of HC patients, some notable observations can be made. For instance, although tuberous sclerosis patients were not considered in this study, the *TSC2* gene was among the genes that accumulated more variation, most of which had a very low MAF. Additionally, most nonsense, frameshift and splicing variants concentrate in DNA repair genes. Finally, there are a number of potentially pathogenic variants in genes not directly related to the phenotype that could be worth exploring. It would therefore be interesting to study the complexity of this variation landscape and its relationship with the presented cancer phenotypes in a larger sample.

## Discussion

The adoption of NGS technology by a routine genetic diagnostics laboratory presents various challenges^29^, among them the new competences required for both wet and dry labs and the complete reorganization of diagnostic activity. However, crucial to genetic testing is the ability to maintain understanding and control over the whole diagnostic workflow, to identify the potential limitations in the detection and interpretation of genetic variants and the implications for the diagnostic report. The prospective integration of new NGS methodologies and more complex bioinformatic analysis raised concerns about possible loss of overall control, so we decided to customize our NGS diagnostic strategy as far as possible. This was achieved by developing an NGS-based workflow comprising a panel of 122 HC genes and a clinically driven custom data analysis pipeline. We designed ROIs to comprehensively sequence all coding exons and desired intron-exon boundaries of hereditary cancer genes and RASopathies according to our diagnostic activity. The custom nature of I2HCP provides the plasticity for continuous and rapid updating. In the few months since the I2HCP strategy was implemented for routine diagnostics, the number of genes in the panel has increased, sequencing procedures and pipeline analysis have been updated and more samples per run can be tested.

According to Eurogentest NGS recommendations^18^ I2HCP can be considered a Type A test, given the high sensitivity and specificity achieved during the development and validation processes. However, it must be taken into account that the panel was set up by analyzing HBOC, FAP, HNPCC and neurofibromatosis patients. Although the quality parameters apply to all genes tested, for genes and conditions with particular mutational spectrums or testing complexities a specific analysis of I2HCP performance could be required.

The custom panel provides great flexibility, allowing for the analysis of pre-established gene sets for particular clinical conditions but also the testing of specific genes on demand (up to the entire set of 122 genes) in those cases with distinct clinical presentations. The customization of the panel and its comprehensive nature foster interplay and dialog between clinicians and the genetic diagnostics team for panel design, evaluation of results, the possible need to analyze additional genes, and reporting. In addition, the results of the re-analysis pilot study show the potential of applying the new strategy to previous negative tests and to patients who do not fully meet clinical criteria.

The I2HCP diagnostic strategy compensates for the limitations of the panel by integrating Sanger sequencing of complex and low-coverage regions, mRNA analysis when required and CNA analysis by MLPA. The I2HCP CNA detection algorithm has shown a 100% concordance with MLPA results in the 168 assessable tests performed so far. Robust development of this algorithm could substantially reduce the number of MLPAs required for diagnostics.

The global analysis of all genes in the panel revealed a complex variation landscape that coexists with the known disease-causing mutation. The number of variants identified per individual and the frequency of variants per gene were consistent with previous reports using other panels^11^. The study of this global variation and the systematic collection of genetic and phenotypic data in a higher number of patients could provide the evidence needed to establish additional genes as conferring cancer predisposition and to make reliable risk estimates for patient management and counseling^30^,^31^,^32^.

## Conclusions

In summary, we developed and validated a custom NGS-based diagnostic strategy for hereditary cancer and implemented it into our routine diagnostic activity. The mutation detection rate increased, while maintaining control over the whole process. Complete analysis of the 122 hereditary cancer genes tested revealed a complex variation landscape that coexists with the disease-causing mutation. Analysis of this landscape in a higher number of patients could help to better estimate individual cancer risk.

## Methods

### Subjects

This study was approved by the IMPPC scientific direction and carried out in accordance with IMPPC guidelines. Signed informed consent was obtained from all participants. Genomic DNA from 73 unrelated individuals clinically diagnosed with a cancer predisposition syndrome was obtained from blood lymphocytes using standard protocols. The 73 samples were grouped in three different sample sets: training, validation and re-analysis. The training set comprised 23 samples from patients who met the clinical criteria for HBOC (n = 7), FAP (n = 2), HNPCC (n = 7), NF1 (n = 6) and NF2 (n = 1). The validation set comprised 13 HBOC patients, 7 FAP, 11 HNPCC, 5 NF1, 2 NF2 and 1 patient who met the clinical criteria for Schwannomatosis, for a total of 40 samples. These samples contained 222 variants (36 pathogenic plus 186 VUS and polymorphisms), which was sufficient to determine specificity and sensitivity with a 95% CI of width <1.3%^23^. All samples had been genetically tested using pre-NGS workflows and methods (cDNA and DNA Sanger sequencing, conformation-sensitive capillary electrophoresis (CSCE), denaturing high-performance liquid chromatography (DHPLC) and multiplex ligation-dependent probe amplification (MLPA)). These samples presented a broad mutational spectrum, including single-nucleotide variants (SNVs), small insertions and deletions and multiple exon deletions, many of them located in complex sequence contexts (Supplementary Figure S6). Finally, the re-analysis set consisted of 14 individuals: 9 HNPCC positive for the Amsterdam criteria, 1 NF1 and 4 patients from the validation set with no pathogenic mutation as detected by pre-NGS testing workflows (Supplementary Table S1). Moreover, the global results for the 150 patients genetically diagnosed using I2HCP were compared to the training and validation sets. The testing criteria for these patients were based on current international clinical criteria guidelines^33^. Samples were codified and mutation analysis was performed blindly for the validation set.

### Enrichment

We used Agilent eArray to design our SureSelect bait library V1 (Agilent, California, USA), covering 106 genes for a total of 0.45 Mb. For each gene, we defined the ROIs as all coding exons and intron/exon boundaries (−35/+20 bp) of all translated isoforms according to NCBI human genome build 37 (GRCh37) and the Ensembl release 67. All ROIs were exhaustively covered with capture baits. A second design was developed after studying the behavior of V1 baits to improve capture results. In short, we monitored the coverage of all exons, identified problematic regions, performed a systematic rebalancing of baits and increased bait tiling using custom algorithms to improve capture of poorly covered areas and to enhance coverage uniformity (Supplementary Figure S1). The bait library V2 included 122 genes responsible for most of the cancer predisposition syndromes and RASopathies (Supplementary Table S1), for a total of 0.5 Mb of targeted DNA.

### Sample preparation and sequencing

DNA was sonicated using a Covaris S2 (Covaris, Woburn, MA, USA). Sample preparation was performed following the SureSelect XT protocol for Ion Torrent or MiSeq. In the first approach, samples were enriched with bait library V1 after combining 4 equimolar indexed samples (pre-capture pooling) and sequenced in a PGM (Ion Torrent) 318 chip with One Touch 200 DLv2 template reagents and Ion PGM 200 sequencing reagents. In the second approach, 12 samples were enriched with bait library V2 according to the manufacturer’s instructions (Agilent) with minor modifications^34^, pooled after capture and sequenced in a MiSeq (Illumina) with Reagent Kit v2, 2 × 250.

### Validation by Sanger Sequencing

For the subset of genes analyzed for a patient, any ROI with at least one base below 30x was Sanger sequenced using standard protocols (primer sequences available upon request). 30x minimum coverage was established as per De Leener *et al*.^35^. Reportable variants (all pathogenic variants and VUS only for some syndromes) were also validated by Sanger sequencing. Human Genome Variation Society (www.hgvs.org) nomenclature guidelines were used to name the mutation at the DNA level and the predicted resulting protein.

### Bioinformatic Analysis

NGS data were processed using a custom data analysis pipeline based on standard tools. In short, fastq files were mapped against the GRCh37 human genome assembly corresponding to Ensembl release 67^36^ using BWA mem^37^ and a sorted bam file was created with samtools^38^. Exhaustive coverage metrics were produced using a combination of bedtools^39^ and custom R and Bioconductor^40^ scripts. Variants, including substitutions and small insertions and deletions, were called using VarScan2^41^ with the following parameters: –min-coverage 10 –min-reads2 2 –min-avg-qual 15 –min-var-freq 0.1 –strand-filter 0. Finally, variants were annotated with a combination of annovar^42^ and custom scripts. bam-readcount was then used to compute additional quality parameters (quality of the bases supporting the reference and alternative alleles, position of the changes in the reads, number of mismatches present in reads supporting the alternative allele, etc.) that were combined to produce a set of variant quality indicators to guide the variant validation process. Final variant annotation included: basic variant quality parameters, gene and transcript annotations and effects, presence in variation databases (dbSNP and ClinVar), population frequencies (1000 G, ExAC and ESP6500) and in-silico prediction of effects in protein function (Polyphen2, SIFT, MutationAssessor, MutationTaster, PROVEAN, CADD). All information, including coverage metrics, variants, variant quality estimators and annotations, was stored in a PostgreSQL database. Variants were filtered using the regioneR bioconductor package^43^ according to the clinical indication prior to generating the variant lists. The preliminary algorithm for exon-level copy-number estimation includes different normalization steps on the exon mean depth of coverage (including mean sample coverage and mean exon coverage across samples), similar to Kang *et al*.^44^, and was run using a set of custom scripts. CLC Genomics Workbench v6 (Qiagen) was used to analyze data from I2HCP V1. Repeatability was measured by comparing the variants called for four samples prepared and sequenced twice under the same conditions. Reproducibility was calculated for twelve samples prepared once and sequenced twice in independent runs. In both cases, the concordance was computed as the number of common variants divided by the total number of variants identified. 95% confidence intervals were computed using the exact method from Hmisc R package. Uniformity was defined as the percentage of bases with a coverage within ± 20% of the mean coverage. C30 was defined as the percentage of ROI bases with coverage ≥30x.

## Additional Information

**How to cite this article**: Castellanos, E. *et al*. A comprehensive custom panel design for routine hereditary cancer testing: preserving control, improving diagnostics and revealing a complex variation landscape. *Sci. Rep.* **7**, 39348; doi: 10.1038/srep39348 (2017).

**Publisher's note:** Springer Nature remains neutral with regard to jurisdictional claims in published maps and institutional affiliations.

## Supplementary Material

Supplementary Information

## Figures and Tables

**Table 1:** Sample re-analysis pilot study.

Sample	Cancer history (proband and 1st and 2nd degree relatives)	Clinical criteria	Previous analysis	I2HCP analysis	Coding non-synonymous infrequent detected variants	Functional prediction
Sample_R1	**Proband: endometrial c (59y).** Mother: breast c (41y), colon c (42y). Maternal aunt: endometrial c (45y). Maternal uncle: Gastric (57)	HNPCC, Amsterdam Criteria	Conserved MLH1/MSH2 and non-evaluable MSH6 expression in proband’s tumor. *MSH6* genetic analysis negative	1) Lynch genes set	**MSH2:NM_000251:exon3:c.G437T:p.G146V**	Neutral
					**MSH6:NM_000179:exon9:c.3952_3960del:p.1318_1320del**	Deleterious
				2) Whole I2HCP	EPCAM:NM_002354:exon3:c.G267C:p.Q89H	Deleterious
					APC:NM_001127510:exon11:c.G1139A:p.R380Q	Deleterious
					PMS2:NM_000535:exon11:c.C1169T:p.A390V	Neutral
					FANCC:NM_000136:exon2:c.C77T:p.S26F	Deleterious
					SDHD:NM_003002:exon1:c.G34A:p.G12S	Neutral (ClinVar:PAT)
					FANCM:NM_020937:exon20:c.C4799T:p.T1600I	Deleterious
					BRCA1:NM_007294:exon10:c.G3119A:p.S1040N	Neutral
					BBRCA1:NM_007294:exon10:c.C2521T:p.R841W	Deleterious
					MN1:NM_002430:exon1:c.G56A:p.G19D	Deleterious
Sample_R2	**Proband: Malignant fibrous histiocytoma (an undifferentiated pleomorphic sarcoma), a glioblastoma multiforme and 3 neurofibromas**	NF1	None	1) NF1 genes set	No candidate variant detected	
				2) Whole I2HCP	**Whole CDKN2A gene deletion. Validated by MLPA**	Deleterious OMIM: 155755
					**ATM:NM_000051:exon50:c.C7375G:p.R2459G**	Neutral
Sample_R3 (V11)	**Proband: breast c (46y), skin c (49y), uterine c (60y), skin c (60y), thyroidectomy due to multinodular hyperplasia (45y).** Father: gastric c (55y). Sister: ovarian c (Brenner t., 39y). Sister’s son: breast c (34y, BRCA2 VUS c.5729A>T carrier).	HBOC	BRCA1/2 genetic analysis negative	1) HBOC genes set	No candidate variant detected	
				2) Whole I2HCP	FANCL:NM_001114636:exon14:c.1111_1114dupATTA:p.Thr372Asnfs*	Deleterious
					BARD1:NM_000465:exon11:c.C2191T:p.R731C	Deleterious
					CHEK2:NM_007194:exon14:c.C1525T:p.P509S	Neutral
					PMS2:NM_000535:c.1866G>A:p.M622I	Insight: Class 1
Sample_R4 (V30)	**Proband: breast c (46y).** Mother: breast c (77y).Maternal aunt: ovarian c (58y). Maternal uncle: CCR (67y). Paternal aunt-1: ovarian c (56y). Paternal aunt-2: ovarian c ? Paternal uncle: CCR ?	HBOC	BRCA1/2 genetic analysis negative	1) HBOC genes set	**PALB2:NM_024675:exon9:c.G2993A:p.G998E**	Deleterious
					PALB2:NM_024675:exon5:c.G2014C:p.E672Q	Neutral
				2) Whole I2HCP	SUFU:NM_016169:exon7:c.G856A:p.E286K	Neutral
Sample_R5	**Proband: 2 breast c (62, 69y), endometrial c (77y).** Son: prostate c (64y). Mother: breast c (75y). Father: colon c (52y). Sister: colon c (49y). Brother: prostate c (74y).	HNPCC, Amsterdam Criteria	MSH2 and MSH6 LoE in proband’s endometrial tumor. MSH2 & MLH1 genetic analysis negative	1) Lynch genes set	No candidate variant detected	
				2) Whole I2HCP	**PPM1D:NM_003620:exon6:c.1736_1737insA:p.T579fs**	Deleterious
					XPC:NM_004628:exon2:c.C142T:p.L48F	Neutral
					WRN:NM_000553:exon9:c.G1149T:p.L383F	Neutral
					WRN:NM_000553:exon25:c.G2983A:p.A995T	Neutral
					MLH3:NM_014381:exon2:c.G1870C:p.E624Q	Deleterious
					PALB2:NM_024675:exon5:c.C2135T:p.A712V	Neutral
Sample_R6	**Proband: colon c (43y).** Mother: colon c (73y). Maternal uncle-1: colon c (22y). Maternal uncle-2: colon c (41y). Maternal aunt: colon c (73y)	HNPCC, Amsterdam Criteria	Microsatellite stability in proband’s tumor. MSH2 & MLH1 genetic analysis negative	1) 3 Lynch genes set	No candidate variant detected	
				2) Whole I2HCP	**BMPR1A:NM_004329:exon4:c.A223G:p.T75A**	Deleterious
					FH:NM_000143:exon7:c.C926T:p.P309L	Deleterious
					RAD50:NM_005732:exon16:c.C2548T:p.R850C	Deleterious
					ATM:NM_000051:exon37:c.A5558T:p.D1853V	Deleterious
					SLX4:NM_032444:exon15:c.A5501G:p.N1834S	Neutral
Sample_R7 (V21)	**Proband: colon c (39y).** Mother: gastric c (61y). Maternal grandfather: skin c (60y).	HNPCC, Bethesda criteria	Microsatellite instability and MLH1 LoE in proband’s tumor. MLH1 genetic analysis negative	1) Lynch genes set	APC:NM_000038:exon16:c.T3920A:p.I1307K	Neutral
				2) Whole I2HCP	ERCC6:NM_000124:exon10:c.C1996T:p.R666C	Deleterious
					ERCC6:NM_000124:exon18:c.A3122C:p.Q1041P	Neutral
					DDB2:NM_000107:exon9:c.G1228A:p.A410T	Neutral
					ATM:NM_000051:exon41:c.A6084T:p.Q2028H	Neutral
					FANCM:NM_020937:exon21:c.A5627G:p.N1876S	Neutral
					EXO1:NM_003686:exon10:c.G1378C:p.V460L	Neutral
Sample_R8	**Proband: Biliary tract c (43y).** Father: colon c (52y). Brother: colon c (42y). Sister: breast c (55y)	HNPCC, Amsterdam Criteria	MSI-H and MLH1 LoE in proband’s tumor. MLH1 genetic analysis negative	1) Lynch genes set	No candidate variant detected	
				2) Whole I2HCP	ERCC4:NM_005236:c.2624A>G:p.E875G	Deleterious
Sample_R9	**Proband: colon c (59y), lung c (67y).** Father: colon c (68y). Sister: colon c (45y). Sister’s son: leukaemia (25y). Brother: colon c 63(y)	HNPCC, Amsterdam Criteria	MLH1 LoE in proband’s tumor. Absence of MLH1 somatic methylation. MLH1 genetic analysis negative	1) Lynch genes set	PMS2:NM_000535:c.1866G>A:p.M622I	Insight: Class 1
				2) Whole I2HCP	PRSS1:NM_002769:exon5:c.G617C:p.C206S	Deleterious
					PALB2:NM_024675:exon9:c.G2993A:p.G998E	Deleterious
Sample_R10	**Proband: colon c (31y).** Father: 3 colon c (67, 67 and 68y). Paternal aunt: endometrial c (50y)	HNPCC, Amsterdam Criteria	MLH1 LoE in proband’s tumor. Absence of MLH1 somatic methylation. MSH2 & MLH1 genetic analysis negative	1) Lynch genes set	No candidate variant detected	
				2) Whole I2HCP	EXO1:NM_130398:c.1918C>G:p.P640A	Neutral
					PTPN11:NM_002834:exon14:c.C1658T:p.T553M	Neutral
					FANCA:NM_000135:exon27:c.C2574G:p.S858R	Neutral
					FLCN:NM_144997:exon9:c.G979A:p.A327T	Neutral
					MN1:NM_002430:exon1:c.G3550C:p.E1184Q	Deleterious
Sample_R11	**Proband: colon c (58y).** Mother: colon c (70y). Brother: colon c (47y)	HNPCC, Amsterdam Criteria	MSH6 LoE in proband’s tumor. MSH2 & MSH6 genetic analysis negative	1) Lynch genes set	No candidate variant detected	
				2) Whole I2HCP	BARD1:NM_000465:exon7:c.G1670C:p.C557S	Neutral
					MRE11A:NM_005590:exon13:c.C1475A:p.A492D	Deleterious
					SLX4:NM_032444:exon15:c.A5501G:p.N1834S	Neutral
					BRCA1:NM_007294:exon10:c.G3119A:p.S1040N	Deleterious
Sample_R12	**Proband: ovary c (35y).** Mother: endometrial c (79y). Maternal uncle 1: colon c (63y). Maternal uncle 2: prostate c (79y)	HNPCC, Amsterdam Criteria	MSH2/MSH6 LoE in proband’s tumor. MLH1, MSH2 & MSH6 genetic analysis negative	1) Lynch genes set	No candidate variant detected	
				2) Whole I2HCP	EXO1:NM_130398:c.2276G>A:p.G759E	Neutral
					HNF1A:NM_000545:exon4:c.C827G:p.A276G	Deleterious
					BRCA2:NM_000059:exon11:c.G4258T:p.D1420Y	Neutral
					PALB2:NM_024675:exon7:c.C2590T:p.P864S	Neutral
					PALB2:NM_024675:exon4:c.G232A:p.V78I	Neutral
					PRKAR1A:NM_002734:exon3:c.G221A:p.R74H	Deleterious
Sample_R13	**Proband: colon c (37y).** Father: colon c (57y). Grandfather: colon c (60y)	HNPCC, Amsterdam Criteria	MSH6 LoE in proband’s tumor. MLH1, MSH2 & MSH6 genetic analysis negative	1) Lynch genes set	POLE:NM_006231:exon42:c.A5797G:p.I1933V	Neutral
				2) Whole I2HCP	FANCD2:NM_001018115:exon28:c.G2702T:p.G901V	Neutral
					ATM:NM_000051:exon22:c.C3161G:p.P1054R	Deleterious
					PALB2:NM_024675:exon7:c.C2590T:p.P864S	Neutral
Sample_R14 (V39)	**Proband: Multiples schwannomas**	Schwannomatosis	NF2 and SMARCB1 genetic analysis negative	1) NF2 genes set	No candidate variant detected	
				2) Whole I2HCP	BARD1:NM_000465:exon11:c.A2146G:p.T716A	Neutral
					ERCC6:NM_000124:exon16:c.G2924A:p.R975Q	Deleterious
					SUFU:NM_016169:exon8:c.G1018T:p.A340S	Neutral
					ATM:NM_000051:exon17:c.T2572C:p.F858L	Neutral
					ATM:NM_000051:exon22:c.C3161G:p.P1054R	Deleterious
					FANCA:NM_000135:exon42:c.C4249G:p.H1417D	Deleterious
					FANCA:NM_000135:exon27:c.T2507A:p.F836Y	Neutral

Results of the re-analysis of 14 samples, including 4 samples from the validation set (V11, V30, V21 and V39). The I2HCP analysis encompasses the rare exonic non-synonymous variants detected in: 1) the genes included in the predefined gene group on the basis of clinical manifestations (Supplementary Table S1) and 2) the variants found in all genes of the panel. 1000G frequencies <0.01% have been used to filter rare variants. Variants highlighted in bold could be responsible for the developed phenotype.

Further analyses are needed to evaluate the impact of these variants. Functional prediction is based on data from Polyphen2 HDIV and HVAR, SIFT, PROVEAN and MutationTaster and a variant is marked as deleterious if three or more predictors classified it as damaging.
