# Supplementary material for: A comprehensive custom panel design for routine hereditary cancer testing: preserving control, improving diagnostics and revealing a complex variation landscape
Source: Sci Rep. 2017 Jan 4;7:39348. doi: 10.1038/srep39348 (PMC5209725; doi:10.1038/srep39348)
Supplement: Supplementary Information [file srep39348-s1.pdf]

SUPPLEMENTARY INFORMATION

A comprehensive custom panel design for routine hereditary cancer testing: preserving control, improving diagnostics and revealing a complex variation landscape

Elisabeth Castellanos[1#*], **Bernat Gel**[1#], Inma Rosas[1], Eva Tornero[2], Sheila Santín[3], Raquel Pluvinet[3], Juan Velasco[3], Lauro Sumoy[3], Jesús del Valle[2], Manuel Perucho[1], Ignacio Blanco[4], Matilde Navarro[2], Joan Brunet[2], Marta Pineda[2], Lidia Feliubadaló[2], Gabi Capellá[2*], Conxi Lázaro[2*], Eduard Serra[1*]

[1]Hereditary Cancer Program, The Institute for Health Science Research Germans Trias i Pujol (IGTP) - PMPPC, Can Ruti Campus, Badalona, Barcelona, Spain

[2]Translational Research Laboratory, Hereditary Cancer Program, Catalan Institute of Oncology (ICO-IDIBELL), L'Hospitalet de Llobregat, Barcelona, (ICO-IDIBGI), Girona, Spain

[3]Genomics and Bioinformatics Unit, IMPPC, Can Ruti Campus, Badalona, Barcelona, Spain

[4]Clinical Genetics and Genetic Counselling Program, Germans Trias i Pujol Hospital, Can Ruti Campus, Badalona, Barcelona, Spain

# Contributed equally to this work

* Corresponding authors: eserra@imppc.org; clazaro@iconcologia.net; gcapella@iconcologia.net; ecastellanos@imppc.org

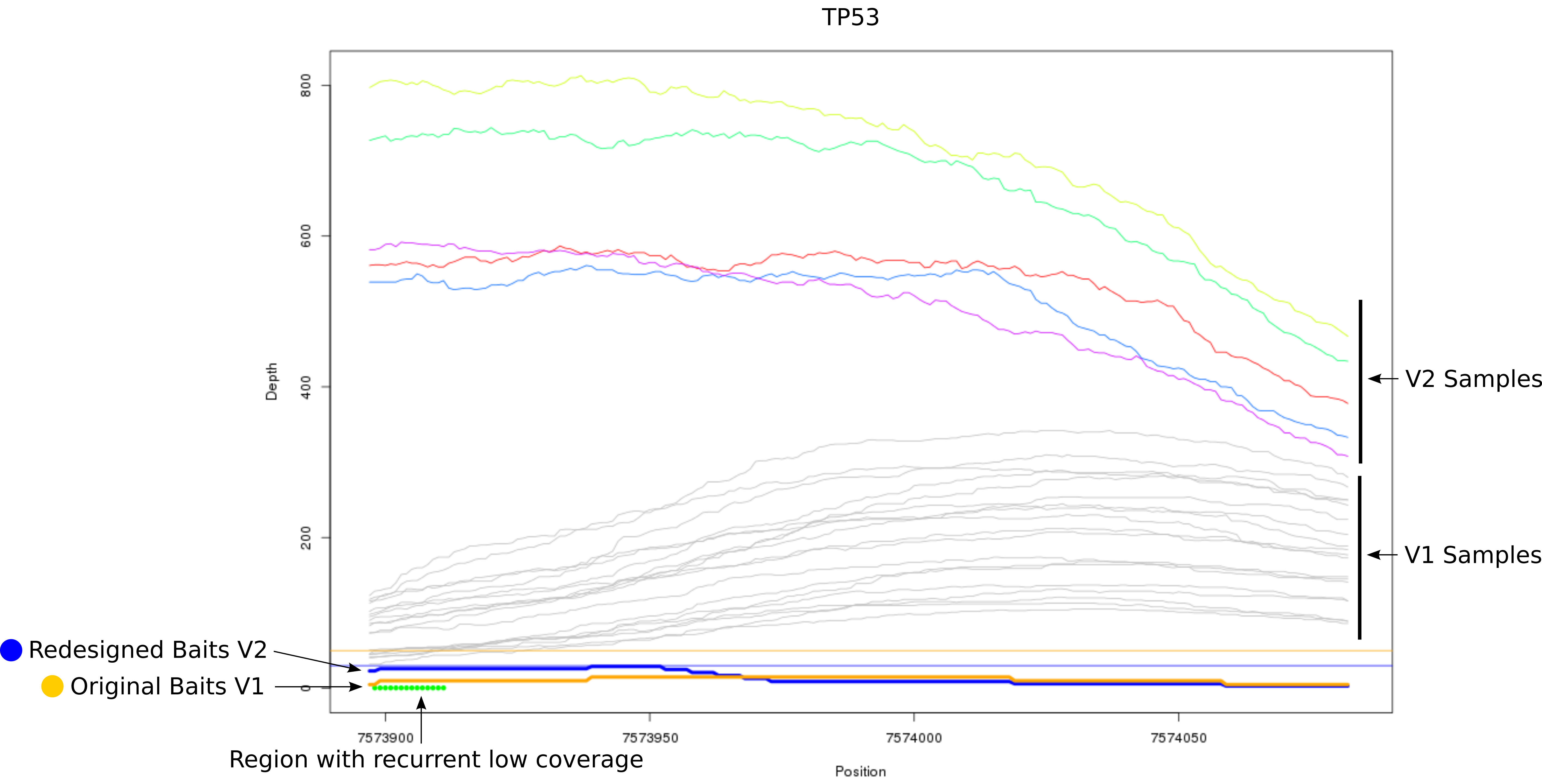
TP53
Depth
800
600
400
200
0
7573900
7573950
7574000
7574050
Position
V2 Samples
V1 Samples
Redesigned Baits V2
Original Baits V1
Region with recurrent low coverage

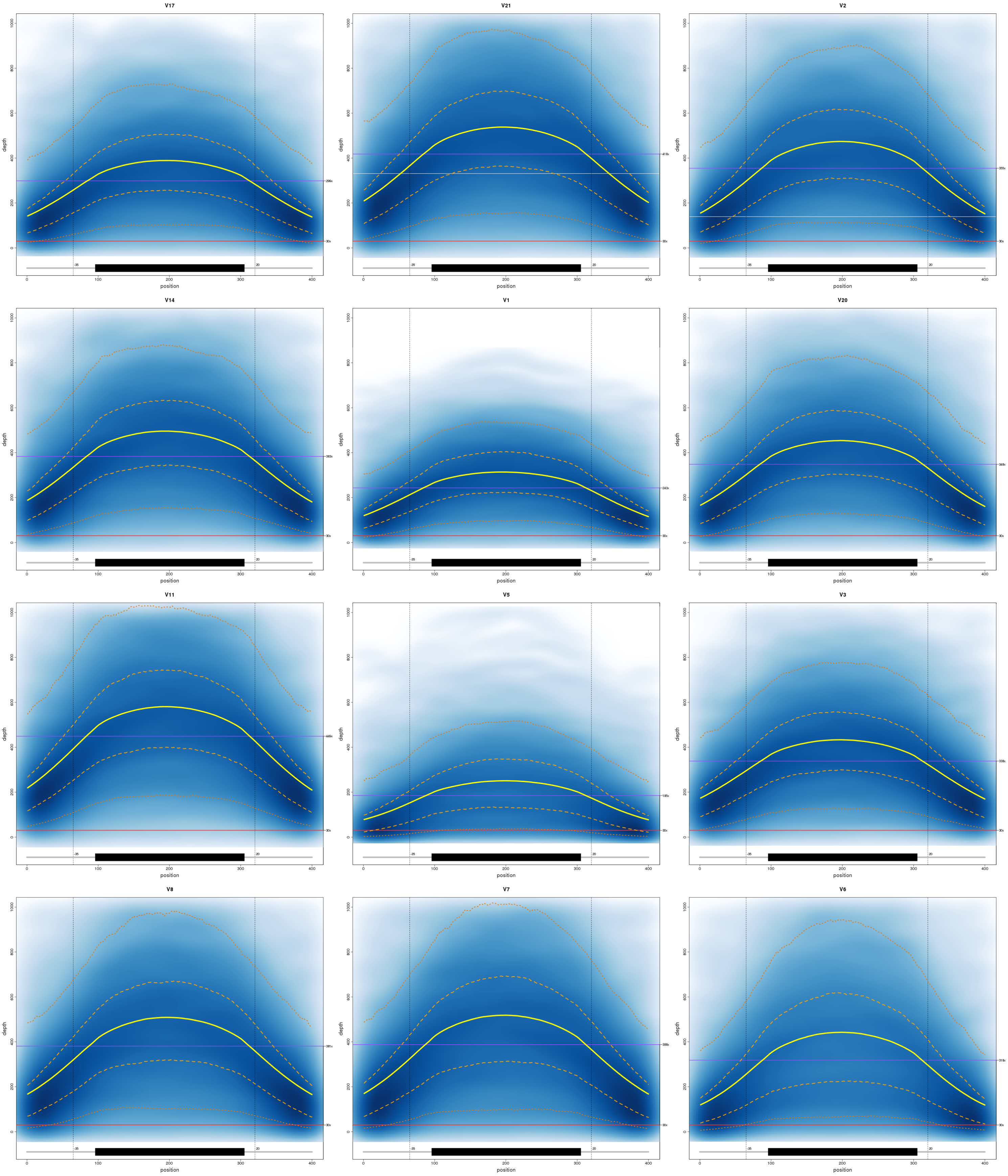
V17
V21
V2
V14
V1
V20
V11
V5
V3
V8
V7
V6
depth
position
0
100
200
300
400
0
200
400
600
800
1000

Supplementary Figure S3

A

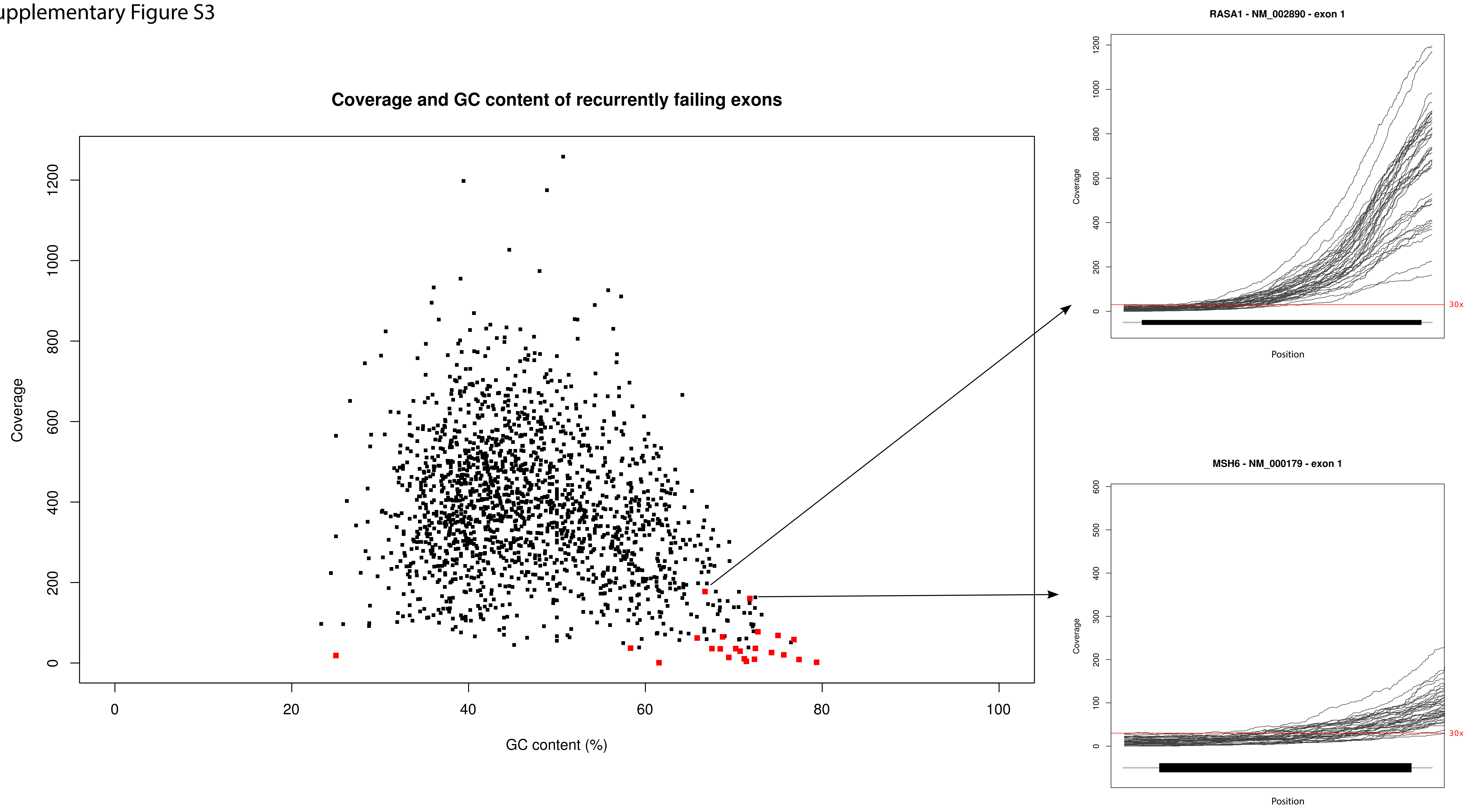

B

Gene	Transcript	Exon Rank	Ensembl Id	CDS Start	CDS End
BAP1	NM_004656	1	ENSE00001879355	1	37
BARD1	NM_000465	1	ENSE00002311538	1	158
BRAF	NM_004333	1	ENSE00001154485	1	138
CBL	NM_005188	1	ENSE00001240819	1	195
CDH1	NM_004360	1	ENSE00001859184	1	48
CDKN1C	NM_000076	1	ENSE00001691292	1	820
CDKN1C	NM_000076	2	ENSE00003634161	821	951
ERCC2	NM_000400	1	ENSE00001510142	1	5
FANCA	NM_000135	1	ENSE00002618162	1	79
FANCE	NM_021922	1	ENSE00000849631	1	248
FH	NM_000143	1	ENSE00001442032	1	132
MAP2K2	NM_030662	1	ENSE00001147081	1	92
MN1	NM_002430	1	ENSE00001150385	1	3781
MSH6	NM_000179	1	ENSE00002724201	1	260
NF1	NM_000267	1	ENSE00003598164	1	60
PDGFB	NM_002608	4	ENSE00000654697	251	456
PTCH1	NM_000264	1	ENSE00001630740	1	201
PTPN11	NM_002834	1	ENSE00001202658	1	14
RASA1	NM_002890	1	ENSE00001505330	1	539
RET	NM_020975	1	ENSE00001948565	1	73
TMEM127	NM_017849	2	ENSE00000921763	1	244
WT1	NM_024426	1	ENSE00001906049	1	646
XPA	NM_000380	1	ENSE00001465835	1	172

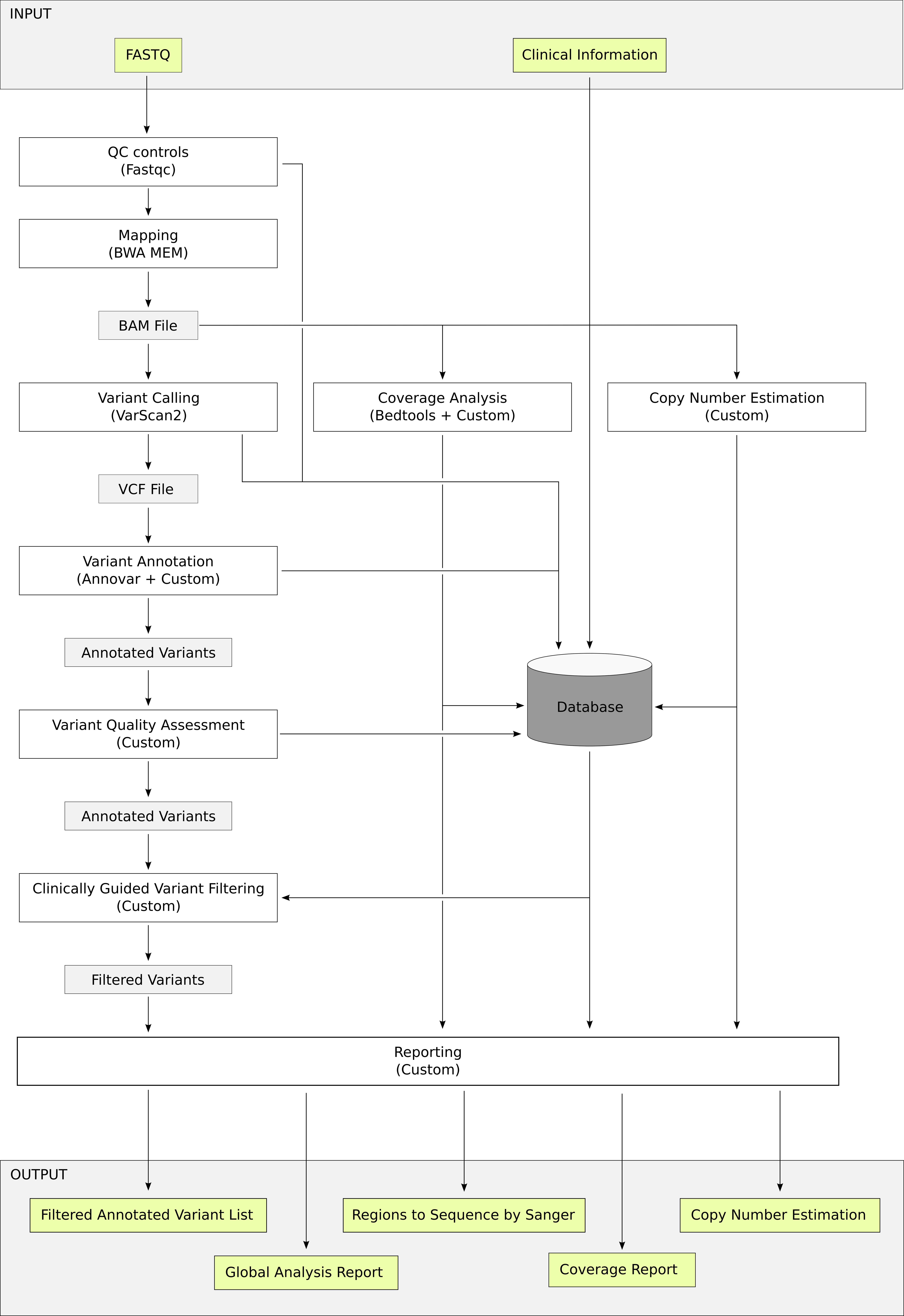
INPUT
FASTQ
Clinical Information
QC controls
(Fastqc)
Mapping
(BWA MEM)
BAM File
Variant Calling
(VarScan2)
Coverage Analysis
(Bedtools + Custom)
Copy Number Estimation
(Custom)
VCF File
Variant Annotation
(Annovar + Custom)
Annotated Variants
Database
Variant Quality Assessment
(Custom)
Annotated Variants
Clinically Guided Variant Filtering
(Custom)
Filtered Variants
Reporting
(Custom)
OUTPUT
Filtered Annotated Variant List
Regions to Sequence by Sanger
Copy Number Estimation
Global Analysis Report
Coverage Report

SUPPLEMENTARY FIGURE 5

Sample R2

a.Pedigree

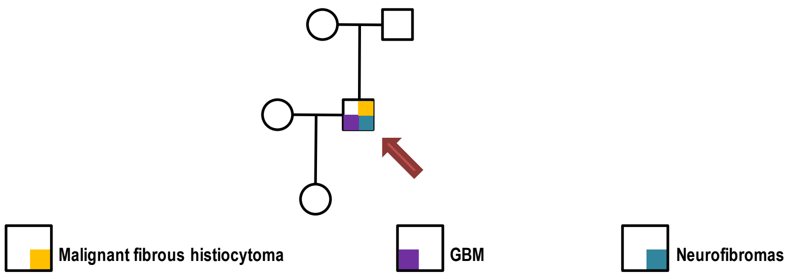

b.*CDKN2A* Normalized Coverage:

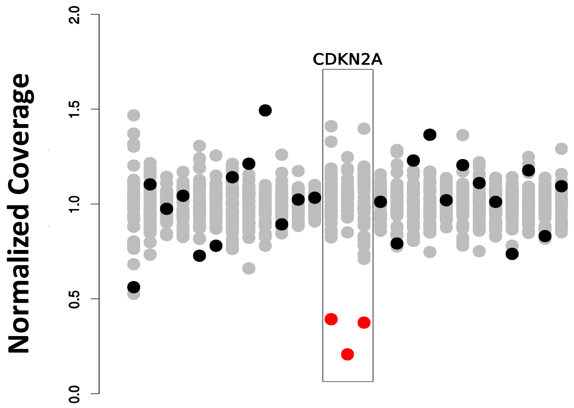

c.*CDKN2A* & *CDKN2B* deletion validated by MLPA:

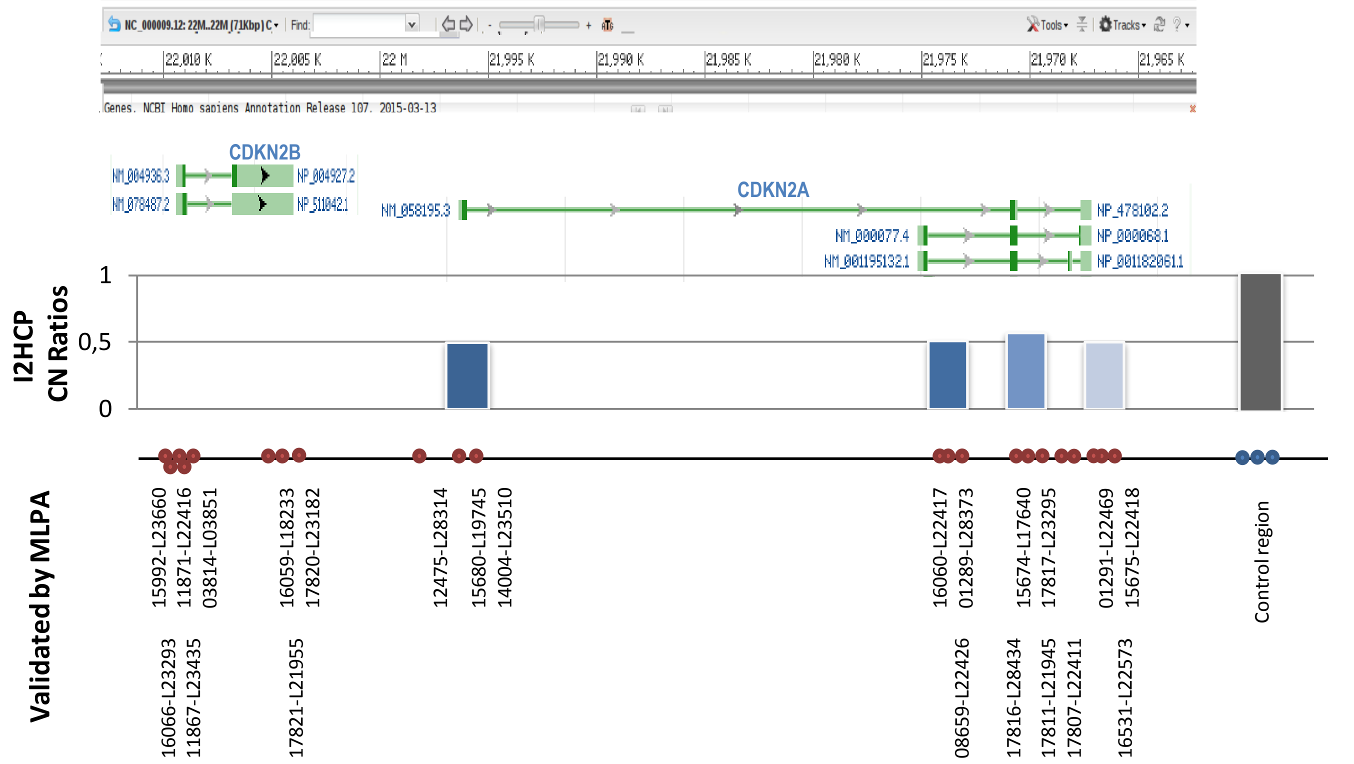

PATHOGENIC VARIANT TYPES

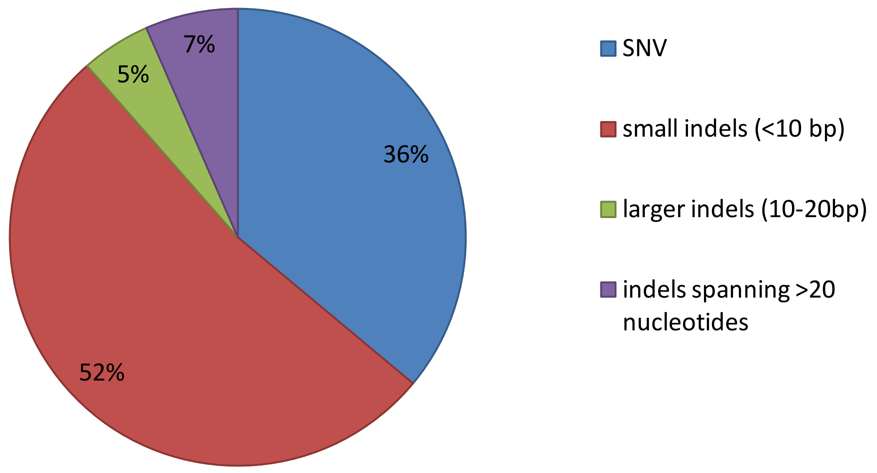

	HBOC	FAP	HNPCC	NF1	NF2
BRCA1	● (red)		●		
BRCA2	● (red)		●		
APC		● (red)	●		
MUTYH		● (red)	●		
MLH1	●		● (red)		
MSH2	●		● (red)		
MSH6	●		● (red)		
PMS2	●		● (red)		
NF1				● (red)	
NF2					● (red)
PTEN	●	●	●		
STK11	●	●	●		
EPCAM	●		●		
TP53	●		●		
ATM	●				
CDH1	●				
CHEK2	●				
PALB2	●				
RAD51C	●				
RAD51D	●				
POLD1		●	●		
POLE		●	●		
BMPR1A		●			
SMAD4		●			
SPRED1				●	
MN1					●
PDGFB					●
SMARCB1					●
SMARCE1					●
SUFU					●

Other Genes Included in the I2HCP:

AIP
ALK
ARAF
BAP1
BARD1
BLM
BRAF
BRIP1
BUB1B
CBL
CDC73
CDK4
CDKN1C
CDKN2A
CDKN2C
CYLD
DDB1
DDB2
DICER1
ELAC2
ERCC2
ERCC3
ERCC4
ERCC5
ERCC6
ERCC8
EXO1
EXT1
EXT2
FANCA
FANCB
FANCC
FANCD2
FANCE
FANCF
FANCG
FANCI
FANCL
FANCM
FH
FLCN
GPC3
HNF1A
HRAS
KIT
KLLN
KRAS
MAP2K1
MAP2K2
MAX
MEN1
MET
MLH3
MRE11A
MSH3
NBN
NRAS
PDGFRA
PHOX2B
PMS1
POLH
PPM1D
PRKAR1A
PRSS1
PTCH1
PTPN11
RAD50
RAD51
RAF1
RASA1
RB1
RET
RNASEL
SBDS
SDHAF2
SDHB
SDHC
SDHD
SHOC2
SLX4
SOS1
TGFBR2
TMEM127
TSC1
TSC2
TSHR
VHL
WRN
WT1
XPA
XPC
XRCC2

<u>Table S2</u>: Results from Training set (n=23)

Sample	Genes	Pathogenic Variant	Detected in 1st Approach	Detected in 2nd Approach	Variant Context
T1	*BRCA1*	NM_007294.2: c.2397T>A	YES	YES	
T2	*APC*	NM_001127510.2: c.3927_3931delAAAGA	YES	YES	
T3	*NF1*	NM_000267.3:c.7739C>G	YES	YES	
T4	*NF1*	NM_000267.3: c.3860_3861insTT	YES	YES	
T5	*BRCA1*	NM_007294.2:c.1961delA	NO	YES	HomoP
T6	*MLH1*	NM_000249.3: c.306+5A>G	YES	YES	
T7	*BRCA2*	NM_000059.3:c.956dupA	NO	YES	HomoP
T8	*MSH6*	NM_000179.2:c.3261delC	NO	YES	HomoP
T9	*NF1*	NM_000267.3:c.4306A>G	YES	YES	
T10	*MSH6*	NM_000179.2:c.255dupC	NO	NO	Low coverage
T11	*NF1*	NM_000267.3: Del exons 33-36	YES	YES	
T12	*NF2*	NM_000268.3: Del exons 1-5	YES	YES	
T13	*NF1*	NM_000267.3:c.6365-2A>G	YES	YES	
T14	*APC*	NM_001127510.2:c.423-3T>A	YES	YES	
T15	*MSH2*	NM_000251.2:c.602_603insT	NO	YES	HomoP
T16	*BRCA2*	NM_000059.3:c.8946delA	NO	YES	HomoP
T17	*BRCA2*	NM_000059.3:c.2402_2420del19	YES	YES	
T18	*BRCA2*	NM_000059.3:c.6275_6276delTT	YES	YES	
T19	*MSH6*	NM_000179.2:c.3150_3161dup12	YES	YES	
T20	*MSH6*	NM_000179.2:c.3261dupC	NO	YES	HomoP
T21	*MLH1*	NM_000249.3:c.1590_1598dup9	YES	YES	
T22	*NF1*	NM_000267.3:c.6226delG	YES	YES	
T23	*BRCA1*	NM_007294.2:c.1953_1956delGAAA	YES	YES	

sample	totalReads	pctMappedReads	properlyPairedReads	meanCoverage	medianCoverage	maxCoverage	C1	C10	C30	C60	C100	U20	U50
T1	2700557	99.89%	98.48%	458.3894277	442	1443	99.93%	99.78%	99.35%	98.42%	96.72%	0.66%	0.28%
T2	3620541	99.90%	98.90%	601.7126241	580	2052	99.96%	99.82%	99.41%	98.89%	97.53%	0.67%	0.28%
T3	2971530	99.90%	98.87%	496.6900251	476	1741	99.97%	99.75%	99.15%	98.11%	96.55%	0.68%	0.30%
T4	3080176	99.90%	98.97%	557.5283568	535	1886	99.90%	99.73%	99.28%	98.46%	97.08%	0.69%	0.31%
T5	2932666	99.22%	96.18%	336.7794168	325	1236	99.92%	99.68%	98.75%	96.89%	93.72%	0.68%	0.31%
T6	2948395	99.89%	97.81%	270.3864092	256	1087	99.59%	98.82%	97.39%	94.58%	88.94%	0.70%	0.34%
T7	2974347	99.80%	97.34%	483.3309917	465	1679	99.94%	99.69%	99.18%	98.21%	96.27%	0.68%	0.30%
T8	4030929	98.80%	95.68%	579.8089141	558	2152	99.97%	99.69%	99.27%	98.24%	96.68%	0.69%	0.31%
T9	6529562	99.04%	97.86%	1086.624322	1051	3512	99.99%	99.83%	99.61%	99.21%	98.53%	0.69%	0.30%
T10	2937637	99.75%	97.56%	398.1105709	382	1500	99.87%	99.68%	99.03%	97.58%	95.35%	0.68%	0.31%
T11	3455356	99.95%	98.73%	155.0875564	149	579	99.65%	98.52%	96.55%	90.25%	75.71%	0.68%	0.31%
T12	3454764	99.91%	98.69%	526.7052471	508	1792	99.98%	99.78%	99.26%	98.42%	96.87%	0.68%	0.29%
T13	3157978	99.95%	98.83%	438.9549286	422	1609	99.55%	98.83%	97.91%	96.69%	94.56%	0.70%	0.34%
T14	3040733	99.38%	95.45%	481.469552	463	1725	99.86%	99.69%	99.12%	98.28%	96.28%	0.68%	0.30%
T15	2491293	99.55%	97.02%	409.6304417	395	1465	99.89%	99.66%	99.12%	97.69%	95.47%	0.68%	0.30%
T16	4103602	99.37%	97.17%	447.420466	426	1672	99.93%	99.60%	98.93%	97.58%	95.60%	0.69%	0.31%
T17	4101591	99.32%	97.40%	646.1095886	621	2398	99.92%	99.79%	99.37%	98.63%	97.24%	0.69%	0.31%
T18	2823557	99.55%	97.95%	440.1877041	424	1643	99.95%	99.69%	98.93%	97.72%	95.78%	0.69%	0.31%
T19	4366424	99.70%	98.02%	635.1975054	605	2428	99.99%	99.77%	99.33%	98.52%	97.20%	0.69%	0.32%
T20	3517122	99.94%	98.95%	512.7125471	496	1768	99.92%	99.76%	99.25%	98.36%	96.79%	0.67%	0.30%
T21	3414029	99.84%	98.44%	489.5334775	472	1741	99.90%	99.76%	99.26%	98.20%	96.49%	0.67%	0.30%
T22	2852050	99.41%	98.01%	506.8352003	484	2014	99.90%	99.64%	99.17%	98.19%	96.49%	0.69%	0.32%
T23	2702987	99.59%	97.87%	432.9446815	413	1649	99.91%	99.70%	99.05%	97.76%	95.75%	0.69%	0.31%
V1	3120052	99.94%	98.80%	295.6665951	290	842	99.95%	99.52%	98.55%	96.75%	93.33%	0.66%	0.27%
V2	3102542	99.91%	98.89%	437.7827133	414	1910	99.82%	99.33%	98.53%	97.27%	94.89%	0.70%	0.32%
V3	3184166	99.93%	98.94%	412.2868723	400	1354	99.95%	99.75%	99.16%	97.93%	95.95%	0.68%	0.29%
V4	3564282	99.73%	98.73%	566.3987475	534	2428	99.83%	99.41%	98.60%	97.57%	96.07%	0.71%	0.34%
V5	2324799	99.66%	98.38%	234.0922369	217	1019	99.84%	98.74%	95.74%	89.85%	80.52%	0.76%	0.44%
V6	2762570	99.71%	98.75%	409.2610649	376	1933	99.81%	99.08%	97.43%	95.03%	90.66%	0.77%	0.46%
V7	3389493	99.36%	97.71%	490.9416272	460	2102	99.81%	99.19%	98.19%	96.77%	94.31%	0.72%	0.38%
V8	2898965	99.68%	98.58%	480.3903083	454	1917	99.81%	99.24%	98.23%	96.98%	94.55%	0.72%	0.36%
V9	3022540	99.66%	97.68%	180.8694414	174	645	99.79%	99.28%	97.32%	92.81%	82.18%	0.68%	0.30%
V10	3142118	99.82%	98.55%	412.5336949	402	1243	99.89%	99.59%	99.07%	97.95%	96.13%	0.67%	0.28%
V11	3348711	99.61%	97.56%	539.0104176	523	1576	99.98%	99.84%	99.38%	98.67%	97.39%	0.67%	0.28%
V12	3815649	99.73%	97.82%	114.4278238	111	390	99.82%	98.79%	95.11%	83.63%	56.84%	0.68%	0.30%
V13	3202154	99.68%	97.92%	483.2600551	473	1444	99.96%	99.77%	99.24%	98.34%	96.67%	0.67%	0.28%
V14	3118424	99.15%	96.59%	463.5094917	451	1366	99.97%	99.70%	99.25%	98.31%	96.60%	0.66%	0.28%
V15	3310405	99.77%	97.91%	515.3704581	493	1820	99.94%	99.66%	99.12%	98.18%	96.73%	0.69%	0.31%
V16	2675671	99.86%	98.01%	393.4459817	377	1546	99.95%	99.63%	98.73%	97.32%	94.81%	0.69%	0.31%
V17	3121897	99.88%	97.46%	366.6039986	349	1422	99.87%	99.48%	98.62%	97.06%	94.23%	0.69%	0.32%
V18	3209613	99.88%	98.22%	497.52831	478	1793	99.96%	99.61%	99.01%	97.96%	96.28%	0.68%	0.31%
V19	1655353	99.83%	97.31%	237.1547783	226	836	99.89%	99.23%	97.88%	94.76%	87.70%	0.70%	0.33%
V20	2735448	99.76%	97.67%	425.3410158	409	1547	99.88%	99.57%	98.91%	97.62%	95.57%	0.68%	0.31%
V21	3343120	99.85%	97.90%	509.2725969	494	1663	99.95%	99.68%	99.22%	98.21%	96.82%	0.68%	0.30%
V22	5416213	98.65%	96.47%	272.6839881	263	1017	99.92%	99.51%	98.33%	96.14%	91.34%	0.68%	0.30%
V23	4263438	99.94%	98.48%	208.4556151	200	725	99.84%	99.32%	97.58%	93.85%	85.93%	0.68%	0.31%
V24	3250856	99.92%	98.46%	401.8487658	386	1443	99.85%	99.65%	99.26%	97.93%	95.44%	0.68%	0.29%
V25	3511443	99.93%	98.70%	445.2353951	426	1590	99.97%	99.77%	99.19%	98.06%	96.08%	0.67%	0.30%
V26	3815216	99.94%	98.73%	624.4534936	602	2206	99.99%	99.83%	99.39%	98.84%	97.75%	0.67%	0.29%
V27	3403384	99.94%	98.40%	397.4319897	382	1351	99.93%	99.71%	99.12%	97.99%	95.54%	0.67%	0.29%
V28	4403274	99.92%	98.60%	727.3731052	704	2487	99.93%	99.76%	99.55%	99.10%	98.20%	0.67%	0.28%
V29	2993755	99.94%	98.13%	209.4945422	202	704	99.85%	99.36%	97.89%	94.44%	86.80%	0.68%	0.29%
V30	3135958	99.94%	98.64%	465.2157651	448	1693	99.93%	99.74%	99.12%	98.16%	96.31%	0.67%	0.29%
V31	2925995	99.93%	98.61%	273.4182949	256	1057	99.94%	99.41%	98.08%	95.46%	89.72%	0.70%	0.34%
V32	3160863	99.93%	97.03%	235.1366617	221	934	99.84%	99.20%	97.47%	94.15%	86.72%	0.71%	0.35%
V33	3427158	99.85%	92.68%	493.8036751	475	1685	99.92%	99.75%	99.18%	98.22%	96.56%	0.68%	0.30%
V34	3248449	99.92%	98.92%	493.7740374	475	1732	99.94%	99.74%	99.31%	98.32%	96.51%	0.68%	0.30%
V35	2826057	99.94%	99.05%	387.3789909	373	1447	99.85%	99.45%	98.83%	97.71%	95.29%	0.67%	0.29%
V36	2551495	99.89%	98.71%	253.8151548	244	1015	99.78%	99.21%	98.14%	95.76%	90.67%	0.68%	0.29%
V37	3427675	99.91%	98.60%	135.1547646	131	469	99.44%	98.35%	95.37%	86.84%	68.07%	0.69%	0.32%
V38	2648692	99.67%	98.59%	397.9490951	378	1483	99.71%	99.10%	98.08%	96.71%	94.14%	0.71%	0.34%
V39	2771123	99.94%	99.22%	459.7732505	435	1881	99.83%	99.24%	98.35%	97.28%	95.34%	0.70%	0.33%
V40	3422016	99.93%	99.23%	575.9554321	548	2453	99.90%	99.48%	98.78%	98.00%	96.60%	0.70%	0.32%

Sample Name	Variants in analyzed genes detected with I2HCP V2	Rs (Snp138)	Variant Freq	Status	Reason to be undetected by pre-NGS methods	Validated by Sanger	Variants also detected in I2HCP V1 (PGM)
TRAINING							
T1							
	BRCA2:NM_000059:c.425+67A>C	rs11571610	56,27	Known			YES
	BRCA2:NM_000059:c.425+147G>T	rs4942423	44,83		Out of pre-NGS method covered region		
	BRCA2:NM_000059:c.426-89T>C	rs3783265	55,11	Known			YES
	BRCA2:NM_000059:c.631+183T>A	rs3752451	48,57		Out of pre-NGS method covered region		
	BRCA2:NM_000059:c.681+56C>T	rs2126042	49,3		Out of pre-NGS method covered region		
	BRCA2:NM_000059:c.793+98G>A	rs206073	99,24		Out of pre-NGS method covered region		
	BRCA2:NM_000059:c.865A>C	rs766173	46,41	Known			YES
	BRCA2:NM_000059:c.1365A>G	rs1801439	36,47	Known			YES
	BRCA2:NM_000059:c.1910-74T>C	rs2320236	48,62		Out of pre-NGS method covered region		
	BRCA2:NM_000059:c.1910-51G>T	rs11571651	49,87		Out of pre-NGS method covered region		
	BRCA2:NM_000059:c.2229T>C	rs1801499	48,6	Known			YES
	BRCA2:NM_000059:c.2971A>G	rs1799944	52,22	Known			YES
	BRCA2:NM_000059:c.4563A>G	rs206075	100		Could not be detected by CSCE		
	BRCA2:NM_000059:c.6513G>C	rs206076	100		Could not be detected by CSCE		
	BRCA2:NM_000059:c.6937+151A>G	rs4942439	41,38		Out of pre-NGS method covered region		
	BRCA2:NM_000059:c.6937+177G>A	rs4942440	41,67		Out of pre-NGS method covered region		
	BRCA2:NM_000059:c.6938-120T>C	rs206080	100		Out of pre-NGS method covered region		
	BRCA2:NM_000059:c.7397T>C	rs169547	100		Could not be detected by CSCE		
	BRCA2:NM_000059:c.7435+53C>T	rs11147489	48,15	Known			YES
	BRCA2:NM_000059:c.7617+190G>A	rs206096	100		Out of pre-NGS method covered region		
	BRCA2:NM_000059:c.7806-14T>C	rs9534262	100	Known			YES
	BRCA2:NM_000059:c.8755-66T>C	rs4942486	99,57		Out of pre-NGS method covered region		
	BRCA2:NM_000059:c.9648+244T>C	rs206344	100		Out of pre-NGS method covered region		
	BRCA1:NM_007294:c.5407-193A>G	rs8176310	62,86		Out of pre-NGS method covered region		
	BRCA1:NM_007294:c.5278-191A>T	rs8176297	33,78		Out of pre-NGS method covered region		
	BRCA1:NM_007294:c.5152+66G>A	rs3092994	49,71		Out of pre-NGS method covered region		
	BRCA1:NM_007294:c.5144G>A	rs45444999	51,29	Known			YES
	BRCA1:NM_007294:c.5075-237C>A	rs8176257	66,67		Out of pre-NGS method covered region		
	BRCA1:NM_007294:c.5074+65G>A	rs8176235	51,77	Known			YES
	BRCA1:NM_007294:c.4987-68A>G	rs8176234	48,32		Out of pre-NGS method covered region		
	BRCA1:NM_007294:c.4987-92A>G	rs8176233	46,79		Out of pre-NGS method covered region		
	BRCA1:NM_007294:c.4986+222A>G	rs3092987	71,43		Out of pre-NGS method covered region		
	BRCA1:NM_007294:c.4837A>G	rs1799966	53,83	Known			YES
	BRCA1:NM_007294:c.4485-63C>G	rs8176212	52,48	Known			YES
	BRCA1:NM_007294:c.4485-137T>A	rs2236762	47,32		Out of pre-NGS method covered region		
	BRCA1:NM_007294:c.4358-2590T>G	rs8176194	45,41		Out of pre-NGS method covered region		
	BRCA1:NM_007294:c.4358-2885G>A	rs8176193	47,98		Out of pre-NGS method covered region		
	BRCA1:NM_007294:c.4308T>C	rs1060915	50,43	Known			YES
	BRCA1:NM_007294:c.4097-141A>C	rs799916	50,83		Out of pre-NGS method covered region		
	BRCA1:NM_007294:c.3548A>G	rs16942	41,54	Known			YES
	BRCA1:NM_007294:c.3113A>G	rs16941	50,27	Known			YES
	BRCA1:NM_007294:c.2612C>T	rs799917	50,78	Known			YES
	**BRCA1:NM_007294:c.2397T>A**	**rs80357203**	**52,11**	**PAT**			YES
	BRCA1:NM_007294:c.2311T>C	rs16940	51,76	Known			YES
	BRCA1:NM_007294:c.2082C>T	rs1799949	49,38	Known			YES
	BRCA1:NM_007294:c.594-225G>A	rs8176147	45,83		Out of pre-NGS method covered region		
	BRCA1:NM_007294:c.593+167T>C	rs8176145	45,45	Known			YES
	BRCA1:NM_007294:c.547+146A>T	rs8176140	36,67		Out of pre-NGS method covered region		
	BRCA1:NM_007294:c.213-161A>G	rs799912	55,56		Out of pre-NGS method covered region		
	BRCA1:NM_007294:c.1-134T>C	rs3765640	46,97	Known			YES
	BRCA1:NM_007294:c.4485-203_4485-199delAACCC	rs34250703	30.77		Out of pre-NGS method covered region		
	BRCA1:NM_007294:c.548-58_548-58delT	rs8176144	45.14		Out of pre-NGS method covered region		
	BRCA1:NM_007294:c.441+64_441+64delT	rs368252296	43.55		Out of pre-NGS method covered region		
	BRCA1:NM_007294:c.441+52_441+63delCTTTTTTTTTTT	.	81.33		Out of pre-NGS method covered region		

	BRCA1:NM_007294:c.441+36_441+38delCTT	rs147856441	45.96		Out of pre-NGS method covered region		
	BRCA1:NM_007294:c.134+224_134+224delT	rs35149296	52.94		Out of pre-NGS method covered region		
T2							
	APC:NM_001127510:c.136-230C>A	rs2464805	100		Out of pre-NGS method covered region		
	APC:NM_001127510:c.423-256G>A	rs392179	100		Out of pre-NGS method covered region		
	APC:NM_001127510:c.645+173A>G	rs2289484	100		Out of pre-NGS method covered region		
	APC:NM_001127510:c.933+261G>T	rs12656359	100		Out of pre-NGS method covered region		
	APC:NM_001127510:c.934-132G>T	rs12521276	100		Out of pre-NGS method covered region		
	APC:NM_001127510:c.1458T>C	rs2229992	99,76		Out of pre-NGS method covered region		
	APC:NM_001127510:c.1635G>A	rs351771	99,91		Out of pre-NGS method covered region		
	APC:NM_001127510:c.1743+193G>A	rs351772	100		Out of pre-NGS method covered region		
	APC:NM_001127510:c.4479G>A	rs41115	99,81		Out of pre-NGS method covered region		
	APC:NM_001127510:c.5034G>A	rs42427	99,82		Out of pre-NGS method covered region		
	APC:NM_001127510:c.5268T>G	rs866006	100		Out of pre-NGS method covered region		
	APC:NM_001127510:c.5465T>A	rs459552	99,55		Out of pre-NGS method covered region		
	APC:NM_001127510:c.5880G>A	rs465899	100		Out of pre-NGS method covered region		
	APC:NM_001127510:c.934-132_934-132delG	rs141013063	58.82		Out of pre-NGS method covered region		
	APC:NM_001127510:c.934-120_934-119insG	rs138003874	78.99		Out of pre-NGS method covered region		
	APC:NM_001127510:c.1959-145_1959-144insAGAA	rs3839284	98.57		Out of pre-NGS method covered region		
	**APC:NM_001127510:c.3921_3925delAAAAG**	**.**	**44.81**	**PAT**			YES
T3							
	NF1:NM_000267:c.61-300C>T	rs2269856	100		Out of pre-NGS method covered region		
	NF1:NM_000267:c.61-123G>A	rs2269855	100		Out of pre-NGS method covered region		
	NF1:NM_000267:c.288+41G>A	rs2952976	99,74		Could not be detected by RNA-based analysis		
	NF1:NM_000267:c.480-90C>T	rs2905807	99,17		Out of pre-NGS method covered region		
	NF1:NM_000267:c.702G>A	rs1801052	98,16	Known			YES
	NF1:NM_000267:c.888+108C>T	rs2953000	100		Out of pre-NGS method covered region		
	NF1:NM_000267:c.888+118G>T	rs2952999	100		Out of pre-NGS method covered region		
	NF1:NM_000267:c.1393-130A>T	rs2905875	100		Out of pre-NGS method covered region		
	NF1:NM_000267:c.1393-32T>C	rs2905876	99,69		Could not be detected by RNA-based analysis		
	NF1:NM_000267:c.1641+39T>C	rs2905880	99,76		Could not be detected by RNA-based analysis		
	NF1:NM_000267:c.5205+23T>C	rs9894648	100		Could not be detected by RNA-based analysis		
	NF1:NM_000267:c.5546+19T>A	rs2285894	99,42		Could not be detected by RNA-based analysis		
	NF1:NM_000267:c.5546+117G>A	rs3815154	100		Out of pre-NGS method covered region		
	NF1:NM_000267:c.6085-29T>A	rs7406038	90,72		Could not be detected by RNA-based analysis		
	NF1:NM_000267:c.6085-28T>A	rs7406039	99,32		Could not be detected by RNA-based analysis		
	NF1:NM_000267:c.7126+37C>G	rs7405740	100		Could not be detected by RNA-based analysis		
	NF1:NM_000267:c.7127-167T>A	rs2525569	100		Out of pre-NGS method covered region		
	NF1:NM_000267:c.7395-29G>A	rs964288	100		Could not be detected by RNA-based analysis		
	**NF1:NM_000267:c.7739C>G**	**.**	**48,72**	**PAT**			YES
	NF1:NM_000267:c.7907+166C>T	rs7350943	100		Could not be detected by RNA-based analysis		
	NF1:NM_000267:c.730+15_730+16insT	.	25.39		Could not be detected by RNA-based analysis		
	NF1:NM_000267:c.889-191_889-190insT	rs35966725	100		Out of pre-NGS method covered region		
	NF1:NM_000267:c.1528-37_1528-36insT	rs67472948	94.06		Could not be detected by RNA-based analysis		
	NF1:NM_000267:c.2002-118_2002-117insAC	rs141114572	39.71		Out of pre-NGS method covered region		
T4							
	NF1:NM_000267:c.61-300C>T	rs2269856	100		Out of pre-NGS method covered region		
	NF1:NM_000267:c.61-123G>A	rs2269855	99,86		Out of pre-NGS method covered region		
	NF1:NM_000267:c.288+41G>A	rs2952976	99,53		Could not be detected by RNA-based analysis		
	NF1:NM_000267:c.480-90C>T	rs2905807	100		Could not be detected by RNA-based analysis		
	NF1:NM_000267:c.702G>A	rs1801052	98,91	Known			YES
	NF1:NM_000267:c.888+108C>T	rs2953000	100		Out of pre-NGS method covered region		
	NF1:NM_000267:c.888+118G>T	rs2952999	100		Out of pre-NGS method covered region		
	NF1:NM_000267:c.1393-130A>T	rs2905875	100		Out of pre-NGS method covered region		
	NF1:NM_000267:c.1393-32T>C	rs2905876	100		Could not be detected by RNA-based analysis		
	NF1:NM_000267:c.1641+39T>C	rs2905880	99,82		Could not be detected by RNA-based analysis		
	NF1:NM_000267:c.1726C>G	.	48,55	Known			YES
	NF1:NM_000267:c.5205+23T>C	rs9894648	100		Could not be detected by RNA-based analysis		

	NF1:NM_000267:c.5546+19T>A	rs2285894	99,54		Could not be detected by RNA-based analysis		
	NF1:NM_000267:c.5546+117G>A	rs3815154	100		Out of pre-NGS method covered region		
	NF1:NM_000267:c.6085-29T>A	rs7406038	90,16		Could not be detected by RNA-based analysis		
	NF1:NM_000267:c.6085-28T>A	rs7406039	95,56		Could not be detected by RNA-based analysis		
	NF1:NM_000267:c.7126+37C>G	rs7405740	99,51		Could not be detected by RNA-based analysis		
	NF1:NM_000267:c.7127-167T>A	rs2525569	100		Out of pre-NGS method covered region		
	NF1:NM_000267:c.7395-29G>A	rs964288	100		Could not be detected by RNA-based analysis		
	NF1:NM_000267:c.7907+166C>T	rs7350943	100		Out of pre-NGS method covered region		
	NF1:NM_000267:c.730+15_730+16insT	.	27.34		Could not be detected by RNA-based analysis		
	NF1:NM_000267:c.889-191_889-190insT	rs35966725	100		Out of pre-NGS method covered region		
	NF1:NM_000267:c.1528-37_1528-36insT	rs67472948	96.02		Could not be detected by RNA-based analysis		
	NF1:NM_000267:c.2002-118_2002-117insAC	rs141114572	44.44		Out of pre-NGS method covered region		
	**NF1:NM_000267:c.3860_3861insTT**	**.**	**44.39**	**PAT**	Could not be detected by RNA-based analysis		YES
	NF1:NM_000267:c.6085-31_6085-30insA	.	34.49		Could not be detected by RNA-based analysis		
T5							
	BRCA2:NM_000059:c.793+98G>A	rs206073	100		Out of pre-NGS method covered region		
	BRCA2:NM_000059:c.3807T>C	rs543304	99,27	Known			YES
	BRCA2:NM_000059:c.4563A>G	rs206075	99,42		Could not be detected by CSCE		
	BRCA2:NM_000059:c.6513G>C	rs206076	99,62		Could not be detected by CSCE		
	BRCA2:NM_000059:c.6938-120T>C	rs206080	98,97		Out of pre-NGS method covered region		
	BRCA2:NM_000059:c.7397T>C	rs169547	99,81		Could not be detected by CSCE		
	BRCA2:NM_000059:c.7435+207T>A	.	57,14		Out of pre-NGS method covered region		
	BRCA2:NM_000059:c.7617+190G>A	rs206096	100		Out of pre-NGS method covered region		
	BRCA2:NM_000059:c.9501+178A>G	rs206343	100		Out of pre-NGS method covered region		
	BRCA2:NM_000059:c.10257+105A>C	rs15869	100		Out of pre-NGS method covered region		
	BRCA1:NM_007294:c.5407-193A>G	rs8176310	100		Out of pre-NGS method covered region		
	BRCA1:NM_007294:c.5333-153A>G	rs8176305	40,54		Out of pre-NGS method covered region		
	BRCA1:NM_007294:c.5278-191A>T	rs8176297	100		Out of pre-NGS method covered region		
	BRCA1:NM_007294:c.5152+66G>A	rs3092994	100		Out of pre-NGS method covered region		
	BRCA1:NM_007294:c.5075-53C>T	rs8176258	57,73	Known			YES
	BRCA1:NM_007294:c.5075-237C>A	rs8176257	60		Out of pre-NGS method covered region		
	BRCA1:NM_007294:c.5075-238A>G	rs8176256	35		Out of pre-NGS method covered region		
	BRCA1:NM_007294:c.5074+65G>A	rs8176235	44,9	Known			YES
	BRCA1:NM_007294:c.4987-68A>G	rs8176234	100		Out of pre-NGS method covered region		
	BRCA1:NM_007294:c.4987-92A>G	rs8176233	97,96		Out of pre-NGS method covered region		
	BRCA1:NM_007294:c.4986+222A>G	rs3092987	100		Out of pre-NGS method covered region		
	BRCA1:NM_007294:c.4837A>G	rs1799966	99,3	Known			YES
	BRCA1:NM_007294:c.4675+245G>A	rs141660808	53,85		Out of pre-NGS method covered region		
	BRCA1:NM_007294:c.4485-63C>G	rs8176212	98,75	Known			YES
	BRCA1:NM_007294:c.4485-137T>A	rs2236762	100		Out of pre-NGS method covered region		
	BRCA1:NM_007294:c.4358-2590T>G	rs8176194	100		Out of pre-NGS method covered region		
	BRCA1:NM_007294:c.4358-2885G>A	rs8176193	99,51		Out of pre-NGS method covered region		
	BRCA1:NM_007294:c.4357+117G>A	rs3737559	49,25		Out of pre-NGS method covered region		
	BRCA1:NM_007294:c.4308T>C	rs1060915	99,54	Known			YES
	BRCA1:NM_007294:c.4097-141A>C	rs799916	99,14		Could not be detected by CSCE		
	BRCA1:NM_007294:c.3548A>G	rs16942	99,28	Known			YES
	BRCA1:NM_007294:c.3113A>G	rs16941	99,14	Known			YES
	BRCA1:NM_007294:c.2612C>T	rs799917	99,61	Known			YES
	BRCA1:NM_007294:c.2311T>C	rs16940	99,28	Known			YES
	BRCA1:NM_007294:c.2082C>T	rs1799949	99,73	Known			YES
	BRCA1:NM_007294:c.2077G>A	rs4986850	49,54	Known			YES
	BRCA1:NM_007294:c.594-225G>A	rs8176147	100		Out of pre-NGS method covered region		
	BRCA1:NM_007294:c.593+167T>C	rs8176145	100	Known			YES
	BRCA1:NM_007294:c.547+146A>T	rs8176140	100		Out of pre-NGS method covered region		
	BRCA1:NM_007294:c.441+36C>T	rs45569832	62,5		Out of pre-NGS method covered region		
	BRCA1:NM_007294:c.213-161A>G	rs799912	100		Out of pre-NGS method covered region		
	BRCA1:NM_007294:c.1-134T>C	rs3765640	100	Known			YES
	BRCA1:NM_007294:c.4485-203_4485-199delAACCC	rs34250703	54.39		Out of pre-NGS method covered region		

	**BRCA1:NM_007294:c.1961_1961delA**	**rs80357522**	**43.32**	**PAT**			NO, variant in homopolymer region
	BRCA1:NM_007294:c.548-58_548-58delT	rs8176144	92.95		Out of pre-NGS method covered region		
	BRCA1:NM_007294:c.441+52_441+63delCTTTTTTTTTTT	.	95.71		Out of pre-NGS method covered region		
	BRCA1:NM_007294:c.441+36_441+38delCTT	rs147856441	97.97		Out of pre-NGS method covered region		
T6							
	**MLH1:NM_000249:c.306+5G>A**	**rs267607735**	**46,33**	**PAT**			YES
	MLH1:NM_000249:c.453+79A>G	rs4234259	100		Out of pre-NGS method covered region		
	MLH1:NM_000249:c.655A>G	rs1799977	45,41	Known			YES
	MLH1:NM_000249:c.1038+86T>C	rs2286939	100	Known			YES
	MLH1:NM_000249:c.1668-19A>G	rs9876116	100	Known			YES
	MLH1:NM_000249:c.1990-121C>T	rs2241031	100	Known			NO, variant in low covered regior
	MLH1:NM_000249:c.1897-167_1897-167delT	rs11290150	100		Out of pre-NGS method covered region		
T7							
	BRCA2:NM_000059:c.793+98G>A	rs206073	99,59		Out of pre-NGS method covered region		YES
	BRCA2:NM_000059:c.3807T>C	rs543304	99,78	Known			
	BRCA2:NM_000059:c.4563A>G	rs206075	100		Could not be detected by CSCE		
	BRCA2:NM_000059:c.6513G>C	rs206076	100		Could not be detected by CSCE		
	BRCA2:NM_000059:c.6938-120T>C	rs206080	100		Could not be detected by CSCE		
	BRCA2:NM_000059:c.7397T>C	rs169547	100		Could not be detected by CSCE		
	BRCA2:NM_000059:c.7617+190G>A	rs206096	100		Out of pre-NGS method covered region		
	BRCA2:NM_000059:c.9501+178A>G	rs206343	100		Out of pre-NGS method covered region		
	BRCA2:NM_000059:c.10257+105A>C	rs15869	98,06		Out of pre-NGS method covered region		
	**BRCA2:NM_000059:c.949_950insA**	**.**	**43.17**	**PAT**			NO, variant in homopolymer region
	BRCA1:NM_007294:c.5407-193A>G	rs8176310	100		Out of pre-NGS method covered region		
	BRCA1:NM_007294:c.5333-153A>G	rs8176305	46,56		Out of pre-NGS method covered region		
	BRCA1:NM_007294:c.5278-191A>T	rs8176297	98,96		Out of pre-NGS method covered region		
	BRCA1:NM_007294:c.5152+66G>A	rs3092994	100		Out of pre-NGS method covered region		
	BRCA1:NM_007294:c.5075-237C>A	rs8176257	41,18		Out of pre-NGS method covered region		
	BRCA1:NM_007294:c.5075-238A>G	rs8176256	58,82		Out of pre-NGS method covered region		
	BRCA1:NM_007294:c.5074+65G>A	rs8176235	44,33	Known			YES
	BRCA1:NM_007294:c.4987-68A>G	rs8176234	99,49		Out of pre-NGS method covered region		
	BRCA1:NM_007294:c.4987-92A>G	rs8176233	97,84		Out of pre-NGS method covered region		
	BRCA1:NM_007294:c.4986+222A>G	rs3092987	100		Out of pre-NGS method covered region		
	BRCA1:NM_007294:c.4837A>G	rs1799966	99,29	Known			YES
	BRCA1:NM_007294:c.4485-63C>G	rs8176212	99,46	Known			YES
	BRCA1:NM_007294:c.4485-137T>A	rs2236762	99,41		Out of pre-NGS method covered region		
	BRCA1:NM_007294:c.4358-2590T>G	rs8176194	98,65		Out of pre-NGS method covered region		
	BRCA1:NM_007294:c.4358-2885G>A	rs8176193	100		Out of pre-NGS method covered region		
	BRCA1:NM_007294:c.4357+117G>A	rs3737559	75,36		Out of pre-NGS method covered region		
	BRCA1:NM_007294:c.4308T>C	rs1060915	99,48	Known			YES
	BRCA1:NM_007294:c.4097-141A>C	rs799916	100		Out of pre-NGS method covered region		
	BRCA1:NM_007294:c.3548A>G	rs16942	100	Known			YES
	BRCA1:NM_007294:c.3113A>G	rs16941	99,67		Could not be detected by CSCE		
	BRCA1:NM_007294:c.2612C>T	rs799917	99,02	Known			YES
	BRCA1:NM_007294:c.2311T>C	rs16940	99,46	Known			YES
	BRCA1:NM_007294:c.2082C>T	rs1799949	98,81	Known			YES
	BRCA1:NM_007294:c.2077G>A	rs4986850	46,48	Known			YES
	BRCA1:NM_007294:c.594-225G>A	rs8176147	100		Out of pre-NGS method covered region		
	BRCA1:NM_007294:c.593+167T>C	rs8176145	100		Out of pre-NGS method covered region		
	BRCA1:NM_007294:c.547+146A>T	rs8176140	100		Out of pre-NGS method covered region		
	BRCA1:NM_007294:c.441+36C>T	rs45569832	55,56		Out of pre-NGS method covered region		
	BRCA1:NM_007294:c.213-161A>G	rs799912	100		Out of pre-NGS method covered region		
	BRCA1:NM_007294:c.1-134T>C	rs3765640	90,91		Out of pre-NGS method covered region		
	BRCA1:NM_007294:c.5277+59_5277+60insGTATTCCACTCC	.	46.04	Known			YES
	BRCA1:NM_007294:c.4485-203_4485-199delAACCC	rs34250703	70.21		Out of pre-NGS method covered region		
	BRCA1:NM_007294:c.548-58_548-58delT	rs8176144	93.54		Out of pre-NGS method covered region		
	BRCA1:NM_007294:c.441+52_441+63delCTTTTTTTTTTT	.	93.23		Out of pre-NGS method covered region		
	BRCA1:NM_007294:c.441+36_441+38delCTT	rs147856441	96.59		Out of pre-NGS method covered region		

	BRCA1:NM_007294:c.134+224_134+224delT	rs35149296	62.5		Out of pre-NGS method covered region		
T8							
	MSH6:NM_000179:c.186C>A	rs1042820	100		Could not be detected by D-HPLC		
	MSH6:NM_000179:c.260+22C>G	rs55927047	100		Could not be detected by D-HPLC		
	MSH6:NM_000179:c.276A>G	rs1800932	98,05	Known			YES
	MSH6:NM_000179:c.457+52T>A	rs3136282	98,54	Known			YES
	MSH6:NM_000179:c.457+217G>T	rs3136283	100		Out of pre-NGS method covered region		
	MSH6:NM_000179:c.458-273A>G	rs3136315	100		Out of pre-NGS method covered region		
	MSH6:NM_000179:c.458-133A>G	rs3136316	99,15	Known			NO, variant in low covered regior
	MSH6:NM_000179:c.540T>C	rs1800935	99,09	Known			YES
	MSH6:NM_000179:c.3173-101G>C	rs2072447	100	Known			YES
	MSH6:NM_000179:c.3438+14A>T	rs2020911	99,57		Could not be detected by D-HPLC		
	MSH6:NM_000179:c.3556+146G>A	rs7562048	100	Known			NO, variant in low covered regior
	MSH6:NM_000179:c.3646+91T>C	rs3136359	100	Known			YES
	MSH6:NM_000179:c.3802-40C>G	rs3136367	99,6	Known			YES
	**MSH6:NM_000179:c.3254_3254delC**	**.**	**46.39**	**PAT**			NO, variant in homopolymer region
	MSH6:NM_000179:c.3557-18_3557-17insT	.	90.24		Could not be detected by D-HPLC		
	MSH6:NM_000179:c.3646+29_3646+32delCTAT	rs2234731	88.42	Known			YES
	MSH6:NM_000179:c.4002-27_4002-27delT	.	34.74		Out of pre-NGS method covered region		
T9							
	NF1:NM_000267:c.61-300C>T	rs2269856	51,35		Out of pre-NGS method covered region		
	NF1:NM_000267:c.2034G>A	rs2285892	100	Known			YES
	NF1:NM_000267:c.3197+139T>C	rs2071009	100		Out of pre-NGS method covered region		
	NF1:NM_000267:c.3315-130G>C	rs2072131	99,75		Out of pre-NGS method covered region		
	NF1:NM_000267:c.3496+33C>A	rs2066736	100		Could not be detected by RNA-based analysis		
	NF1:NM_000267:c.3974+46T>G	rs72813695	83,3		Could not be detected by RNA-based analysis		
	**NF1:NM_000267:c.4306A>G**	**.**	**48,37**	**PAT**			YES
	NF1:NM_000267:c.4772+193T>C	rs7221954	55,43		Out of pre-NGS method covered region		
	NF1:NM_000267:c.5205+23T>C	rs9894648	48,12		Could not be detected by RNA-based analysis		
	NF1:NM_000267:c.5944-179A>C	rs10853138	100		Out of pre-NGS method covered region		
	NF1:NM_000267:c.6085-28T>A	rs7406039	50,06		Could not be detected by RNA-based analysis		
	NF1:NM_000267:c.6579+45T>A	rs17883614	48,28		Could not be detected by RNA-based analysis		
	NF1:NM_000267:c.7126+37C>G	rs7405740	100		Could not be detected by RNA-based analysis		
	NF1:NM_000267:c.8097+55T>C	rs143138479	47,06		Could not be detected by RNA-based analysis		
	NF1:NM_000267:c.2002-118_2002-117insACACACACACAC	.	33.85		Out of pre-NGS method covered region		
	NF1:NM_000267:c.3974+33_3974+34insTG	.	67.97		Could not be detected by RNA-based analysis		
	NF1:NM_000267:c.6085-30_6085-29insA	rs66638017	37.03		Could not be detected by RNA-based analysis		
T10							
	MSH6:NM_000179:c.116G>A	rs1042821	100		Region not anlyzed by pre-NGS method		
	**MSH6:NM_000179:c.255dupC**	**.**	**Not detected**	**PAT**	**Low coverage region – detected by Sanger sequencing**		NO, variant in low covered region
	MSH6:NM_000179:c.458-133A>G	rs3136316	100		Out of pre-NGS method covered region		
	MSH6:NM_000179:c.458-52G>T	rs1800934	100		Out of pre-NGS method covered region		
	MSH6:NM_000179:c.3173-101G>C	rs2072447	100		Out of pre-NGS method covered region		
	MSH6:NM_000179:c.3556+146G>A	rs7562048	100		Out of pre-NGS method covered region		
	MSH6:NM_000179:c.3646+91T>C	rs3136359	100		Out of pre-NGS method covered region		
	MSH6:NM_000179:c.3802-40C>G	rs3136367	100		Region not anlyzed by pre-NGS method		
	MSH6:NM_000179:c.3557-18_3557-17insT	.	37.5		Region not anlyzed by pre-NGS method		
	MSH6:NM_000179:c.3646+29_3646+32delCTAT	rs2234731	89.53		Region not anlyzed by pre-NGS method		
	MSH6:NM_000179:c.4002-27_4002-27delT	.	42.83		Region not anlyzed by pre-NGS method		
T11							
	NF1:NM_000267:c.61-123G>A	rs2269855	100		Out of pre-NGS method covered region		
	NF1:NM_000267:c.288+41G>A	rs2952976	100		Could not be detected by RNA-based analysis		
	NF1:NM_000267:c.480-90C>T	rs2905807	100		Out of pre-NGS method covered region		
	NF1:NM_000267:c.702G>A	rs1801052	97,47	Known			YES
	NF1:NM_000267:c.730+85A>T	.	20,57		Could not be detected by RNA-based analysis		
	NF1:NM_000267:c.888+108C>T	rs2953000	100		Out of pre-NGS method covered region		
	NF1:NM_000267:c.888+118G>T	rs2952999	100		Out of pre-NGS method covered region		
	NF1:NM_000267:c.1393-130A>T	rs2905875	100		Out of pre-NGS method covered region		

	NF1:NM_000267:c.1393-32T>C	rs2905876	100		Could not be detected by RNA-based analysis		
	NF1:NM_000267:c.1641+39T>C	rs2905880	100		Could not be detected by RNA-based analysis		
	NF1:NM_000267:c.1642-158A>G	rs17883230	70,59		Out of pre-NGS method covered region		
	NF1:NM_000267:c.2851-127G>A	rs181022226	62,86		Out of pre-NGS method covered region		
	NF1:NM_000267:c.5205+23T>C	rs9894648	51,64		Could not be detected by RNA-based analysis		
	NF1:NM_000267:c.5546+19T>A	rs2285894	47,42		Could not be detected by RNA-based analysis		
	NF1:NM_000267:c.5546+117G>A	rs3815154	41,22		Could not be detected by RNA-based analysis		
	NF1:NM_000267:c.6085-29T>A	rs7406038	42,05		Could not be detected by RNA-based analysis		
	NF1:NM_000267:c.6085-28T>A	rs7406039	47,78		Could not be detected by RNA-based analysis		
	NF1:NM_000267:c.7126+37C>G	rs7405740	50		Could not be detected by RNA-based analysis		
	NF1:NM_000267:c.7395-29G>A	rs964288	41,51		Could not be detected by RNA-based analysis		
	NF1:NM_000267:c.730+15_730+16insT	.	29.59		Could not be detected by RNA-based analysis		
	NF1:NM_000267:c.1528-37_1528-36insT	rs67472948	94.59		Could not be detected by RNA-based analysis		
	NF1:NM_000267:c.2002-118_2002-117insACAC	rs141114572	28		Out of pre-NGS method covered region		
	**NF1:NM_000267: exons 33-36 deletion**			**PAT**			YES
T12							
	NF2:NM_000268:c.1-110G>C	rs1800540	100		Out of pre-NGS method covered region		
	NF2:NM_000268:c.240+171T>A	rs2530662	100		Out of pre-NGS method covered region		
	NF2:NM_000268:c.241-58G>A	rs79901896	100		Could not be detected by RNA-based analysis		
	NF2:NM_000268:c.517-150C>T	rs6006220	46,78		Out of pre-NGS method covered region		
	NF2:NM_000268:c.600-154G>A	rs2071622	55		Out of pre-NGS method covered region		
	NF2:NM_000268:c.886-205C>T	rs2252472	45,95		Out of pre-NGS method covered region		
	NF2:NM_000268:c.1574+173G>A	rs56188151	46,15		Out of pre-NGS method covered region		
	NF2:NM_000268:c.1575-67G>A	rs140086	99,53		Could not be detected by RNA-based analysis		
	NF2:NM_000268:c.1737+1623T>C	rs7291645	60,29		Out of pre-NGS method covered region		
	NF2:NM_000268:c.599+41_599+41delT	.	28.98		Could not be detected by RNA-based analysis		
	NF2:NM_000268:c.885+173_885+173delT	rs35328234	57.89		Could not be detected by RNA-based analysis		
	NF2:NM_000268:c.1447-227_1447-226insTGAGGGA	rs3842713	31.71		Out of pre-NGS method covered region		
	**NF2:NM_000268: exons 1-5 deletion**			**PAT**			YES
T13							
	NF1:NM_000267:c.61-300C>T	rs2269856	100		Out of pre-NGS method covered region		
	NF1:NM_000267:c.61-123G>A	rs2269855	100		Out of pre-NGS method covered region		
	NF1:NM_000267:c.288+41G>A	rs2952976	100		Could not be detected by RNA-based analysis		
	NF1:NM_000267:c.480-90C>T	rs2905807	100		Out of pre-NGS method covered region		
	NF1:NM_000267:c.702G>A	rs1801052	98,48	Known			YES
	NF1:NM_000267:c.888+108C>T	rs2953000	100		Out of pre-NGS method covered region		
	NF1:NM_000267:c.888+118G>T	rs2952999	100		Out of pre-NGS method covered region		
	NF1:NM_000267:c.1393-130A>T	rs2905875	98,66		Out of pre-NGS method covered region		
	NF1:NM_000267:c.1393-32T>C	rs2905876	99,78		Could not be detected by RNA-based analysis		
	NF1:NM_000267:c.1641+39T>C	rs2905880	100		Could not be detected by RNA-based analysis		
	NF1:NM_000267:c.4368-46G>C	rs17881285	50,72		Could not be detected by RNA-based analysis		
	NF1:NM_000267:c.5205+23T>C	rs9894648	47,8		Could not be detected by RNA-based analysis		
	NF1:NM_000267:c.5546+19T>A	rs2285894	49,56		Could not be detected by RNA-based analysis		
	NF1:NM_000267:c.5546+117G>A	rs3815154	52,2		Out of pre-NGS method covered region		
	NF1:NM_000267:c.6085-29T>A	rs7406038	45,93		Could not be detected by RNA-based analysis		
	NF1:NM_000267:c.6085-28T>A	rs7406039	48,88		Could not be detected by RNA-based analysis		
	**NF1:NM_000267:c.6365-2A>G**	**.**	**45,71**	**PAT**			YES
	NF1:NM_000267:c.7126+37C>G	rs7405740	43,85		Could not be detected by RNA-based analysis		
	NF1:NM_000267:c.7395-29G>A	rs964288	55,22		Could not be detected by RNA-based analysis		
	NF1:NM_000267:c.7907+166C>T	rs7350943	45,45		Out of pre-NGS method covered region		
	NF1:NM_000267:c.8097+30G>T	.	27,54		Could not be detected by RNA-based analysis		
	NF1:NM_000267:c.8097+71A>T	.	30,61		Could not be detected by RNA-based analysis		
	NF1:NM_000267:c.8097+76C>A	.	26,19		Could not be detected by RNA-based analysis		
	NF1:NM_000267:c.730+15_730+16insT	.	27.83		Could not be detected by RNA-based analysis		
	NF1:NM_000267:c.889-191_889-190insT	rs35966725	100		Out of pre-NGS method covered region		
	NF1:NM_000267:c.1528-37_1528-36insT	rs67472948	94.58		Could not be detected by RNA-based analysis		
	NF1:NM_000267:c.2002-118_2002-117insAC	rs141114572	62.39		Out of pre-NGS method covered region		
T14							

	**APC:NM_001127510:c.423-3T>A**	**.**	**44,24**	**PAT**			YES
	APC:NM_001127510:c.645+173A>G	rs2289484	48,48	Known			YES
	APC:NM_001127510:c.1458T>C	rs2229992	49,55	Known			YES
	APC:NM_001127510:c.1635G>A	rs351771	46,6	Known			YES
	APC:NM_001127510:c.1743+193G>A	rs351772	47,62		Out of pre-NGS method covered region		
	APC:NM_001127510:c.4479G>A	rs41115	42,42	Known			YES
	APC:NM_001127510:c.5034G>A	rs42427	55,11	Known			YES
	APC:NM_001127510:c.5268T>G	rs866006	50,91	Known			YES
	APC:NM_001127510:c.5465T>A	rs459552	100	Known			YES
	APC:NM_001127510:c.5880G>A	rs465899	52,59	Known			YES
	APC:NM_001127510:c.934-120_934-119insG	rs138003874	26.76		Out of pre-NGS method covered region		
	APC:NM_001127510:c.1959-145_1959-144insAGAA	rs3839284	47.69		Out of pre-NGS method covered region		
T15							
	MSH2:NM_000251:c.943-161C>T	rs17224276	48,65		Out of pre-NGS method covered region		
	MSH2:NM_000251:c.1077-80G>A	rs2347794	57,6		Out of pre-NGS method covered region		
	MSH2:NM_000251:c.1277-118G>A	rs1981929	50,29		Out of pre-NGS method covered region		
	MSH2:NM_000251:c.1661+90T>C	rs10183143	49,43		Out of pre-NGS method covered region		
	MSH2:NM_000251:c.2006-127C>T	rs2059521	100		Out of pre-NGS method covered region		
	MSH2:NM_000251:c.2210+175G>A	rs4583514	32,14		Out of pre-NGS method covered region		
	MSH2:NM_000251:c.2635-111T>A	rs6737098	53,63		Out of pre-NGS method covered region		
	**MSH2:NM_000251:c.597_598insT**	**.**	**47.18**	**PAT**			NO, variant in homopolymer region
	MSH2:NM_000251:c.1276+117_1276+117delT	.	22.41		Out of pre-NGS method covered region		
T16							
	BRCA2:NM_000059:c.1-26G>A	rs1799943	49,36		Out of pre-NGS method covered region		
	BRCA2:NM_000059:c.425+246G>C	rs11571613	100		Out of pre-NGS method covered region		
	BRCA2:NM_000059:c.631+183T>A	rs3752451	100		Out of pre-NGS method covered region		
	BRCA2:NM_000059:c.793+98G>A	rs206073	99,29		Out of pre-NGS method covered region		
	BRCA2:NM_000059:c.3396A>G	rs1801406	99,68		Could not be detected by CSCE		
	BRCA2:NM_000059:c.4563A>G	rs206075	99,77		Could not be detected by CSCE		
	BRCA2:NM_000059:c.6513G>C	rs206076	100		Could not be detected by CSCE		
	BRCA2:NM_000059:c.6841+80T>G	.	77,78		Out of pre-NGS method covered region		
	BRCA2:NM_000059:c.6841+191C>A	rs11571662	100		Out of pre-NGS method covered region		
	BRCA2:NM_000059:c.6938-120T>C	rs206080	99,6		Out of pre-NGS method covered region		
	BRCA2:NM_000059:c.7242A>G	rs1799955	49,46		Region not anlyzed by pre-NGS method		
	BRCA2:NM_000059:c.7397T>C	rs169547	100		Could not be detected by CSCE		
	BRCA2:NM_000059:c.7617+190G>A	rs206096	100		Out of pre-NGS method covered region		
	BRCA2:NM_000059:c.7806-14T>C	rs9534262	48,3		Region not anlyzed by pre-NGS method		
	BRCA2:NM_000059:c.6841+78_6841+81delAATT	rs138193280	87.92		Out of pre-NGS method covered region		
	**BRCA2:NM_000059:c.8942_8942delA**	**.**	**50.6**	**PAT**			NO, variant in homopolymer region
T17							
	BRCA2:NM_000059:c.793+98G>A	rs206073	100		Could not be detected by CSCE		
	BRCA2:NM_000059:c.1114A>C	rs144848	100	Known			YES
	BRCA2:NM_000059:c.4563A>G	rs206075	99,66		Could not be detected by CSCE		
	BRCA2:NM_000059:c.6513G>C	rs206076	99,74		Could not be detected by CSCE		
	BRCA2:NM_000059:c.6938-120T>C	rs206080	99,07		Could not be detected by CSCE		
	BRCA2:NM_000059:c.7397T>C	rs169547	100		Could not be detected by CSCE		
	BRCA2:NM_000059:c.7617+190G>A	rs206096	100		Could not be detected by CSCE		
	BRCA2:NM_000059:c.9648+244T>C	rs206344	100		Could not be detected by CSCE		
	**BRCA2:NM_000059:c.2402_2420delACAATTATGAATCTGATGT**	**.**	**38,89**	**PAT**			YES
	BRCA1:NM_007294:c.5407-193A>G	rs8176310	44,23		Out of pre-NGS method covered region		
	BRCA1:NM_007294:c.5333-153A>G	rs8176305	49,38		Out of pre-NGS method covered region		
	BRCA1:NM_007294:c.5278-191A>T	rs8176297	52,89		Out of pre-NGS method covered region		
	BRCA1:NM_007294:c.5152+66G>A	rs3092994	44,67		Out of pre-NGS method covered region		
	BRCA1:NM_007294:c.5075-53C>T	rs8176258	45,16	Known			YES
	BRCA1:NM_007294:c.5075-238A>G	rs8176256	45,83		Out of pre-NGS method covered region		
	BRCA1:NM_007294:c.4987-68A>G	rs8176234	51,65		Out of pre-NGS method covered region		
	BRCA1:NM_007294:c.4987-92A>G	rs8176233	47,72		Out of pre-NGS method covered region		
	BRCA1:NM_007294:c.4986+222A>G	rs3092987	73,68		Out of pre-NGS method covered region		

	BRCA1:NM_007294:c.4837A>G	rs1799966	48,05	Known			
	BRCA1:NM_007294:c.4485-63C>G	rs8176212	46,37	Known			YES
	BRCA1:NM_007294:c.4485-137T>A	rs2236762	45,12		Out of pre-NGS method covered region		
	BRCA1:NM_007294:c.4358-2590T>G	rs8176194	52,1		Out of pre-NGS method covered region		
	BRCA1:NM_007294:c.4358-2885G>A	rs8176193	50,44		Out of pre-NGS method covered region		
	BRCA1:NM_007294:c.4308T>C	rs1060915	43,87	Known			YES
	BRCA1:NM_007294:c.4097-141A>C	rs799916	54,68		Out of pre-NGS method covered region		
	BRCA1:NM_007294:c.3548A>G	rs16942	46,11	Known			YES
	BRCA1:NM_007294:c.3113A>G	rs16941	47,65	Known			YES
	BRCA1:NM_007294:c.2612C>T	rs799917	52,96	Known			YES
	BRCA1:NM_007294:c.2311T>C	rs16940	49,12	Known			YES
	BRCA1:NM_007294:c.2082C>T	rs1799949	45,98	Known			YES
	BRCA1:NM_007294:c.2077G>A	rs4986850	45,48	Known			YES
	BRCA1:NM_007294:c.594-225G>A	rs8176147	44,74		Out of pre-NGS method covered region		
	BRCA1:NM_007294:c.593+167T>C	rs8176145	39,47	Known			YES
	BRCA1:NM_007294:c.547+146A>T	rs8176140	53,42		Out of pre-NGS method covered region		
	BRCA1:NM_007294:c.213-161A>G	rs799912	46,39		Out of pre-NGS method covered region		
	BRCA1:NM_007294:c.1-134T>C	rs3765640	35,29	Known			YES
	BRCA1:NM_007294:c.4485-203_4485-199delAACCC	rs34250703	40.58		Out of pre-NGS method covered region		
	BRCA1:NM_007294:c.548-58_548-58delT	rs8176144	49.24		Out of pre-NGS method covered region		
	BRCA1:NM_007294:c.441+52_441+63delCTTTTTTTTTTT	.	52.52		Out of pre-NGS method covered region		
	BRCA1:NM_007294:c.441+36_441+38delCTT	rs147856441	44.87		Out of pre-NGS method covered region		
	BRCA1:NM_007294:c.134+224_134+224delT	rs35149296	60		Out of pre-NGS method covered region		
T18							
	BRCA2:NM_000059:c.1-26G>A	rs1799943	45,98	Known			YES
	BRCA2:NM_000059:c.425+246G>C	rs11571613	50		Out of pre-NGS method covered region		
	BRCA2:NM_000059:c.631+183T>A	rs3752451	34,21		Out of pre-NGS method covered region		
	BRCA2:NM_000059:c.793+98G>A	rs206073	99,41		Out of pre-NGS method covered region		
	BRCA2:NM_000059:c.1114A>C	rs144848	51,24	Known			YES
	BRCA2:NM_000059:c.3396A>G	rs1801406	45,41	Known			YES
	BRCA2:NM_000059:c.4563A>G	rs206075	100		Could not be detected by CSCE		
	BRCA2:NM_000059:c.6513G>C	rs206076	100		Could not be detected by CSCE		
	BRCA2:NM_000059:c.6841+191C>A	rs11571662	52,69		Out of pre-NGS method covered region		
	BRCA2:NM_000059:c.6937+239A>T	rs190434310	61,54		Out of pre-NGS method covered region		
	BRCA2:NM_000059:c.6938-120T>C	rs206080	98,45		Out of pre-NGS method covered region		
	BRCA2:NM_000059:c.7242A>G	rs1799955	47,78	Known			YES
	BRCA2:NM_000059:c.7397T>C	rs169547	100		Could not be detected by CSCE		
	BRCA2:NM_000059:c.7617+190G>A	rs206096	100		Out of pre-NGS method covered region		
	BRCA2:NM_000059:c.7806-14T>C	rs9534262	49,88	Known			YES
	BRCA2:NM_000059:c.8754+183A>C	rs3764791	39,13		Out of pre-NGS method covered region		
	BRCA2:NM_000059:c.8754+187C>T	rs3764792	39,02		Out of pre-NGS method covered region		
	BRCA2:NM_000059:c.8755-66T>C	rs4942486	45,41		Out of pre-NGS method covered region		
	BRCA2:NM_000059:c.9257-219A>G	rs7330025	70		Out of pre-NGS method covered region		
	BRCA2:NM_000059:c.9257-16T>C	rs11571818	45,36	Known			YES
	BRCA2:NM_000059:c.9648+244T>C	rs206344	100		Out of pre-NGS method covered region		
	BRCA2:NM_000059:c.9976A>T	rs11571833	49,17	Known			YES
	**BRCA2:NM_000059:c.6275_6276delTT**	**rs11571658**	**43.6**	**PAT**			YES
	BRCA2:NM_000059:c.6841+78_6841+81delAATT	rs138193280	47.75		Out of pre-NGS method covered region		
	BRCA2:NM_000059:c.8633-150_8633-149insA	rs200156944	44.83		Out of pre-NGS method covered region		
T19							
	MSH6:NM_000179:c.116G>A	rs1042821	52,11	Known			NO, variant in low covered regior
	MSH6:NM_000179:c.458-133A>G	rs3136316	50	Known			YES
	MSH6:NM_000179:c.458-52G>T	rs1800934	49,64	Known			YES
	MSH6:NM_000179:c.2633T>C	rs2020912	49,86	Known			YES
	**MSH6:NM_000179:c.3148_3149insTGTAGAGTGTAT**	**.**	**33.72**	**PAT**			YES
	MSH6:NM_000179:c.4002-27_4002-27delT	.	36.45		Out of pre-NGS method covered region		
T20							
	MSH6:NM_000179:c.186C>A	rs1042820	56,44	Known			NO, variant in low covered regior

	MSH6:NM_000179:c.260+22C>G	rs55927047	44,44	Known			NO, variant in low covered regior
	MSH6:NM_000179:c.276A>G	rs1800932	49,34	Known			YES
	MSH6:NM_000179:c.457+52T>A	rs3136282	52,34	Known			YES
	MSH6:NM_000179:c.458-133A>G	rs3136316	56,31	Known			YES
	MSH6:NM_000179:c.540T>C	rs1800935	44,8		Region not anlyzed by pre-NGS method		
	MSH6:NM_000179:c.3173-101G>C	rs2072447	55,2		Out of pre-NGS method covered region		
	MSH6:NM_000179:c.3438+14A>T	rs2020911	53,03		Region not anlyzed by pre-NGS method		
	MSH6:NM_000179:c.3556+146G>A	rs7562048	56,45	Known			YES
	MSH6:NM_000179:c.3646+91T>C	rs3136359	46,43		Out of pre-NGS method covered region		
	MSH6:NM_000179:c.3802-40C>G	rs3136367	47,03		Out of pre-NGS method covered region		
	**MSH6:NM_000179:c.3252_3253insC**	**.**	**41.13**	**PAT**			NO, variant in homopolymer region
	MSH6:NM_000179:c.3557-18_3557-17insT	.	43.88		Region not anlyzed by pre-NGS method		
	MSH6:NM_000179:c.3646+29_3646+32delCTAT	rs2234731	33.04	Known			YES
	MSH6:NM_000179:c.4002-27_4002-27delT	.	35.14		Region not anlyzed by pre-NGS method		
T21							
	MLH1:NM_000249:c.453+79A>G	rs4234259	99,12		Out of pre-NGS method covered region		
	MLH1:NM_000249:c.655A>G	rs1799977	99,59		Region not anlyzed by pre-NGS method		
	MLH1:NM_000249:c.791-79A>G	rs104894998	51,84		Out of pre-NGS method covered region		
	MLH1:NM_000249:c.1038+86T>C	rs2286939	100		Out of pre-NGS method covered region		
	MLH1:NM_000249:c.1039-78A>G	rs11129748	92,31		Out of pre-NGS method covered region		
	MLH1:NM_000249:c.1410-169C>T	rs2286940	100		Out of pre-NGS method covered region		
	MLH1:NM_000249:c.1668-19A>G	rs9876116	100		Region not anlyzed by pre-NGS method		
	MLH1:NM_000249:c.1990-121C>T	rs2241031	100		Out of pre-NGS method covered region		
	MLH1:NM_000249:c.208-150_208-150delA	rs111908792	21.62		Out of pre-NGS method covered region		
	MLH1:NM_000249:c.678-212_678-211insGGGA	rs138898092	70		Out of pre-NGS method covered region		
	MLH1:NM_000249:c.1039-28_1039-26delTTT	.	37.5		Region not anlyzed by pre-NGS method		
	**MLH1:NM_000249:c.1588_1589insCGTGGGCTG**	**.**	**39.49**	**PAT**			YES
	MLH1:NM_000249:c.1897-167_1897-167delT	rs11290150	100		Out of pre-NGS method covered region		
T22							
	NF1:NM_000267:c.61-300C>T	rs2269856	100		Out of pre-NGS method covered region		
	NF1:NM_000267:c.61-123G>A	rs2269855	99,36		Out of pre-NGS method covered region		
	NF1:NM_000267:c.288+41G>A	rs2952976	100		Could not be detected by RNA-based analysis		
	NF1:NM_000267:c.480-90C>T	rs2905807	100		Out of pre-NGS method covered region		
	NF1:NM_000267:c.702G>A	rs1801052	98,56	Known			YES
	NF1:NM_000267:c.888+108C>T	rs2953000	100		Out of pre-NGS method covered region		
	NF1:NM_000267:c.888+118G>T	rs2952999	100		Out of pre-NGS method covered region		
	NF1:NM_000267:c.1393-130A>T	rs2905875	100		Out of pre-NGS method covered region		
	NF1:NM_000267:c.1393-32T>C	rs2905876	99,83		Could not be detected by RNA-based analysis		
	NF1:NM_000267:c.1641+39T>C	rs2905880	100		Could not be detected by RNA-based analysis		
	NF1:NM_000267:c.4368-46G>C	rs17881285	43,74		Could not be detected by RNA-based analysis		
	NF1:NM_000267:c.5205+23T>C	rs9894648	100		Could not be detected by RNA-based analysis		
	NF1:NM_000267:c.5546+19T>A	rs2285894	99,19		Could not be detected by RNA-based analysis		
	NF1:NM_000267:c.5546+117G>A	rs3815154	100		Out of pre-NGS method covered region		
	NF1:NM_000267:c.6085-29T>A	rs7406038	88,82		Could not be detected by RNA-based analysis		
	NF1:NM_000267:c.6085-28T>A	rs7406039	97,6		Could not be detected by RNA-based analysis		
	**NF1:NM_000267:c.6226delG**		**52,4**	**PAT**			YES
	NF1:NM_000267:c.7126+37C>G	rs7405740	100		Could not be detected by RNA-based analysis		
	NF1:NM_000267:c.7127-167T>A	rs2525569	100		Out of pre-NGS method covered region		
	NF1:NM_000267:c.7395-29G>A	rs964288	100		Could not be detected by RNA-based analysis		
	NF1:NM_000267:c.7907+166C>T	rs7350943	100		Out of pre-NGS method covered region		
	NF1:NM_000267:c.8050+20G>A	rs55747230	49,26		Could not be detected by RNA-based analysis		
T23							
	BRCA2:NM_000059:c.1-26G>A	rs1799943	45,37	Known			YES
	BRCA2:NM_000059:c.425+67A>C	rs11571610	51,53	Known			YES
	BRCA2:NM_000059:c.426-89T>C	rs3783265	52,3	Known			YES
	BRCA2:NM_000059:c.631+183T>A	rs3752451	100		Out of pre-NGS method covered region		
	BRCA2:NM_000059:c.793+98G>A	rs206073	99,29		Out of pre-NGS method covered region		
	BRCA2:NM_000059:c.865A>C	rs766173	53,76	Known			YES

	BRCA2:NM_000059:c.1365A>G	rs1801439	43,9	Known			YES
	BRCA2:NM_000059:c.1910-51G>T	rs11571651	48,68		Out of pre-NGS method covered region		
	BRCA2:NM_000059:c.2229T>C	rs1801499	51,14	Known			YES
	BRCA2:NM_000059:c.2971A>G	rs1799944	50,49	Known			YES
	BRCA2:NM_000059:c.3396A>G	rs1801406	50,24	Known			YES
	BRCA2:NM_000059:c.4563A>G	rs206075	99,77		Could not be detected by CSCE		
	BRCA2:NM_000059:c.6513G>C	rs206076	100		Could not be detected by CSCE		
	BRCA2:NM_000059:c.6841+191C>A	rs11571662	45,3		Out of pre-NGS method covered region		
	BRCA2:NM_000059:c.6938-120T>C	rs206080	99,15		Out of pre-NGS method covered region		
	BRCA2:NM_000059:c.7397T>C	rs169547	100		Could not be detected by CSCE		
	BRCA2:NM_000059:c.7435+53C>T	rs11147489	48,33	Known			YES
	BRCA2:NM_000059:c.7617+190G>A	rs206096	100		Out of pre-NGS method covered region		
	BRCA2:NM_000059:c.7806-14T>C	rs9534262	49,39	Known			YES
	BRCA2:NM_000059:c.8755-66T>C	rs4942486	46,5		Out of pre-NGS method covered region		
	BRCA2:NM_000059:c.8953+98T>C	rs81002901	51,75		Out of pre-NGS method covered region		
	BRCA2:NM_000059:c.9257-219A>G	rs7330025	60		Out of pre-NGS method covered region		
	BRCA2:NM_000059:c.6841+78_6841+81delAATT	rs138193280	41.81		Out of pre-NGS method covered region		
	BRCA1:NM_007300:c.5050-142_5050-142delA	.	24		Out of pre-NGS method covered region		
	**BRCA1:NM_007300:c.1953_1956delGAAA**	**rs80357526**	**46.81**	**PAT**			YES
VALIDATION							
V1							
	MUTYH:NM_012222:c.1477-40C>G	rs3219493	100	Known			
	MUTYH:NM_012222:c.1477-125C>A	rs3219492	37,74		Out of pre-NGS method covered region		
	**MUTYH:NM_012222:c.1187G>A**	**rs36053993**	**51,76**	**PAT**			
	MUTYH:NM_012222:c.1014G>C	rs3219489	57,85	Known			
	MUTYH:NM_012222:c.504+35A>G	rs3219487	100		Out of pre-NGS method covered region		
	**MUTYH:NM_012222:c.481G>C**	**.**	**51,04**	**VUS**			
	APC:NM_001127511:c.1-133C>G	rs79896135	43,64		Out of pre-NGS method covered region		
	APC:NM_001127511:c.1-31T>G	rs78429131	50		Out of pre-NGS method covered region		
	APC:NM_001127510:c.645+32C>T	rs2909961	55,04		Out of pre-NGS method covered region		
	APC:NM_001127510:c.645+173A>G	rs2289484	50		Out of pre-NGS method covered region		
	APC:NM_001127510:c.934-132G>T	rs12521276	100		Out of pre-NGS method covered region		
	APC:NM_001127510:c.1458T>C	rs2229992	99,51	Known			
	APC:NM_001127510:c.1635G>A	rs351771	50,97	Known			
	APC:NM_001127510:c.1743+193G>A	rs351772	91,67		Out of pre-NGS method covered region		
	APC:NM_001127510:c.1744-158A>G	rs74915204	47,06		Out of pre-NGS method covered region		
	APC:NM_001127510:c.4479G>A	rs41115	48,48	Known			
	APC:NM_001127510:c.5034G>A	rs42427	57,46	Known			
	APC:NM_001127510:c.5268T>G	rs866006	43,49	Known			
	APC:NM_001127510:c.5465T>A	rs459552	99,61	Known			
	APC:NM_001127510:c.5880G>A	rs465899	49,35	Known			
	APC:NM_001127510:c.7201C>T	rs2229994	49,49	Known			
	APC:NM_001127510:c.934-132_934-132delG	rs141013063	45.9		Out of pre-NGS method covered region		
	APC:NM_001127510:c.934-120_934-119insG	rs138003874	24.71		Out of pre-NGS method covered region		
	APC:NM_001127510:c.1959-145_1959-144insAGAA	rs3839284	100		Out of pre-NGS method covered region		
V2							
	APC:NM_001127510:c.645+173A>G	rs2289484	65,52	Known			
	APC:NM_001127510:c.934-132G>T	rs12521276	28,57		Out of pre-NGS method covered region		
	APC:NM_001127510:c.1458T>C	rs2229992	46,25	Known			
	**APC:NM_001127510:c.1548+1G>C**	**.**	**47,74**	**PAT**			
	APC:NM_001127510:c.1635G>A	rs351771	52,91	Known			
	APC:NM_001127510:c.1743+193G>A	rs351772	Not detected	Known	Out of I2CHP ROI		
	APC:NM_001127510:c.1744-37A>G	.	51,29	Known			
	APC:NM_001127510:c.4479G>A	rs41115	51,63	Known			
	APC:NM_001127510:c.5034G>A	rs42427	49,54	Known			
	APC:NM_001127510:c.5268T>G	rs866006	49,72	Known			
	APC:NM_001127510:c.5465T>A	rs459552	49,39	Known			
	APC:NM_001127510:c.5880G>A	rs465899	55,8	Known			

	APC:NM_001127510:c.934-120_934-119insG	rs138003874	28		Out of pre-NGS method covered region		
	APC:NM_001127510:c.1959-145_1959-144insAGAA	rs3839284	48.24		Out of pre-NGS method covered region		
V3							
	MSH2:NM_000251:c.1-118T>C	rs2303425	58,99		Out of pre-NGS method covered region		
	MSH2:NM_000251:c.211+9C>G	rs2303426	98,51	Known			
	MSH2:NM_000251:c.211+98T>C	rs3815865	92,86		Out of pre-NGS method covered region		
	MSH2:NM_000251:c.367-168C>T	rs17217758	60,38		Out of pre-NGS method covered region		
	**MSH2:NM_000251:c.518T>G**	**rs63750070**	**44,49**	**VUS**			
	MSH2:NM_000251:c.646-224A>G	rs17217786	54,55		Out of pre-NGS method covered region		
	MSH2:NM_000251:c.1077-80G>A	rs2347794	100		Out of pre-NGS method covered region		
	MSH2:NM_000251:c.1077-10T>C	rs17224360	54,4	Known			
	MSH2:NM_000251:c.1277-212T>A	rs1981928	100		Out of pre-NGS method covered region		
	MSH2:NM_000251:c.1277-118G>A	rs1981929	100		Out of pre-NGS method covered region		
	MSH2:NM_000251:c.1510+111T>C	rs3771278	99,23		Out of pre-NGS method covered region		
	MSH2:NM_000251:c.1510+115A>G	rs3771279	100		Out of pre-NGS method covered region		
	MSH2:NM_000251:c.1510+118T>C	rs3771280	100		Out of pre-NGS method covered region		
	MSH2:NM_000251:c.1511-91G>T	rs3732182	100	Known			
	MSH2:NM_000251:c.1511-9A>T	rs12998837	57,84	Known			
	MSH2:NM_000251:c.1661+12G>A	rs3732183	100	Known			
	MSH2:NM_000251:c.1759+107A>G	rs3764959	100	Known			
	MSH2:NM_000251:c.1759+183G>A	rs3764960	98,31	Known			
	MSH2:NM_000251:c.2006-127C>T	rs2059521	100		Out of pre-NGS method covered region		
	MSH2:NM_000251:c.2006-6T>C	rs2303428	48,79	Known			
	MSH2:NM_000251:c.2210+175G>A	rs4583514	100		Out of pre-NGS method covered region		
	MSH2:NM_000251:c.2635-214T>C	rs2042649	59,46		Out of pre-NGS method covered region		
	MSH2:NM_000251:c.792+148_792+148delT	.	27.78		Out of pre-NGS method covered region		
V4							
	BRCA2:NM_000059:c.1-26G>A	rs1799943	43,6	Known			
	BRCA2:NM_000059:c.631+183T>A	rs3752451	85,19		Out of pre-NGS method covered region		
	BRCA2:NM_000059:c.793+98G>A	rs206073	99,05		Out of pre-NGS method covered region		
	BRCA2:NM_000059:c.1114A>C	rs144848	51,94	Known			
	BRCA2:NM_000059:c.2803G>A	rs28897716	40,53	Known			
	BRCA2:NM_000059:c.3396A>G	rs1801406	49,7	Known			
	BRCA2:NM_000059:c.4563A>G	rs206075	99,73		Region not anlyzed by pre-NGS method		
	**BRCA2:NM_000059:c.5351A>T**	**.**	**47,55**	**VUS**	Region not anlyzed by pre-NGS method		
	BRCA2:NM_000059:c.6513G>C	rs206076	99,81		Region not anlyzed by pre-NGS method		
	BRCA2:NM_000059:c.6841+191C>A	rs11571662	51,2		Out of pre-NGS method covered region		
	BRCA2:NM_000059:c.6938-120T>C	rs206080	100		Out of pre-NGS method covered region		
	BRCA2:NM_000059:c.7008-62A>G	rs76584943	49,33	Known			
	BRCA2:NM_000059:c.7242A>G	rs1799955	47,43	Known			
	BRCA2:NM_000059:c.7397T>C	rs169547	99,62		Region not anlyzed by pre-NGS method		
	BRCA2:NM_000059:c.7617+190G>A	rs206096	100		Out of pre-NGS method covered region		
	BRCA2:NM_000059:c.7806-14T>C	rs9534262	47,76	Known			
	BRCA2:NM_000059:c.8754+183A>C	rs3764791	40		Out of pre-NGS method covered region		
	BRCA2:NM_000059:c.8754+187C>T	rs3764792	43,33		Out of pre-NGS method covered region		
	BRCA2:NM_000059:c.8755-66T>C	rs4942486	52,36		Out of pre-NGS method covered region		
	**BRCA2:NM_000059:c.5345_5345delA**	**.**	**44.09**	**PAT**			
	BRCA2:NM_000059:c.6841+78_6841+81delAATT	rs138193280	36.03		Out of pre-NGS method covered region		
	BRCA1:NM_007294:c.5407-193A>G	rs8176310	55		Out of pre-NGS method covered region		
	BRCA1:NM_007294:c.4485-137T>A	rs2236762	49,44		Out of pre-NGS method covered region		
	BRCA1:NM_007294:c.4097-141A>C	rs799916	50,73		Out of pre-NGS method covered region		
	BRCA1:NM_007294:c.2612C>T	rs799917	47,32		Out of pre-NGS method covered region		
	BRCA1:NM_007294:c.213-161A>G	rs799912	59,09		Out of pre-NGS method covered region		
	BRCA1:NM_007294:c.441+64_441+64delT	rs368252296	53,33		Out of pre-NGS method covered region		
V5							
	MLH1:NM_000249:c.453+79A>G	rs4234259	100		Out of pre-NGS method covered region		
	MLH1:NM_000249:c.655A>G	rs1799977	55,38		Region not anlyzed by pre-NGS method		
	MLH1:NM_000249:c.1038+86T>C	rs2286939	100		Out of pre-NGS method covered region		

	MLH1:NM_000249:c.1410-169C>T	rs2286940	100		Out of pre-NGS method covered region		
	MLH1:NM_000249:c.1668-19A>G	rs9876116	100		Region not anlyzed by pre-NGS method		
	MLH1:NM_000249:c.1990-121C>T	rs2241031	97,67		Out of pre-NGS method covered region		
	MLH1:NM_000249:c.1897-167_1897-167delT	rs11290150	100		Out of pre-NGS method covered region		
	**MLH1:NM_000249:c.2148_2149insAACA**	**.**	**48.71**	**PAT**			
V6							
	PMS2:NM_000535:c.2570G>C	rs1802683	42,07		Region not anlyzed by pre-NGS method		
	PMS2:NM_000535:c.2446-118G>A	0	21,88		Out of pre-NGS method covered region		
	PMS2:NM_000535:c.2276-135T>C	rs9655490	41,42		Out of pre-NGS method covered region		
	PMS2:NM_000535:c.2007-4G>A	rs1805326	27,91		Region not anlyzed by pre-NGS method		
	PMS2:NM_000535:c.1621A>G	rs2228006	99,67		Region not anlyzed by pre-NGS method		
	PMS2:NM_000535:c.1408C>T	rs1805321	45,04		Region not anlyzed by pre-NGS method		
	PMS2:NM_000535:c.1145-57G>A	rs199550985	20,62		Out of pre-NGS method covered region		
	**PMS2:NM_000535:c.943C>T**	**rs200640585**	**36,43**	**PAT**			
	PMS2:NM_000535:c.804-164T>G	rs2286681	22,41		Region not anlyzed by pre-NGS method		
	PMS2:NM_000535:c.780C>G	rs1805319	99,66		Region not anlyzed by pre-NGS method		
	PMS2:NM_000535:c.705+17A>G	rs62456182	43,37		Region not anlyzed by pre-NGS method		
	PMS2:NM_000535:c.251-72A>G	rs117831773	44,49		Out of pre-NGS method covered region		
	PMS2:NM_000535:c.904-88_904-86delCTG	rs200573843	56		Out of pre-NGS method covered region		
	PMS2:NM_000535:c.705+118_705+118delT	rs11289444	83.33		Out of pre-NGS method covered region		
V7							
	APC:NM_001127511:c.1-133C>G	rs79896135	38,78		Out of pre-NGS method covered region		
	APC:NM_001127511:c.1-31T>G	rs78429131	32,12		Region not anlyzed by pre-NGS method		
	APC:NM_001127510:c.645+32C>T	rs2909961	48,44		Region not anlyzed by pre-NGS method		
	APC:NM_001127510:c.645+173A>G	rs2289484	60,71	Known			
	APC:NM_001127510:c.934-132G>T	rs12521276	100	Known			
	**APC:NM_001127510:c.1312+3A>G**	**.**	**53,08**	**PAT**			
	APC:NM_001127510:c.1458T>C	rs2229992	98,97	Known			
	APC:NM_001127510:c.1635G>A	rs351771	49,49	Known			
	APC:NM_001127510:c.1743+193G>A	rs351772	Not detected	Known	Out of I2CHP ROI		
	APC:NM_001127510:c.1744-158A>G	rs74915204	50		Out of pre-NGS method covered region		
	APC:NM_001127510:c.4479G>A	rs41115	48,71		Region not anlyzed by pre-NGS method		
	APC:NM_001127510:c.5034G>A	rs42427	53,38		Region not anlyzed by pre-NGS method		
	APC:NM_001127510:c.5268T>G	rs866006	48,41		Region not anlyzed by pre-NGS method		
	APC:NM_001127510:c.5465T>A	rs459552	100		Region not anlyzed by pre-NGS method		
	APC:NM_001127510:c.5880G>A	rs465899	53,66		Region not anlyzed by pre-NGS method		
	APC:NM_001127510:c.7201C>T	rs2229994	48,04		Region not anlyzed by pre-NGS method		
	APC:NM_001127510:c.934-132_934-132delG	rs141013063	68.42		Out of pre-NGS method covered region		
	APC:NM_001127510:c.934-120_934-119insG	rs138003874	28.57		Out of pre-NGS method covered region		
	APC:NM_001127510:c.1959-145_1959-144insAGAA	rs3839284	97.87		Out of pre-NGS method covered region		
V8							
	BRCA2:NM_000059:c.793+98G>A	rs206073	99,18		Out of pre-NGS method covered region		
	BRCA2:NM_000059:c.1114A>C	rs144848	99,85	Known			
	BRCA2:NM_000059:c.4563A>G	rs206075	99,75	Known			
	BRCA2:NM_000059:c.5199C>T	rs28897734	48,22	Known			
	BRCA2:NM_000059:c.6513G>C	rs206076	100	Known			
	BRCA2:NM_000059:c.6841+191C>A	rs11571662	34,78		Out of pre-NGS method covered region		
	BRCA2:NM_000059:c.6938-120T>C	rs206080	98,48		Out of pre-NGS method covered region		
	BRCA2:NM_000059:c.7242A>G	rs1799955	50	Known			
	BRCA2:NM_000059:c.7397T>C	rs169547	100	Known			
	BRCA2:NM_000059:c.7617+190G>A	rs206096	100		Out of pre-NGS method covered region		
	BRCA2:NM_000059:c.7806-14T>C	rs9534262	54,05	Known			
	BRCA2:NM_000059:c.8754+183A>C	rs3764791	50		Out of pre-NGS method covered region		
	BRCA2:NM_000059:c.8754+187C>T	rs3764792	45,16		Out of pre-NGS method covered region		
	BRCA2:NM_000059:c.8755-66T>C	rs4942486	43,66		Out of pre-NGS method covered region		
	BRCA2:NM_000059:c.6841+78_6841+81delAATT	rs138193280	38,77		Out of pre-NGS method covered region		
	TP53:NM_001126112:c.1101-221G>A	rs6503048	39,58		Out of pre-NGS method covered region		
	**TP53:NM_001126112:c.743G>A**	**rs11540652**	**43,57**	**PAT**			

	TP53:NM_001126112:c.672+62A>G	rs1625895	46,98		Out of pre-NGS method covered region		
	TP53:NM_001126112:c.376-91G>A	rs2909430	57,98		Out of pre-NGS method covered region		
	TP53:NM_001126112:c.376-101A>T	rs145153611	54,63		Out of pre-NGS method covered region		
	TP53:NM_001126112:c.97-52G>A	.	56,65		Out of pre-NGS method covered region		
	TP53:NM_001126112:c.672+194_672+194delC	.	58.82		Out of pre-NGS method covered region		
	TP53:NM_001126112:c.96+41_97-54delACCTGGAGGGCTGGGG	rs146534833	44.64		Out of pre-NGS method covered region		
V9							
	MSH6:NM_000179:c.276A>G	rs1800932	48,68	Known			
	MSH6:NM_000179:c.457+52T>A	rs3136282	52,54		Out of pre-NGS method covered region		
	MSH6:NM_000179:c.458-133A>G	rs3136316	97,56		Out of pre-NGS method covered region		
	MSH6:NM_000179:c.540T>C	rs1800935	46,62	Known			
	MSH6:NM_000179:c.3173-101G>C	rs2072447	72,5		Out of pre-NGS method covered region		
	MSH6:NM_000179:c.3438+14A>T	rs2020911	61,97		Out of pre-NGS method covered region		
	MSH6:NM_000179:c.3556+146G>A	rs7562048	35,29		Out of pre-NGS method covered region		
	MSH6:NM_000179:c.3646+91T>C	rs3136359	56,25		Out of pre-NGS method covered region		
	MSH6:NM_000179:c.3802-40C>G	rs3136367	44,67	Known			
	**MSH6:NM_000179:c.1614_1616delTCT**	**.**	**58.33**	**US / PPAT**			
	MSH6:NM_000179:c.3646+29_3646+32delCTAT	rs2234731	42.86		Out of pre-NGS method covered region		
	MSH6:NM_000179:c.4002-27_4002-27delT	.	32.58		Out of pre-NGS method covered region		
V10							
	BRCA2:NM_000059:c.793+98G>A	rs206073	99,7		Out of pre-NGS method covered region		
	BRCA2:NM_000059:c.1114A>C	rs144848	47,61	Known			
	BRCA2:NM_000059:c.3807T>C	rs543304	47,72	Known			
	BRCA2:NM_000059:c.4563A>G	rs206075	100		Could not be detected by CSCE		
	BRCA2:NM_000059:c.6513G>C	rs206076	99,6		Could not be detected by CSCE		
	BRCA2:NM_000059:c.6938-120T>C	rs206080	99,17		Out of pre-NGS method covered region		
	BRCA2:NM_000059:c.7397T>C	rs169547	99,77		Could not be detected by CSCE		
	BRCA2:NM_000059:c.7617+190G>A	rs206096	100		Out of pre-NGS method covered region		
	BRCA2:NM_000059:c.9501+178A>G	rs206343	66,67		Out of pre-NGS method covered region		
	BRCA2:NM_000059:c.9648+244T>C	rs206344	100		Out of pre-NGS method covered region		
	BRCA2:NM_000059:c.10257+105A>C	rs15869	41,79		Out of pre-NGS method covered region		
	BRCA1:NM_007294:c.5407-193A>G	rs8176310	50		Out of pre-NGS method covered region		
	BRCA1:NM_007294:c.5278-191A>T	rs8176297	45,65		Out of pre-NGS method covered region		
	BRCA1:NM_007294:c.5152+66G>A	rs3092994	58,7		Out of pre-NGS method covered region		
	BRCA1:NM_007294:c.5075-237C>A	rs8176257	72,73		Out of pre-NGS method covered region		
	BRCA1:NM_007294:c.5074+65G>A	rs8176235	55,49	Known			
	BRCA1:NM_007294:c.4987-68A>G	rs8176234	54,55		Out of pre-NGS method covered region		
	BRCA1:NM_007294:c.4987-92A>G	rs8176233	48,15		Out of pre-NGS method covered region		
	BRCA1:NM_007294:c.4986+222A>G	rs3092987	81,82		Out of pre-NGS method covered region		
	BRCA1:NM_007294:c.4837A>G	rs1799966	48,29	Known			
	BRCA1:NM_007294:c.4485-63C>G	rs8176212	49,67		Out of pre-NGS method covered region		
	BRCA1:NM_007294:c.4485-137T>A	rs2236762	46,9		Out of pre-NGS method covered region		
	BRCA1:NM_007294:c.4358-2590T>G	rs8176194	51,53		Out of pre-NGS method covered region		
	BRCA1:NM_007294:c.4358-2885G>A	rs8176193	48,27		Out of pre-NGS method covered region		
	BRCA1:NM_007294:c.4308T>C	rs1060915	52,13	Known			
	BRCA1:NM_007294:c.4097-141A>C	rs799916	51,88		Out of pre-NGS method covered region		
	BRCA1:NM_007294:c.3548A>G	rs16942	49,79	Known			
	BRCA1:NM_007294:c.3113A>G	rs16941	50,39	Known			
	BRCA1:NM_007294:c.2612C>T	rs799917	49,51	Known			
	BRCA1:NM_007294:c.2311T>C	rs16940	47,28	Known			
	BRCA1:NM_007294:c.2082C>T	rs1799949	52,33	Known			
	BRCA1:NM_007294:c.594-225G>A	rs8176147	43,75		Out of pre-NGS method covered region		
	BRCA1:NM_007294:c.593+167T>C	rs8176145	50,94	Known			
	BRCA1:NM_007294:c.547+146A>T	rs8176140	63,64		Out of pre-NGS method covered region		
	BRCA1:NM_007294:c.213-161A>G	rs799912	52,94		Out of pre-NGS method covered region		
	BRCA1:NM_007294:c.1-134T>C	rs3765640	54,55		Out of pre-NGS method covered region		
	BRCA1:NM_007294:c.5468-121_5468-121delA	rs375078966	21.49		Out of pre-NGS method covered region		
	BRCA1:NM_007294:c.4485-203_4485-199delAACCC	rs34250703	40.74		Out of pre-NGS method covered region		

	BRCA1:NM_007294:c.548-58_548-58delT	rs8176144	51.58		Out of pre-NGS method covered region		
	BRCA1:NM_007294:c.441+52_441+63delCTTTTTTTTTTT	.	53.66		Out of pre-NGS method covered region		
	BRCA1:NM_007294:c.441+36_441+38delCTT	rs147856441	39.26		Out of pre-NGS method covered region		
	**BRCA1:NM_007294:c.68_69delAG**	**rs386833395**	**49.82**	**PAT**			
V11							
	BRCA2:NM_000059:c.1-26G>A	rs1799943	47,96	Known			
	BRCA2:NM_000059:c.425+246G>C	rs11571613	55		Out of pre-NGS method covered region		
	BRCA2:NM_000059:c.631+183T>A	rs3752451	44,44		Out of pre-NGS method covered region		
	BRCA2:NM_000059:c.793+98G>A	rs206073	99,16		Out of pre-NGS method covered region		
	BRCA2:NM_000059:c.1114A>C	rs144848	45,68	Known			
	BRCA2:NM_000059:c.3396A>G	rs1801406	51,67	Known			
	BRCA2:NM_000059:c.4563A>G	rs206075	100	Known			
	BRCA2:NM_000059:c.6513G>C	rs206076	99,83	Known			
	BRCA2:NM_000059:c.6841+191C>A	rs11571662	50		Out of pre-NGS method covered region		
	BRCA2:NM_000059:c.6938-120T>C	rs206080	99,71		Out of pre-NGS method covered region		
	BRCA2:NM_000059:c.7242A>G	rs1799955	49,01	Known			
	BRCA2:NM_000059:c.7397T>C	rs169547	99,24	Known			
	BRCA2:NM_000059:c.7617+190G>A	rs206096	97,22		Out of pre-NGS method covered region		
	BRCA2:NM_000059:c.7806-14T>C	rs9534262	99,63	Known			
	BRCA2:NM_000059:c.8754+183A>C	rs3764791	49,06		Out of pre-NGS method covered region		
	BRCA2:NM_000059:c.8754+187C>T	rs3764792	51,02		Out of pre-NGS method covered region		
	BRCA2:NM_000059:c.8755-66T>C	rs4942486	99,28		Out of pre-NGS method covered region		
	BRCA2:NM_000059:c.9257-219A>G	rs7330025	100		Out of pre-NGS method covered region		
	BRCA2:NM_000059:c.9648+244T>C	rs206344	100		Out of pre-NGS method covered region		
	BRCA2:NM_000059:c.6841+78_6841+81delAATT	rs138193280	43.32		Out of pre-NGS method covered region		
	BRCA2:NM_000059:c.8633-150_8633-149insA	rs200156944	32.26		Out of pre-NGS method covered region		
	BRCA1:NM_007294:c.3119G>A	rs4986852	47,96	Known			
	BRCA1:NM_007294:c.548-293G>A	rs117281398	57,14		Out of pre-NGS method covered region		
	BRCA1:NM_007294:c.442-34C>T	rs799923	53,02	Known			
	BRCA1:NM_007294:c.4987-142_4987-142delA	.	22.22		Out of pre-NGS method covered region		
V12							
	BRCA2:NM_000059:c.793+98G>A	rs206073	97,27		Out of pre-NGS method covered region		
	BRCA2:NM_000059:c.3807T>C	rs543304	99,35		Region not anlyzed by pre-NGS method		
	BRCA2:NM_000059:c.4563A>G	rs206075	100		Region not anlyzed by pre-NGS method		
	BRCA2:NM_000059:c.6513G>C	rs206076	100		Region not anlyzed by pre-NGS method		
	BRCA2:NM_000059:c.6938-120T>C	rs206080	100		Out of pre-NGS method covered region		
	BRCA2:NM_000059:c.7397T>C	rs169547	99		Region not anlyzed by pre-NGS method		
	BRCA2:NM_000059:c.7617+190G>A	rs206096	100		Out of pre-NGS method covered region		
	BRCA2:NM_000059:c.10257+105A>C	rs15869	100		Out of pre-NGS method covered region		
	**BRCA2:NM_000059:c.1307_1308delAG**	**.**	**40.95**	**PAT**			
V13							
	**PMS2:NM_000535:c.2606G>C (c.*17G>C)**	**.**	**Not detected**	**Known**	**Complex region**	**SANGER**	
	**PMS2:NM_000535:c.2681_2682insA (c.*92dupA)**	**rs3214300**	**Not detected**	**Known**	**Complex region**	**SANGER**	
	PMS2:NM_000535:c.2570G>C	rs1802683	36,5				
	PMS2:NM_000535:c.2466T>C	rs10000	41,48	Known			
	PMS2:NM_000535:c.2446-118G>A	.	33,33		Region not anlyzed by pre-NGS method		
	**PMS2:NM_000535:c.2340C>T**	**rs17420802**	**Not detected**	**Known**	**Complex region**	**SANGER**	
	**PMS2:NM_000535:c.2334A>G**	**rs63750220**	**Not detected**	**Known**	**Complex region**	**SANGER**	
	PMS2:NM_000535:c.2276-135T>C	rs9655490	62,5		Region not anlyzed by pre-NGS method		
	**PMS2:NM_000535:c.2253T>C**	**rs1805325**	**Not detected**	**Known**	**Variant only detected by RNA-based analysis**	**SANGER**	
	PMS2:NM_000535:c.2174+103C>T	rs2692553	29,09		Region not anlyzed by pre-NGS method		
	PMS2:NM_000535:c.2007-7C>T	rs55954143	40,17	Known			
	PMS2:NM_000535:c.1621A>G	rs2228006	100	Known			
	PMS2:NM_000535:c.1145-57G>A	rs199550985	20,42		Region not anlyzed by pre-NGS method		
	PMS2:NM_000535:c.780C>G	rs1805319	99,26	Known			
	PMS2:NM_000535:c.353+209G>A	rs6463526	61,9		Region not anlyzed by pre-NGS method		
	PMS2:NM_000535:c.251-72A>G	rs117831773	48,33		Region not anlyzed by pre-NGS method		
	**PMS2:NM_000535:c.59G>A**	**rs10254120**	**52,23**	**PAT**			

	PMS2:NM_000535:c.23+72C>T	rs3735295	34,21	Known			
	PMS2:NM_000535:c.705+118_705+118delT	rs11289444	53.7		Region not anlyzed by pre-NGS method		
V14							
	BRCA2:NM_000059:c.793+98G>A	rs206073	98,49		Out of pre-NGS method covered region		
	BRCA2:NM_000059:c.1114A>C	rs144848	99,69		Region not anlyzed by pre-NGS method		
	BRCA2:NM_000059:c.4563A>G	rs206075	100		Region not anlyzed by pre-NGS method		
	BRCA2:NM_000059:c.6513G>C	rs206076	99,43		Region not anlyzed by pre-NGS method		
	BRCA2:NM_000059:c.6938-120T>C	rs206080	97,83		Out of pre-NGS method covered region		
	BRCA2:NM_000059:c.7397T>C	rs169547	99,52		Region not anlyzed by pre-NGS method		
	BRCA2:NM_000059:c.7617+190G>A	rs206096	100		Out of pre-NGS method covered region		
	BRCA2:NM_000059:c.7806-14T>C	rs9534262	47,44		Region not anlyzed by pre-NGS method		
	BRCA2:NM_000059:c.8755-66T>C	rs4942486	49,81		Out of pre-NGS method covered region		
	**BRCA2:NM_000059:c.3934_3952delAATTACAAGAGAAATACTG**	**.**	**35.53**	**PAT**			
V15							
	APC:NM_001127510:c.423-256G>A	rs392179	100		Out of pre-NGS method covered region		
	APC:NM_001127510:c.645+173A>G	rs2289484	100	Known			
	APC:NM_001127510:c.730-22G>C	rs115634618	47,09	Known			
	APC:NM_001127510:c.934-132G>T	rs12521276	100		Out of pre-NGS method covered region		
	APC:NM_001127510:c.1458T>C	rs2229992	100	Known			
	APC:NM_001127510:c.1635G>A	rs351771	99,73	Known			
	APC:NM_001127510:c.1743+193G>A	rs351772	100	Known			
	**APC:NM_001127510:c.2344A>T**	**.**	**50,63**	**PAT**			
	APC:NM_001127510:c.4479G>A	rs41115	99,4	Known			
	APC:NM_001127510:c.5034G>A	rs42427	99,47	Known			
	APC:NM_001127510:c.5268T>G	rs866006	100	Known			
	APC:NM_001127510:c.5465T>A	rs459552	99,42	Known			
	APC:NM_001127510:c.5880G>A	rs465899	100	Known			
	APC:NM_001127510:c.934-133_934-132delTG	rs369189394	20		Out of pre-NGS method covered region		
	APC:NM_001127510:c.934-132_934-132delG	rs141013063	65.31		Out of pre-NGS method covered region		
	APC:NM_001127510:c.934-120_934-119insG	rs138003874	77.42		Out of pre-NGS method covered region		
	APC:NM_001127510:c.1959-145_1959-144insAGAA	rs3839284	93.58		Out of pre-NGS method covered region		
V16							
	MUTYH:NM_012222:c.1477-40C>G	rs3219493	99,54		Region not anlyzed by pre-NGS method		
	**MUTYH:NM_012222:c.1187G>A**	**rs36053993**	**100**	**PAT**			
	MUTYH:NM_012222:c.504+35A>G	rs3219487	100		Region not anlyzed by pre-NGS method		
V17							
	BRCA2:NM_000059:c.793+98G>A	rs206073	99,35		Out of pre-NGS method covered region		
	BRCA2:NM_000059:c.1114A>C	rs144848	48,33	Known			
	BRCA2:NM_000059:c.3807T>C	rs543304	47,13	Known			
	BRCA2:NM_000059:c.4563A>G	rs206075	100		Could not be detected by CSCE		
	BRCA2:NM_000059:c.6513G>C	rs206076	100		Could not be detected by CSCE		
	BRCA2:NM_000059:c.6938-120T>C	rs206080	99,12		Out of pre-NGS method covered region		
	BRCA2:NM_000059:c.7397T>C	rs169547	99,5		Could not be detected by CSCE		
	BRCA2:NM_000059:c.7617+190G>A	rs206096	100		Out of pre-NGS method covered region		
	BRCA2:NM_000059:c.9501+178A>G	rs206343	34,62		Out of pre-NGS method covered region		
	BRCA2:NM_000059:c.10257+105A>C	rs15869	63,75		Out of pre-NGS method covered region		
	**BRCA2:NM_000059:c.5718_5719delCT**	**rs80359530**	**49.73**	**PAT**			
	BRCA1:NM_007294:c.4987-142_4987-142delA	.	23.68		Out of pre-NGS method covered region		
V18							
	BRCA2:NM_000059:c.1-26G>A	rs1799943	49,85	Known			
	BRCA2:NM_000059:c.631+183T>A	rs3752451	47,06		Out of pre-NGS method covered region		
	BRCA2:NM_000059:c.793+98G>A	rs206073	99,27		Out of pre-NGS method covered region		
	BRCA2:NM_000059:c.1114A>C	rs144848	47,34	Known			
	BRCA2:NM_000059:c.3396A>G	rs1801406	43,85	Known			
	BRCA2:NM_000059:c.4563A>G	rs206075	100	Known			
	BRCA2:NM_000059:c.6513G>C	rs206076	99,81	Known			
	BRCA2:NM_000059:c.6841+191C>A	rs11571662	54,65		Out of pre-NGS method covered region		
	BRCA2:NM_000059:c.6938-120T>C	rs206080	100		Out of pre-NGS method covered region		

	BRCA2:NM_000059:c.7242A>G	rs1799955	48,96	Known			
	BRCA2:NM_000059:c.7397T>C	rs169547	99,61	Known			
	BRCA2:NM_000059:c.7617+190G>A	rs206096	100		Out of pre-NGS method covered region		
	BRCA2:NM_000059:c.7806-14T>C	rs9534262	48,26	Known			
	BRCA2:NM_000059:c.8754+183A>C	rs3764791	38,64		Out of pre-NGS method covered region		
	BRCA2:NM_000059:c.8754+187C>T	rs3764792	37,84		Out of pre-NGS method covered region		
	BRCA2:NM_000059:c.8755-66T>C	rs4942486	47,33		Out of pre-NGS method covered region		
	BRCA2:NM_000059:c.9501+178A>G	rs206343	48,28		Out of pre-NGS method covered region		
	BRCA2:NM_000059:c.10257+105A>C	rs15869	64,29		Out of pre-NGS method covered region		
	BRCA2:NM_000059:c.6841+78_6841+81delAATT	rs138193280	45,78		Out of pre-NGS method covered region		
	BRCA1:NM_007294:c.2584A>G	rs80356927	47,47	Known			
	BRCA1:NM_007294:c.442-34C>T	rs799923	50,78	Known			
	BRCA1:NM_007294:c.4987-142_4987-142delA	.	26.58		Out of pre-NGS method covered region		
	**BRCA1:NM_007294:c.3869_3870delAA**	**rs80357918**	**51.32**	**PAT**			
V19							
	MSH2:NM_000251:c.211+9C>G	rs2303426	48,72		Region not anlyzed by pre-NGS method		
	MSH2:NM_000251:c.367-86A>C	rs17217765	46,15		Out of pre-NGS method covered region		
	**MSH2:NM_000251:c.691G>T**	**.**	**46,99**	**PAT**	Region not anlyzed by pre-NGS method		
	MSH2:NM_000251:c.1277-118G>A	rs1981929	46,95		Out of pre-NGS method covered region		
	MSH2:NM_000251:c.1661+90T>C	rs10183143	68,57		Out of pre-NGS method covered region		
	MSH2:NM_000251:c.2006-127C>T	rs2059521	100		Out of pre-NGS method covered region		
	**MSH2:NM_000251:c.689_689delC**	**.**	**45.2**	**PAT**			
	MSH2:NM_000251:c.2005+43_2005+43delT	rs201305711	22.78		Region not anlyzed by pre-NGS method		
	MLH1:NM_000249:c.453+79A>G	rs4234259	51,39		Out of pre-NGS method covered region		
	MLH1:NM_000249:c.655A>G	rs1799977	55,83	Known			
	MLH1:NM_000249:c.1038+86T>C	rs2286939	65,62		Out of pre-NGS method covered region		
	MLH1:NM_000249:c.1039-78A>G	rs11129748	60		Out of pre-NGS method covered region		
	MLH1:NM_000249:c.1668-19A>G	rs9876116	42,14		Region not anlyzed by pre-NGS method		
	MLH1:NM_000249:c.1990-121C>T	rs2241031	55,36		Out of pre-NGS method covered region		
	MLH1:NM_000249:c.1897-167_1897-167delT	rs11290150	85,81		Out of pre-NGS method covered region		
V20							
	PMS2:NM_000535:c.2446-118G>A	.	27,59		Out of pre-NGS method covered region		
	PMS2:NM_000535:c.2276-135T>C	rs9655490	39,23		Out of pre-NGS method covered region		
	PMS2:NM_000535:c.1621A>G	rs2228006	99,79	Known			
	PMS2:NM_000535:c.1408C>T	rs1805321	56,54	Known			
	PMS2:NM_000535:c.804-164T>G	rs2286681	61,54		Out of pre-NGS method covered region		
	PMS2:NM_000535:c.780C>G	rs1805319	99,11	Known			
	PMS2:NM_000535:c.705+17A>G	rs62456182	50	Known			
	PMS2:NM_000535:c.538-223A>G	rs2345060	75		Out of pre-NGS method covered region		
	PMS2:NM_000535:c.163+138A>G	rs62456183	70		Out of pre-NGS method covered region		
	PMS2:NM_000535:c.989-105_989-104insA	rs149249785	36		Out of pre-NGS method covered region		
	**PMS2:NM_000535:c.780_780delC**	**.**	**49.53**	**PAT**			
	PMS2:NM_000535:c.705+118_705+118delT	rs11289444	48.84		Out of pre-NGS method covered region		
V21							
	MLH1:NM_000249:c.1-93G>A	rs1800734	100	Known			
	MLH1:NM_000249:c.116+199C>T	.	40,91		Out of pre-NGS method covered region		
	MLH1:NM_000249:c.1039-31A>T	rs59684491	43,75		Region not anlyzed by pre-NGS method		
	MLH1:NM_000249:c.1039-29A>T	rs6771325	73,68		Region not anlyzed by pre-NGS method		
	MLH1:NM_000249:c.1039-31_1039-29delATA	.	26.92		Region not anlyzed by pre-NGS method		
V22							
	BRCA2:NM_000059:c.793+98G>A	rs206073	99,61		Out of pre-NGS method covered region		
	BRCA2:NM_000059:c.1114A>C	rs144848	99,42	Known			
	BRCA2:NM_000059:c.4563A>G	rs206075	100	Known			
	BRCA2:NM_000059:c.6513G>C	rs206076	99,66	Known			
	BRCA2:NM_000059:c.6938-120T>C	rs206080	98,37		Out of pre-NGS method covered region		
	BRCA2:NM_000059:c.7397T>C	rs169547	100	Known			
	BRCA2:NM_000059:c.7617+190G>A	rs206096	100		Out of pre-NGS method covered region		
	BRCA2:NM_000059:c.9648+84G>A	rs81002832	40,86		Out of pre-NGS method covered region		

	BRCA1:NM_007294:c.5407-193A>G	rs8176310	38,71		Out of pre-NGS method covered region		
	BRCA1:NM_007294:c.5278-191A>T	rs8176297	59,57		Out of pre-NGS method covered region		
	BRCA1:NM_007294:c.5152+66G>A	rs3092994	42,2		Out of pre-NGS method covered region		
	BRCA1:NM_007294:c.5075-237C>A	rs8176257	53,85		Out of pre-NGS method covered region		
	BRCA1:NM_007294:c.5074+65G>A	rs8176235	50,44		Out of pre-NGS method covered region		
	BRCA1:NM_007294:c.4987-68A>G	rs8176234	52,94		Out of pre-NGS method covered region		
	BRCA1:NM_007294:c.4987-92A>G	rs8176233	50		Out of pre-NGS method covered region		
	BRCA1:NM_007294:c.4986+222A>G	rs3092987	77,78		Out of pre-NGS method covered region		
	BRCA1:NM_007294:c.4837A>G	rs1799966	51,23	Known			
	BRCA1:NM_007294:c.4485-63C>G	rs8176212	50,72		Out of pre-NGS method covered region		
	BRCA1:NM_007294:c.4485-137T>A	rs2236762	53,25		Out of pre-NGS method covered region		
	BRCA1:NM_007294:c.4358-2590T>G	rs8176194	47,86		Out of pre-NGS method covered region		
	BRCA1:NM_007294:c.4358-2885G>A	rs8176193	48,88		Out of pre-NGS method covered region		
	BRCA1:NM_007294:c.4357+117G>A	rs3737559	41,79		Out of pre-NGS method covered region		
	BRCA1:NM_007294:c.4308T>C	rs1060915	46,19	Known			
	BRCA1:NM_007294:c.4097-141A>C	rs799916	45,81		Out of pre-NGS method covered region		
	BRCA1:NM_007294:c.3548A>G	rs16942	47,99	Known			
	BRCA1:NM_007294:c.3113A>G	rs16941	47,08	Known			
	BRCA1:NM_007294:c.2612C>T	rs799917	47,71	Known			
	BRCA1:NM_007294:c.2311T>C	rs16940	49,37	Known			
	BRCA1:NM_007294:c.2082C>T	rs1799949	47,7	Known			
	BRCA1:NM_007294:c.594-225G>A	rs8176147	69,23		Out of pre-NGS method covered region		
	BRCA1:NM_007294:c.593+167T>C	rs8176145	46,81		Out of pre-NGS method covered region		
	BRCA1:NM_007294:c.547+146A>T	rs8176140	56,52		Out of pre-NGS method covered region		
	BRCA1:NM_007294:c.213-161A>G	rs799912	43,24		Out of pre-NGS method covered region		
	BRCA1:NM_007294:c.1-134T>C	rs3765640	60		Out of pre-NGS method covered region		
	BRCA1:NM_007294:c.4485-203_4485-199delAACCC	rs34250703	53.85		Out of pre-NGS method covered region		
	**BRCA1:NM_007294:c.3770_3771delAG**	**rs80357993**	**50.44**	**PAT**			
	BRCA1:NM_007294:c.548-58_548-58delT	rs8176144	46.24		Out of pre-NGS method covered region		
	BRCA1:NM_007294:c.441+52_441+63delCTTTTTTTTTTT	.	37.04		Out of pre-NGS method covered region		
	BRCA1:NM_007294:c.441+36_441+38delCTT	rs147856441	46.15		Out of pre-NGS method covered region		
	BRCA1:NM_007294:c.134+224_134+224delT	rs35149296	46.15		Out of pre-NGS method covered region		
V23							
	BRCA2:NM_000059:c.793+98G>A	rs206073	100		Out of pre-NGS method covered region		
	BRCA2:NM_000059:c.1114A>C	rs144848	54,95		Region not anlyzed by pre-NGS method		
	BRCA2:NM_000059:c.3807T>C	rs543304	42,21		Region not anlyzed by pre-NGS method		
	BRCA2:NM_000059:c.4563A>G	rs206075	100		Region not anlyzed by pre-NGS method		
	BRCA2:NM_000059:c.6513G>C	rs206076	100		Region not anlyzed by pre-NGS method		
	BRCA2:NM_000059:c.6938-120T>C	rs206080	100		Out of pre-NGS method covered region		
	BRCA2:NM_000059:c.7397T>C	rs169547	99,43		Region not anlyzed by pre-NGS method		
	BRCA2:NM_000059:c.7617+190G>A	rs206096	100		Out of pre-NGS method covered region		
	BRCA2:NM_000059:c.7806-14T>C	rs9534262	49,52		Region not anlyzed by pre-NGS method		
	BRCA2:NM_000059:c.8755-66T>C	rs4942486	50		Out of pre-NGS method covered region		
	BRCA2:NM_000059:c.10257+105A>C	rs15869	59,09		Out of pre-NGS method covered region		
	**BRCA2:NM_000059:c.1126_1126delT**	**.**	**43,55**	**PAT**			
V24							
	APC:NM_001127510:c.423-256G>A	rs392179	100		Out of pre-NGS method covered region		
	APC:NM_001127510:c.645+173A>G	rs2289484	100		Out of pre-NGS method covered region		
	APC:NM_001127510:c.933+261G>T	rs12656359	100		Out of pre-NGS method covered region		
	APC:NM_001127510:c.934-132G>T	rs12521276	100		Out of pre-NGS method covered region		
	APC:NM_001127510:c.1458T>C	rs2229992	100		Region not anlyzed by pre-NGS method		
	APC:NM_001127510:c.1635G>A	rs351771	99,68		Region not anlyzed by pre-NGS method		
	APC:NM_001127510:c.1743+193G>A	rs351772	100		Out of pre-NGS method covered region		
	APC:NM_001127510:c.3249T>G	rs201629780	45,35		Region not anlyzed by pre-NGS method		
	APC:NM_001127510:c.4479G>A	rs41115	100		Region not anlyzed by pre-NGS method		
	APC:NM_001127510:c.5034G>A	rs42427	100		Region not anlyzed by pre-NGS method		
	APC:NM_001127510:c.5268T>G	rs866006	99,65		Region not anlyzed by pre-NGS method		
	APC:NM_001127510:c.5465T>A	rs459552	100		Region not anlyzed by pre-NGS method		

	APC:NM_001127510:c.5880G>A	rs465899	100		Region not anlyzed by pre-NGS method		
	APC:NM_001127510:c.934-133_934-132delTG	rs369189394	28.05		Out of pre-NGS method covered region		
	APC:NM_001127510:c.934-132_934-132delG	rs141013063	67.8		Out of pre-NGS method covered region		
	APC:NM_001127510:c.934-120_934-119insG	rs138003874	89.52		Out of pre-NGS method covered region		
	APC:NM_001127510:c.1959-145_1959-144insAGAA	rs3839284	97.56		Out of pre-NGS method covered region		
	**APC:NM_001127510:c.3180_3184delAAAAC**	**.**	**53.1**	**PAT**			
V25							
	MSH2:NM_000251:c.1661+90T>C	rs10183143	37,93		Out of pre-NGS method covered region		
	MSH2:NM_000251:c.2006-127C>T	rs2059521	100		Out of pre-NGS method covered region		
	**MSH2:NM_000251:c.388_389delCA**	**rs63750704**	**45.72**	**PAT**			
	MSH2:NM_000251:c.2005+43_2005+43delT	rs201305711	21.5		Out of pre-NGS method covered region		
V26							
	BRCA2:NM_000059:c.793+98G>A	rs206073	100		Out of pre-NGS method covered region		
	BRCA2:NM_000059:c.1114A>C	rs144848	50,84		Region not anlyzed by pre-NGS method		
	BRCA2:NM_000059:c.3807T>C	rs543304	47,4		Region not anlyzed by pre-NGS method		
	BRCA2:NM_000059:c.4563A>G	rs206075	100		Region not anlyzed by pre-NGS method		
	BRCA2:NM_000059:c.6513G>C	rs206076	100		Region not anlyzed by pre-NGS method		
	BRCA2:NM_000059:c.6938-120T>C	rs206080	99,6		Out of pre-NGS method covered region		
	BRCA2:NM_000059:c.7397T>C	rs169547	100		Region not anlyzed by pre-NGS method		
	BRCA2:NM_000059:c.7617+190G>A	rs206096	100		Out of pre-NGS method covered region		
	BRCA2:NM_000059:c.7806-14T>C	rs9534262	49,51		Region not anlyzed by pre-NGS method		
	BRCA2:NM_000059:c.8755-66T>C	rs4942486	51,45		Out of pre-NGS method covered region		
	BRCA2:NM_000059:c.9257-235G>A	rs7327867	63,64		Out of pre-NGS method covered region		
	BRCA2:NM_000059:c.9257-219A>G	rs7330025	50		Out of pre-NGS method covered region		
	BRCA2:NM_000059:c.9501+178A>G	rs206343	50		Out of pre-NGS method covered region		
	BRCA2:NM_000059:c.9648+244T>C	rs206344	100		Out of pre-NGS method covered region		
	BRCA2:NM_000059:c.10257+105A>C	rs15869	47,93		Out of pre-NGS method covered region		
	**BRCA2:NM_000059:c.7227_7227delT**	**.**	**42,31**	**PAT**			
	BRCA1:NM_007294:c.5407-193A>G	rs8176310	75,93		Out of pre-NGS method covered region		
	BRCA1:NM_007294:c.5333-153A>G	rs8176305	45,71		Out of pre-NGS method covered region		
	BRCA1:NM_007294:c.5278-191A>T	rs8176297	53,27		Out of pre-NGS method covered region		
	BRCA1:NM_007294:c.5152+66G>A	rs3092994	48,16		Out of pre-NGS method covered region		
	BRCA1:NM_007294:c.5075-238A>G	rs8176256	66,67		Out of pre-NGS method covered region		
	BRCA1:NM_007294:c.4987-68A>G	rs8176234	46,86		Out of pre-NGS method covered region		
	BRCA1:NM_007294:c.4987-92A>G	rs8176233	46,34		Out of pre-NGS method covered region		
	BRCA1:NM_007294:c.4986+222A>G	rs3092987	54,17		Out of pre-NGS method covered region		
	BRCA1:NM_007294:c.4837A>G	rs1799966	47,99		Region not anlyzed by pre-NGS method		
	BRCA1:NM_007294:c.4485-63C>G	rs8176212	51,8		Out of pre-NGS method covered region		
	BRCA1:NM_007294:c.4485-137T>A	rs2236762	51,2		Out of pre-NGS method covered region		
	BRCA1:NM_007294:c.4358-2590T>G	rs8176194	50,92		Out of pre-NGS method covered region		
	BRCA1:NM_007294:c.4358-2885G>A	rs8176193	50		Out of pre-NGS method covered region		
	BRCA1:NM_007294:c.4308T>C	rs1060915	48,09		Region not anlyzed by pre-NGS method		
	BRCA1:NM_007294:c.4097-141A>C	rs799916	42,68		Out of pre-NGS method covered region		
	BRCA1:NM_007294:c.3548A>G	rs16942	50,19		Region not anlyzed by pre-NGS method		
	BRCA1:NM_007294:c.3113A>G	rs16941	47,86		Region not anlyzed by pre-NGS method		
	BRCA1:NM_007294:c.2612C>T	rs799917	45,07		Region not anlyzed by pre-NGS method		
	BRCA1:NM_007294:c.2311T>C	rs16940	48,22		Region not anlyzed by pre-NGS method		
	BRCA1:NM_007294:c.2082C>T	rs1799949	52,39		Region not anlyzed by pre-NGS method		
	BRCA1:NM_007294:c.2077G>A	rs4986850	52,9		Region not anlyzed by pre-NGS method		
	BRCA1:NM_007294:c.594-225G>A	rs8176147	62,07		Out of pre-NGS method covered region		
	BRCA1:NM_007294:c.593+167T>C	rs8176145	48,18		Out of pre-NGS method covered region		
	BRCA1:NM_007294:c.547+146A>T	rs8176140	66,67		Out of pre-NGS method covered region		
	BRCA1:NM_007294:c.442-34C>T	rs799923	43,84		Region not anlyzed by pre-NGS method		
	BRCA1:NM_007294:c.213-161A>G	rs799912	37,08		Out of pre-NGS method covered region		
	BRCA1:NM_007294:c.1-134T>C	rs3765640	57,78		Out of pre-NGS method covered region		
	BRCA1:NM_007294:c.4485-203_4485-199delAACCC	rs34250703	27.27		Out of pre-NGS method covered region		
	BRCA1:NM_007294:c.548-58_548-58delT	rs8176144	45.99		Out of pre-NGS method covered region		
	BRCA1:NM_007294:c.441+52_441+63delCTTTTTTTTTTT	.	44.23		Out of pre-NGS method covered region		

	BRCA1:NM_007294:c.441+36_441+38delCTT	rs147856441	44.89		Region not anlyzed by pre-NGS method		
	BRCA1:NM_007294:c.134+224_134+224delT	rs35149296	59.09		Out of pre-NGS method covered region		
V27							
	MSH6:NM_000179:c.186C>A	rs1042820	58,18	Known			
	MSH6:NM_000179:c.260-22C>G		**Not detected**	**Known**	**Low covered region**	**SANGER**	
	MSH6:NM_000179:c.276A>G	rs1800932	51,93	Known			
	MSH6:NM_000179:c.457+52T>A	rs3136282	55,36	Known			
	MSH6:NM_000179:c.458-133A>G	rs3136316	58,65	Known			
	MSH6:NM_000179:c.540T>C	rs1800935	51,34	Known			
	MSH6:NM_000179:c.628-56C>T	rs1800936	45,11	Known			
	MSH6:NM_000179:c.3173-101G>C	rs2072447	49,04		Out of pre-NGS method covered region		
	MSH6:NM_000179:c.3438+14A>T	rs2020911	49,63	Known			
	MSH6:NM_000179:c.3556+146G>A	rs7562048	42,55	Known			
	MSH6:NM_000179:c.3557-144G>A	rs7562367	66,67		Out of pre-NGS method covered region		
	MSH6:NM_000179:c.3646+91T>C	rs3136359	48,98		Out of pre-NGS method covered region		
	MSH6:NM_000179:c.3802-40C>G	rs3136367	49,02	Known			
	**MSH6:NM_000179:c.2905_2906delTA**	**.**	**49.4**	**PAT**			
	MSH6:NM_000179:c.3646+29_3646+32delCTAT	rs2234731	41.11	Known			
	MSH6:NM_000179:c.4002-27_4002-27delT	.	33.27				
V28							
	BRCA2:NM_000059:c.425+246G>C	rs11571613	57,89		Out of pre-NGS method covered region		
	BRCA2:NM_000059:c.631+183T>A	rs3752451	63,64		Out of pre-NGS method covered region		
	BRCA2:NM_000059:c.793+98G>A	rs206073	99,55		Out of pre-NGS method covered region		
	BRCA2:NM_000059:c.3396A>G	rs1801406	46,92	Known			
	BRCA2:NM_000059:c.3807T>C	rs543304	49,13	Known			
	BRCA2:NM_000059:c.4563A>G	rs206075	100		Could not be detected by CSCE		
	BRCA2:NM_000059:c.6513G>C	rs206076	100		Could not be detected by CSCE		
	BRCA2:NM_000059:c.6841+191C>A	rs11571662	51,47		Out of pre-NGS method covered region		
	BRCA2:NM_000059:c.6938-120T>C	rs206080	100		Out of pre-NGS method covered region		
	BRCA2:NM_000059:c.7242A>G	rs1799955	48,74	Known			
	BRCA2:NM_000059:c.7397T>C	rs169547	100		Could not be detected by CSCE		
	BRCA2:NM_000059:c.7617+190G>A	rs206096	100		Out of pre-NGS method covered region		
	BRCA2:NM_000059:c.7806-14T>C	rs9534262	52,33	Known			
	BRCA2:NM_000059:c.8754+183A>C	rs3764791	36,36		Out of pre-NGS method covered region		
	BRCA2:NM_000059:c.8754+187C>T	rs3764792	36,67		Out of pre-NGS method covered region		
	BRCA2:NM_000059:c.8755-66T>C	rs4942486	47,32		Out of pre-NGS method covered region		
	BRCA2:NM_000059:c.9257-219A>G	rs7330025	45		Out of pre-NGS method covered region		
	BRCA2:NM_000059:c.9501+178A>G	rs206343	61,9		Out of pre-NGS method covered region		
	BRCA2:NM_000059:c.9648+244T>C	rs206344	100		Out of pre-NGS method covered region		
	BRCA2:NM_000059:c.10257+105A>C	rs15869	55,81		Out of pre-NGS method covered region		
	**BRCA2:NM_000059:c.260_261delCT**	**.**	**44.9**	**PAT**			
	BRCA2:NM_000059:c.6841+78_6841+81delAATT	rs138193280	46.06		Out of pre-NGS method covered region		
	BRCA2:NM_000059:c.8633-150_8633-149insA	rs200156944	36.11		Out of pre-NGS method covered region		
	BRCA1:NM_007294:c.5407-193A>G	rs8176310	49,38		Out of pre-NGS method covered region		
	BRCA1:NM_007294:c.5333-153A>G	rs8176305	54,98		Out of pre-NGS method covered region		
	BRCA1:NM_007294:c.5278-191A>T	rs8176297	48,85		Out of pre-NGS method covered region		
	BRCA1:NM_007294:c.5152+66G>A	rs3092994	52,53		Out of pre-NGS method covered region		
	BRCA1:NM_007294:c.5075-238A>G	rs8176256	51,43		Out of pre-NGS method covered region		
	BRCA1:NM_007294:c.4987-68A>G	rs8176234	43,29		Out of pre-NGS method covered region		
	BRCA1:NM_007294:c.4987-92A>G	rs8176233	42,49		Out of pre-NGS method covered region		
	BRCA1:NM_007294:c.4986+222A>G	rs3092987	60		Out of pre-NGS method covered region		
	BRCA1:NM_007294:c.4837A>G	rs1799966	55,39	Known			
	BRCA1:NM_007294:c.4485-63C>G	rs8176212	46,72		Out of pre-NGS method covered region		
	BRCA1:NM_007294:c.4485-137T>A	rs2236762	50		Out of pre-NGS method covered region		
	BRCA1:NM_007294:c.4358-2590T>G	rs8176194	50		Out of pre-NGS method covered region		
	BRCA1:NM_007294:c.4358-2885G>A	rs8176193	49,27		Out of pre-NGS method covered region		
	BRCA1:NM_007294:c.4308T>C	rs1060915	45,37	Known			
	BRCA1:NM_007294:c.4097-141A>C	rs799916	53,38		Out of pre-NGS method covered region		

	BRCA1:NM_007294:c.3548A>G	rs16942	44,53	Known			
	BRCA1:NM_007294:c.3113A>G	rs16941	51,03	Known			
	BRCA1:NM_007294:c.2612C>T	rs799917	47,68	Known			
	BRCA1:NM_007294:c.2311T>C	rs16940	50,74	Known			
	BRCA1:NM_007294:c.2082C>T	rs1799949	50,65	Known			
	BRCA1:NM_007294:c.2077G>A	rs4986850	50,59	Known			
	BRCA1:NM_007294:c.671-178G>A	rs8176151	48,84		Out of pre-NGS method covered region		
	BRCA1:NM_007294:c.670+259T>G	rs7503154	50		Out of pre-NGS method covered region		
	BRCA1:NM_007294:c.594-225G>A	rs8176147	56,25		Out of pre-NGS method covered region		
	BRCA1:NM_007294:c.593+167T>C	rs8176145	53,01	Known			
	BRCA1:NM_007294:c.547+146A>T	rs8176140	51,06		Out of pre-NGS method covered region		
	BRCA1:NM_007294:c.213-161A>G	rs799912	41,76		Out of pre-NGS method covered region		
	BRCA1:NM_007294:c.1-134T>C	rs3765640	44,12	Known			
	BRCA1:NM_007294:c.4485-203_4485-199delAACCC	rs34250703	32.65		Out of pre-NGS method covered region		
	BRCA1:NM_007294:c.548-58_548-58delT	rs8176144	44.24		Out of pre-NGS method covered region		
	BRCA1:NM_007294:c.441+52_441+63delCTTTTTTTTTTT	0	48.33		Out of pre-NGS method covered region		
	BRCA1:NM_007294:c.441+36_441+38delCTT	rs147856441	42.96		Out of pre-NGS method covered region		
	BRCA1:NM_007294:c.134+224_134+224delT	rs35149296	46.15		Out of pre-NGS method covered region		
V29							
	BRCA2:NM_000059:c.1-26G>A	rs1799943	100		Could not be detected by CSCE		
	BRCA2:NM_000059:c.67+82C>G	rs189026060	50,34		Out of pre-NGS method covered region		
	BRCA2:NM_000059:c.631+183T>A	rs3752451	100		Out of pre-NGS method covered region		
	BRCA2:NM_000059:c.793+98G>A	rs206073	100		Out of pre-NGS method covered region		
	BRCA2:NM_000059:c.3396A>G	rs1801406	100		Could not be detected by CSCE		
	BRCA2:NM_000059:c.4563A>G	rs206075	100		Could not be detected by CSCE		
	BRCA2:NM_000059:c.6513G>C	rs206076	99,17		Could not be detected by CSCE		
	BRCA2:NM_000059:c.6841+191C>A	rs11571662	100		Out of pre-NGS method covered region		
	BRCA2:NM_000059:c.6938-120T>C	rs206080	100		Out of pre-NGS method covered region		
	BRCA2:NM_000059:c.7242A>G	rs1799955	100		Could not be detected by CSCE		
	BRCA2:NM_000059:c.7397T>C	rs169547	100		Could not be detected by CSCE		
	BRCA2:NM_000059:c.7617+190G>A	rs206096	100		Out of pre-NGS method covered region		
	BRCA2:NM_000059:c.7806-14T>C	rs9534262	100		Could not be detected by CSCE		
	BRCA2:NM_000059:c.8754+183A>C	rs3764791	100		Out of pre-NGS method covered region		
	BRCA2:NM_000059:c.8754+187C>T	rs3764792	100		Out of pre-NGS method covered region		
	BRCA2:NM_000059:c.8755-66T>C	rs4942486	96,3		Out of pre-NGS method covered region		
	BRCA2:NM_000059:c.9257-235G>A	rs7327867	100		Out of pre-NGS method covered region		
	BRCA2:NM_000059:c.9257-219A>G	rs7330025	100		Out of pre-NGS method covered region		
	BRCA2:NM_000059:c.6841+78_6841+81delAATT	rs138193280	87.25		Out of pre-NGS method covered region		
	BRCA2:NM_000059:c.8633-150_8633-149insA	rs200156944	84.62		Out of pre-NGS method covered region		
	BRCA1:NM_007294:c.5468-121_5468-121delA	rs375078966	24.62		Out of pre-NGS method covered region		
	**BRCA1:NM_007294:c.4110_4111insATCT**	**.**	**47.21**	**PAT**			
V30							
	BRCA2:NM_000059:c.1-26G>A	rs1799943	52,65	Known			
	BRCA2:NM_000059:c.425+246G>C	rs11571613	70		Out of pre-NGS method covered region		
	BRCA2:NM_000059:c.631+183T>A	rs3752451	53,19		Out of pre-NGS method covered region		
	BRCA2:NM_000059:c.793+98G>A	rs206073	100		Out of pre-NGS method covered region		
	BRCA2:NM_000059:c.1114A>C	rs144848	47,2	Known			
	BRCA2:NM_000059:c.3396A>G	rs1801406	45,99	Known			
	BRCA2:NM_000059:c.4563A>G	rs206075	100	Known			
	BRCA2:NM_000059:c.6513G>C	rs206076	99,8	Known			
	BRCA2:NM_000059:c.6841+191C>A	rs11571662	53,79		Out of pre-NGS method covered region		
	BRCA2:NM_000059:c.6938-120T>C	rs206080	99,55		Out of pre-NGS method covered region		
	BRCA2:NM_000059:c.7242A>G	rs1799955	46,14	Known			
	BRCA2:NM_000059:c.7397T>C	rs169547	100	Known			
	BRCA2:NM_000059:c.7617+190G>A	rs206096	100		Out of pre-NGS method covered region		
	BRCA2:NM_000059:c.7806-14T>C	rs9534262	51,41	Known			
	BRCA2:NM_000059:c.8754+183A>C	rs3764791	44,83		Out of pre-NGS method covered region		
	BRCA2:NM_000059:c.8754+187C>T	rs3764792	42,86		Out of pre-NGS method covered region		

	BRCA2:NM_000059:c.8755-66T>C	rs4942486	46,47		Out of pre-NGS method covered region		
	BRCA2:NM_000059:c.9257-219A>G	rs7330025	60		Out of pre-NGS method covered region		
	BRCA2:NM_000059:c.9648+244T>C	rs206344	100		Out of pre-NGS method covered region		
	BRCA2:NM_000059:c.6841+78_6841+81delAATT	rs138193280	41,61		Out of pre-NGS method covered region		
	BRCA1:NM_007294:c.5407-193A>G	rs8176310	48,57		Out of pre-NGS method covered region		
	BRCA1:NM_007294:c.5333-153A>G	rs8176305	44,14		Out of pre-NGS method covered region		
	BRCA1:NM_007294:c.5278-191A>T	rs8176297	57,14		Out of pre-NGS method covered region		
	BRCA1:NM_007294:c.5152+66G>A	rs3092994	57		Out of pre-NGS method covered region		
	BRCA1:NM_007294:c.5075-238A>G	rs8176256	64,29		Out of pre-NGS method covered region		
	BRCA1:NM_007294:c.4987-68A>G	rs8176234	60		Out of pre-NGS method covered region		
	BRCA1:NM_007294:c.4987-92A>G	rs8176233	63,64		Out of pre-NGS method covered region		
	BRCA1:NM_007294:c.4986+222A>G	rs3092987	66,67		Out of pre-NGS method covered region		
	BRCA1:NM_007294:c.4837A>G	rs1799966	46,25	Known			
	BRCA1:NM_007294:c.4485-63C>G	rs8176212	57,06		Out of pre-NGS method covered region		
	BRCA1:NM_007294:c.4485-137T>A	rs2236762	57,35		Out of pre-NGS method covered region		
	BRCA1:NM_007294:c.4358-2590T>G	rs8176194	48,96		Out of pre-NGS method covered region		
	BRCA1:NM_007294:c.4358-2885G>A	rs8176193	46,13		Out of pre-NGS method covered region		
	BRCA1:NM_007294:c.4308T>C	rs1060915	45,86	Known			
	BRCA1:NM_007294:c.4097-141A>C	rs799916	44,07		Out of pre-NGS method covered region		
	BRCA1:NM_007294:c.3548A>G	rs16942	51,05	Known			
	BRCA1:NM_007294:c.3113A>G	rs16941	46,46	Known			
	BRCA1:NM_007294:c.2612C>T	rs799917	45,34	Known			
	BRCA1:NM_007294:c.2311T>C	rs16940	46,85	Known			
	BRCA1:NM_007294:c.2082C>T	rs1799949	47,46	Known			
	BRCA1:NM_007294:c.2077G>A	rs4986850	48,35	Known			
	BRCA1:NM_007294:c.671-178G>A	rs8176151	60,71		Out of pre-NGS method covered region		
	BRCA1:NM_007294:c.593+167T>C	rs8176145	52,88		Out of pre-NGS method covered region		
	BRCA1:NM_007294:c.547+146A>T	rs8176140	41,03		Out of pre-NGS method covered region		
	BRCA1:NM_007294:c.213-161A>G	rs799912	50		Out of pre-NGS method covered region		
	BRCA1:NM_007294:c.1-134T>C	rs3765640	55,88	Known			
	BRCA1:NM_007294:c.4485-203_4485-199delAACCC	rs34250703	46.34		Out of pre-NGS method covered region		
	BRCA1:NM_007294:c.548-58_548-58delT	rs8176144	41.23		Out of pre-NGS method covered region		
	BRCA1:NM_007294:c.441+52_441+63delCTTTTTTTTTTT	.	54.23		Out of pre-NGS method covered region		
	BRCA1:NM_007294:c.441+36_441+38delCTT	rs147856441	49.57	Known			
V31							
	APC:NM_001127510:c.1-176G>A	.	65		Out of pre-NGS method covered region		
	APC:NM_001127510:c.136-53T>C	rs2304793	50,67	Known			
	APC:NM_001127510:c.423-256G>A	rs392179	100		Out of pre-NGS method covered region		
	APC:NM_001127510:c.645+129A>C	rs2289485	42,86	Known			
	APC:NM_001127510:c.645+173A>G	rs2289484	57,14	Known			
	APC:NM_001127510:c.934-132G>T	rs12521276	100		Out of pre-NGS method covered region		
	APC:NM_001127510:c.934-92G>C	rs80307314	64,56	Known			
	APC:NM_001127510:c.1458T>C	rs2229992	47,67	Known			
	APC:NM_001127510:c.1635G>A	rs351771	50,22	Known			
	**APC:NM_001127510:c.1660C>T**	**rs137854573**	**48,4**	**PAT**			
	APC:NM_001127510:c.1743+193G>A	rs351772	47,37	Known			
	**APC:NM_001127510:c.3264G>A**	**rs114774495**	**50,59**	**VUS**			
	APC:NM_001127510:c.4479G>A	rs41115	47,86	Known			
	APC:NM_001127510:c.5034G>A	rs42427	49,41	Known			
	APC:NM_001127510:c.5268T>G	rs866006	50,4	Known			
	APC:NM_001127510:c.5465T>A	rs459552	100		Could not be detected by CSCE		
	APC:NM_001127510:c.5880G>A	rs465899	47,14	Known			
	APC:NM_001127510:c.8532+86C>A	rs1804197	41,3	Known			
	APC:NM_001127511:c.1-134_1-133insG	.	46.67		Out of pre-NGS method covered region		
	APC:NM_001127510:c.934-133_934-132delTG	rs369189394	29.17		Out of pre-NGS method covered region		
	APC:NM_001127510:c.934-132_934-132delG	rs141013063	64.71		Out of pre-NGS method covered region		
	APC:NM_001127510:c.934-120_934-119insG	rs138003874	25		Out of pre-NGS method covered region		
	APC:NM_001127510:c.1959-145_1959-144insAGAA	rs3839284	47.73		Out of pre-NGS method covered region		

V32							
	MLH1:NM_000249:c.1-93G>A	rs1800734	60,64		Out of pre-NGS method covered region		
	MLH1:NM_000249:c.453+79A>G	rs4234259	43,08		Out of pre-NGS method covered region		
	MLH1:NM_000249:c.655A>G	rs1799977	66,67	Known			
	MLH1:NM_000249:c.678-211T>A	.	75		Out of pre-NGS method covered region		
	MLH1:NM_000249:c.1038+86T>C	rs2286939	50		Out of pre-NGS method covered region		
	MLH1:NM_000249:c.1039-78A>G	rs11129748	47,06		Out of pre-NGS method covered region		
	MLH1:NM_000249:c.1039-29A>T	rs6771325	46,15		Out of pre-NGS method covered region		
	MLH1:NM_000249:c.1410-169C>T	rs2286940	70		Out of pre-NGS method covered region		
	MLH1:NM_000249:c.1668-19A>G	rs9876116	41,51		Out of pre-NGS method covered region		
	MLH1:NM_000249:c.1990-121C>T	rs2241031	53,09		Out of pre-NGS method covered region		
	**MLH1:NM_000249:c.541_541delG**	**.**	**46.22**	**PAT**			
	MLH1:NM_000249:c.1897-167_1897-167delT	rs11290150	88.24		Out of pre-NGS method covered region		
V33							
	NF1:NM_000267:c.889-192G>T	.	100		Could not be detected by RNA-based analysis		
	NF1:NM_000267:c.2034G>A	rs2285892	100	Known			
	NF1:NM_000267:c.3197+139T>C	rs2071009	98,33		Could not be detected by RNA-based analysis		
	NF1:NM_000267:c.3315-130G>C	rs2072131	99,45		Could not be detected by RNA-based analysis		
	NF1:NM_000267:c.3496+33C>A	rs2066736	100		Could not be detected by RNA-based analysis		
	NF1:NM_000267:c.3974+46T>G	rs72813695	83,97		Could not be detected by RNA-based analysis		
	NF1:NM_000267:c.7126+37C>G	rs7405740	99,48		Could not be detected by RNA-based analysis		
	NF1:NM_000267:c.8097+71A>T	.	23,53		Could not be detected by RNA-based analysis		
	NF1:NM_000267:c.2002-118_2002-117insACACACACAC	.	28.57		Could not be detected by RNA-based analysis		
	**NF1:NM_000267:c.2956_2957insT**	**.**	**48.22**	**PAT**			
	NF1:NM_000267:c.3974+33_3974+34insTG	.	72.76		Could not be detected by RNA-based analysis		
	NF1:NM_000267:c.6085-29_6085-28insA	rs33925668	78.53		Could not be detected by RNA-based analysis		
V34							
	NF1:NM_000267:c.61-300C>T	rs2269856	66,67		Could not be detected by RNA-based analysis		
	NF1:NM_000267:c.61-123G>A	rs2269855	50,61		Could not be detected by RNA-based analysis		
	NF1:NM_000267:c.288+41G>A	rs2952976	47,83		Could not be detected by RNA-based analysis		
	NF1:NM_000267:c.480-90C>T	rs2905807	46,91		Could not be detected by RNA-based analysis		
	NF1:NM_000267:c.702G>A	rs1801052	49,55	Known			
	**NF1:NM_000267:c.731-1G>C**	**.**	**52,86**	**PAT**			
	NF1:NM_000267:c.888+108C>T	rs2953000	53,49		Could not be detected by RNA-based analysis		
	NF1:NM_000267:c.888+118G>T	rs2952999	54,67		Could not be detected by RNA-based analysis		
	NF1:NM_000267:c.1393-130A>T	rs2905875	48,99		Could not be detected by RNA-based analysis		
	NF1:NM_000267:c.1393-32T>C	rs2905876	43,77		Could not be detected by RNA-based analysis		
	NF1:NM_000267:c.1641+39T>C	rs2905880	54,49		Could not be detected by RNA-based analysis		
	NF1:NM_000267:c.2034G>A	rs2285892	46,09	Known			
	NF1:NM_000267:c.3197+139T>C	rs2071009	45,65		Could not be detected by RNA-based analysis		
	NF1:NM_000267:c.3315-130G>C	rs2072131	44,98		Could not be detected by RNA-based analysis		
	NF1:NM_000267:c.3496+33C>A	rs2066736	49,69		Could not be detected by RNA-based analysis		
	NF1:NM_000267:c.3974+46T>G	rs72813695	40,44		Could not be detected by RNA-based analysis		
	NF1:NM_000267:c.5205+23T>C	rs9894648	47,1		Could not be detected by RNA-based analysis		
	NF1:NM_000267:c.5546+19T>A	rs2285894	47,2		Could not be detected by RNA-based analysis		
	NF1:NM_000267:c.5546+117G>A	rs3815154	51,36		Could not be detected by RNA-based analysis		
	NF1:NM_000267:c.6085-29T>A	rs7406038	49,11		Could not be detected by RNA-based analysis		
	NF1:NM_000267:c.6085-28T>A	rs7406039	58,56		Could not be detected by RNA-based analysis		
	NF1:NM_000267:c.7126+37C>G	rs7405740	100		Could not be detected by RNA-based analysis		
	NF1:NM_000267:c.7395-29G>A	rs964288	44,59		Could not be detected by RNA-based analysis		
	NF1:NM_000267:c.7907+166C>T	rs7350943	53,33		Could not be detected by RNA-based analysis		
	NF1:NM_000267:c.8097+30G>T	.	20,69		Could not be detected by RNA-based analysis		
	NF1:NM_000267:c.889-191_889-190insT	rs35966725	35.29		Could not be detected by RNA-based analysis		
	NF1:NM_000267:c.1528-37_1528-36insT	rs67472948	44.86		Could not be detected by RNA-based analysis		
	NF1:NM_000267:c.2002-118_2002-117insACACACACACAC	.	20.45		Could not be detected by RNA-based analysis		
	NF1:NM_000267:c.3974+33_3974+34insTG	.	34.7		Could not be detected by RNA-based analysis		
V35							
	NF1:NM_000267:c.61-300C>T	rs2269856	100		Could not be detected by RNA-based analysis		

	NF1:NM_000267:c.61-123G>A	rs2269855	100		Could not be detected by RNA-based analysis		
	NF1:NM_000267:c.288+41G>A	rs2952976	100		Could not be detected by RNA-based analysis		
	NF1:NM_000267:c.480-90C>T	rs2905807	100		Could not be detected by RNA-based analysis		
	NF1:NM_000267:c.702G>A	rs1801052	97,9	Known			
	NF1:NM_000267:c.730+85A>T	.	20,48		Could not be detected by RNA-based analysis		
	NF1:NM_000267:c.888+108C>T	rs2953000	100		Could not be detected by RNA-based analysis		
	NF1:NM_000267:c.888+118G>T	rs2952999	100		Could not be detected by RNA-based analysis		
	NF1:NM_000267:c.1393-130A>T	rs2905875	100		Could not be detected by RNA-based analysis		
	NF1:NM_000267:c.1393-32T>C	rs2905876	100		Could not be detected by RNA-based analysis		
	NF1:NM_000267:c.1641+39T>C	rs2905880	100		Could not be detected by RNA-based analysis		
	NF1:NM_000267:c.1642-158A>G	rs17883230	81,03		Could not be detected by RNA-based analysis		
	NF1:NM_000267:c.5205+23T>C	rs9894648	100		Could not be detected by RNA-based analysis		
	NF1:NM_000267:c.5546+19T>A	rs2285894	99,25		Could not be detected by RNA-based analysis		
	NF1:NM_000267:c.5546+117G>A	rs3815154	100		Could not be detected by RNA-based analysis		
	NF1:NM_000267:c.6085-29T>A	rs7406038	94,88		Could not be detected by RNA-based analysis		
	NF1:NM_000267:c.6085-28T>A	rs7406039	100		Could not be detected by RNA-based analysis		
	NF1:NM_000267:c.7126+37C>G	rs7405740	100		Could not be detected by RNA-based analysis		
	NF1:NM_000267:c.7395-29G>A	rs964288	100		Could not be detected by RNA-based analysis		
	NF1:NM_000267:c.7907+166C>T	rs7350943	100		Could not be detected by RNA-based analysis		
	NF1:NM_000267:c.8050+20G>A	rs55747230	22,4		Could not be detected by RNA-based analysis		
	NF1:NM_000267:c.730+15_730+16insT	.	33.89		Could not be detected by RNA-based analysis		
	NF1:NM_000267:c.889-191_889-190insT	rs35966725	87.5		Could not be detected by RNA-based analysis		
	NF1:NM_000267:c.1528-37_1528-36insT	rs67472948	92.45		Could not be detected by RNA-based analysis		
	NF1:NM_000267:c.2002-118_2002-117insAC	rs141114572	43.4		Could not be detected by RNA-based analysis		
	NF1:NM_000267:c.3314+154_3314+154delT	rs17881842	84.21		Could not be detected by RNA-based analysis		
	**NF1:NM_000267: whole gene deletion**	Detected by I2HCP copy number		**PAT**			
V36							
	NF1:NM_000267:c.61-300C>T	rs2269856	100		Could not be detected by RNA-based analysis		
	NF1:NM_000267:c.61-123G>A	rs2269855	99,38		Could not be detected by RNA-based analysis		
	NF1:NM_000267:c.288+41G>A	rs2952976	100		Could not be detected by RNA-based analysis		
	NF1:NM_000267:c.480-90C>T	rs2905807	98,53		Could not be detected by RNA-based analysis		
	NF1:NM_000267:c.655-37A>C	rs375655707	46,49		Could not be detected by RNA-based analysis		
	NF1:NM_000267:c.702G>A	rs1801052	97,55	Known			
	NF1:NM_000267:c.888+108C>T	rs2953000	100		Could not be detected by RNA-based analysis		
	NF1:NM_000267:c.888+118G>T	rs2952999	100		Could not be detected by RNA-based analysis		
	**NF1:NM_000267:c.1381C>T**	**.**	**49,23**	**PAT**			
	NF1:NM_000267:c.1393-130A>T	rs2905875	100		Could not be detected by RNA-based analysis		
	NF1:NM_000267:c.1393-32T>C	rs2905876	99,6		Could not be detected by RNA-based analysis		
	NF1:NM_000267:c.1641+39T>C	rs2905880	99,55		Could not be detected by RNA-based analysis		
	NF1:NM_000267:c.5205+23T>C	rs9894648	49,25		Could not be detected by RNA-based analysis		
	NF1:NM_000267:c.5546+19T>A	rs2285894	46,97		Could not be detected by RNA-based analysis		
	NF1:NM_000267:c.5546+117G>A	rs3815154	52,46		Could not be detected by RNA-based analysis		
	NF1:NM_000267:c.6085-29T>A	rs7406038	46,63		Could not be detected by RNA-based analysis		
	NF1:NM_000267:c.6085-28T>A	rs7406039	54,27		Could not be detected by RNA-based analysis		
	NF1:NM_000267:c.7126+37C>G	rs7405740	54,17		Could not be detected by RNA-based analysis		
	NF1:NM_000267:c.7395-29G>A	rs964288	37,62		Could not be detected by RNA-based analysis		
	NF1:NM_000267:c.7970+166C>T;NF1:NM_000267:c.7907+166C>T	rs7350943	59,09		Could not be detected by RNA-based analysis		
	NF1:NM_000267:c.730+15_730+16insT	.	33.75		Could not be detected by RNA-based analysis		
	NF1:NM_000267:c.889-191_889-190insT	rs35966725	100		Could not be detected by RNA-based analysis		
	NF1:NM_000267:c.1528-37_1528-36insT	rs67472948	93.47		Could not be detected by RNA-based analysis		
	NF1:NM_000267:c.2002-118_2002-117insAC	rs141114572	46.94		Could not be detected by RNA-based analysis		
	NF1:NM_000267:c.3198-29_3198-29delT	.	20.49		Could not be detected by RNA-based analysis		
V37							
	NF1:NM_00267:c.61-123G>A	rs2269855	52,49		Could not be detected by RNA-based analysis		
	**NF1:NM_00267:c.278G>A**	**rs199474728**	**59,06**	**PAT**			
	NF1:NM_00267:c.288+41G>A	rs2952976	63,44		Could not be detected by RNA-based analysis		
	NF1:NM_00267:c.480-90C>T	rs2905807	45,05		Could not be detected by RNA-based analysis		
	NF1:NM_00267:c.587-140A>G	.	55,17		Could not be detected by RNA-based analysis		

	NF1:NM_00267:c.702G>A	rs1801052	58,9	Known			
	NF1:NM_00267:c.1393-130A>T	rs2905875	47,92		Could not be detected by RNA-based analysis		
	NF1:NM_00267:c.1393-32T>C	rs2905876	50,36		Could not be detected by RNA-based analysis		
	NF1:NM_00267:c.1641+39T>C	rs2905880	34,88		Could not be detected by RNA-based analysis		
	NF1:NM_00267:c.2034G>A	rs2285892	41,1	Known			
	NF1:NM_00267:c.3315-130G>C	rs2072131	48,21		Could not be detected by RNA-based analysis		
	NF1:NM_00267:c.3496+33C>A	rs2066736	53,33		Could not be detected by RNA-based analysis		
	NF1:NM_00267:c.3974+46T>G	rs72813695	38,46		Could not be detected by RNA-based analysis		
	NF1:NM_00267:c.5268+23T>C;NF1:NM_000267:c.5205+23T>C	rs9894648	36,73		Could not be detected by RNA-based analysis		
	NF1:NM_00267:c.5609+19T>A;NF1:NM_000267:c.5546+19T>A	rs2285894	44,9		Could not be detected by RNA-based analysis		
	NF1:NM_00267:c.5609+117G>A;NF1:NM_000267:c.5546+117G>A	rs3815154	43,22		Could not be detected by RNA-based analysis		
	NF1:NM_00267:c.6148-29T>A;NF1:NM_000267:c.6085-29T>A	rs7406038	35,86		Could not be detected by RNA-based analysis		
	NF1:NM_00267:c.7189+37C>G;NF1:NM_000267:c.7126+37C>G	rs7405740	100		Could not be detected by RNA-based analysis		
	NF1:NM_00267:c.7458-29G>A;NF1:NM_000267:c.7395-29G>A	rs964288	54,29		Could not be detected by RNA-based analysis		
	NF1:NM_00267:c.8160+30G>T;NF1:NM_000267:c.8097+30G>T	.	33,33		Could not be detected by RNA-based analysis		
	NF1:NM_00267:c.730+15_730+16insT	.	25.41		Could not be detected by RNA-based analysis		
	NF1:NM_00267:c.1528-37_1528-36insT	rs67472948	47.26		Could not be detected by RNA-based analysis		
	NF1:NM_000267:c.2002-118_2002-117insACACACACACAC	.	35		Could not be detected by RNA-based analysis		
	NF1:NM_000267:c.3974+33_3974+34insTG	.	32.61		Could not be detected by RNA-based analysis		
	NF1:NM_000267:c.6085-29_6085-28insA	rs33925668	54.05		Could not be detected by RNA-based analysis		
V38							
	NF2:NM_000268:c.241-85G>T	rs5763378	51,43		Could not be detected by RNA-based analysis		
	NF2:NM_000268:c.364-39A>C	rs2530664	46,58		Could not be detected by RNA-based analysis		
	NF2:NM_000268:c.600-154G>A	rs2071622	59,04		Could not be detected by RNA-based analysis		
	NF2:NM_000268:c.886-205C>T	rs2252472	72,73		Could not be detected by RNA-based analysis		
	NF2:NM_000268:c.1122+129A>T	rs2527336	52,73		Could not be detected by RNA-based analysis		
	NF2:NM_000268:c.1123-51C>T	rs13055076	43,08		Could not be detected by RNA-based analysis		
	NF2:NM_000268:c.1575-67G>A	rs140086	99,18		Could not be detected by RNA-based analysis		
	NF2:NM_000268:c.1737+1623T>C	rs7291645	100		Could not be detected by RNA-based analysis		
	**NF2:NM_000268: exon 15 deletion**	Detected by I2HCP copy number		**PAT**			
V39							
	SMARCB1:NM_003073:c.1-117C>T	rs11090285	69,23		Could not be detected by RNA-based analysis		
	SMARCB1:NM_003073:c.363-154G>C	rs9608185	100		Could not be detected by RNA-based analysis		
	SMARCB1:NM_003073:c.363-153C>T	rs9608186	98,33		Could not be detected by RNA-based analysis		
	SMARCB1:NM_003073:c.500+234C>G	rs738797	100		Could not be detected by RNA-based analysis		
	SMARCB1:NM_003073:c.501-164C>T	rs11090286	62,96		Could not be detected by RNA-based analysis		
	SMARCB1:NM_003073:c.501-87A>G	rs5760030	100		Could not be detected by RNA-based analysis		
	SMARCB1:NM_003073:c.628+66G>C	rs5751738	99,45		Could not be detected by RNA-based analysis		
	SMARCB1:NM_003073:c.628+118C>T	rs5760032	50,79		Could not be detected by RNA-based analysis		
	SMARCB1:NM_003073:c.628+200T>C	rs17003912	54,55		Could not be detected by RNA-based analysis		
	SMARCB1:NM_003073:c.629-248A>G	rs2073392	100		Could not be detected by RNA-based analysis		
	SMARCB1:NM_003073:c.629-62A>G	rs9608192	48,29		Could not be detected by RNA-based analysis		
	SMARCB1:NM_003073:c.795+121G>A	rs9612452	44,14		Could not be detected by RNA-based analysis		
	SMARCB1:NM_003073:c.795+153A>G	rs2073393	43,4		Could not be detected by RNA-based analysis		
	SMARCB1:NM_003073:c.795+184A>T	rs2070458	65		Could not be detected by RNA-based analysis		
	SMARCB1:NM_003073:c.795+198T>C	rs2070459	38,89		Could not be detected by RNA-based analysis		
	SMARCB1:NM_003073:c.897G>A	rs2229354	49,89	Known			
	SMARCB1:NM_003073:c.987-176A>C	rs2267045	62,16		Could not be detected by RNA-based analysis		
	SMARCB1:NM_003073:c.987-139G>A	rs2267046	53,57		Could not be detected by RNA-based analysis		
	SMARCB1:NM_003073:c.1118+163G>T	rs17003998	53,06		Could not be detected by RNA-based analysis		
	SMARCB1:NM_003073:c.1119-164G>A	rs9612483	60		Could not be detected by RNA-based analysis		
	SMARCB1:NM_003073:c.1119-41G>A	rs5030613	55		Could not be detected by RNA-based analysis		
	SMARCB1:NM_003073:c.629-130_629-130delC	rs34737764	51.32		Could not be detected by RNA-based analysis		
	SMARCB1:NM_003073:c.986+54_986+55insAA	rs5844569	44.27		Could not be detected by RNA-based analysis		
	SMARCB1:NM_003073:c.986+143_986+145delTCC	rs34624937	75		Could not be detected by RNA-based analysis		
	SMARCB1:NM_003073:c.1158+112_1158+113insG	rs34399789	41.67		Could not be detected by RNA-based analysis		
V40							
	NF2:NM_000268:c.1-110G>C	rs1800540	100		Could not be detected by RNA-based analysis		

	NF2:NM_000268:c.240+171T>A	rs2530662	42,11		Could not be detected by RNA-based analysis		
	NF2:NM_000268:c.241-85G>T	rs5763378	50,28		Could not be detected by RNA-based analysis		
	**NF2:NM_000268:c.241-13T>A**	**.**	**50**	**PAT**			
	NF2:NM_000268:c.600-154G>A	rs2071622	49,44		Could not be detected by RNA-based analysis		
	NF2:NM_000268:c.886-205C>T	rs2252472	62,5		Could not be detected by RNA-based analysis		
	NF2:NM_000268:c.1123-51C>T	rs13055076	50,76		Could not be detected by RNA-based analysis		
	NF2:NM_000268:c.1575-67G>A	rs140086	99,6		Could not be detected by RNA-based analysis		
	NF2:NM_000268:c.1737+1623T>C	rs7291645	29,27		Could not be detected by RNA-based analysis		
	NF2:NM_000268:c.599+41_599+41delT	.	21.8		Could not be detected by RNA-based analysis		
	NF2:NM_000268:c.1447-227_1447-226insTGAGGGA	rs3842713	27.03		Could not be detected by RNA-based analysis		

Table S5: Results from Validation set (n=40)

Sample	HC syndrome	Genes	Pathogenic Variant	Detected
V1	FAP	*MUTYH*	NM_012222.2:c.1187G>A; c.481G>C	YES
V2	FAP	*APC*	NM_001127510.2:c.1548+1G>C	YES
V3	HNPPC	*MSH2*	NM_000251.2:c.518T>G	YES
V4	HBOC	*BRCA2*	NM_000059.3:c.5350_5351delAAinsT	YES
V5	HNPPC	*MLH1*	NM_000249.3:c.2150_2153dup	YES
V6	HNPPC	*PMS2*	NM_000535:.5:c.943C>T	YES
V7	FAP	*APC*	NM_001127510.2:c.1312+3A>G	YES
V8	LI-FRAUMENI	*TP53*	NM_001126112.2:c.743G>A	YES
V9	HNPPC	*MSH6*	NM_000179.2:c.1618_1620delCTT	YES
V10	HBOC	*BRCA1*	NM_007294.2:c.3770_3771delAG	YES
V11	HBOC	*BRCA1*	Unknown disease-causing mutation	---
V12	HBOC	*BRCA2*	NM_000059.3:c.1308_1309delGA	YES
V13	HNPPC	*PMS2*	NM_000535.5:c.59G>A	YES
V14	HBOC	*BRCA2*	NM_000059.3:c.3936_3954del(19bp)	YES
V15	FAP	*APC*	NM_001127510.2:c.2344A>T	YES
V16	FAP	*MUTYH*	NM_012222.2:c.1187G>A	YES
V17	HBOC	*BRCA2*	NM_000059.3:c.262_263delCT	YES
V18	HBOC	*BRCA1*	NM_007294.2:c.3869_3870delAA	YES
V19	HNPPC	*MSH2*	NM_000251.2:c.689_691delCTGinsTT	YES
V20	HNPPC	*PMS2*	NM_000535.5:c.780delC	YES
V21	HNPPC	*MLH1*	Unknown disease-causing mutation	---
V22	HBOC	*BRCA1*	NM_007294.2:c.68_69delAG	YES
V23	HBOC	*BRCA2*	NM_000059.3:c1128delT	YES
V24	FAP	*APC*	NM_001127510.2:c.3183_3187delACAAA	YES
V25	HNPPC	*MSH2*	NM_000251.2:c.388_389delCA	YES
V26	HBOC	*BRCA2*	NM_000059.3:c.7230delT	YES

V27	HNPPC	*MSH6*	NM_000179.2:c.2906_2907del	YES
V28	HBOC	*BRCA2*	NM_000059.3:c.5722_5723delCT	YES
V29	HBOC	*BRCA1*	NM_007294.2:c.4107_4110dupATCT	YES
V30	HBOC	*BRCA1*	Unknown disease-causing mutation	---
V31	FAP	*APC*	NM_001127510.2:c.1660C>T; c.3264C>A	YES
V32	HNPPC	*MLH1*	NM_000249.3:c.542delG	YES
V33	NF1	*NF1*	NM_000267.3: c.2957_2958insT	YES
V34	NF1	*NF1*	NM_000267.3:c.731-1G>C	YES
V35	NF1	*NF1*	NM_000267.3: whole gene deletion	YES
V36	NF1	*NF1*	NM_000267.3: c.1381C>T	YES
V37	NF1	*NF1*	NM_000267.3: c.278G>A	YES
V38	NF2	*NF2*	NM_000268.3: exon 15 deletion	YES
V39	SCHWANNOMATOSIS	*SMARCB1*	Unknown disease-causing mutation	---
V40	NF2	*NF2*	NM_000268.3: c.241-13T>A	YES

Sample	HBOC	FAP	HNPCC	NF1	NF2
**T1**	3	0	2	2	2
**T2**	2	0	1	1	3
**T3**	2	0	1	1	3
**T4**	2	0	1	1	2
**T5**	2	0	1	1	2
**T6**	7	0	4	2	4
**T7**	2	0	1	1	1
**T8**	7	0	4	2	4
**T9**	1	0	0	1	1
**T10**	4	0	2	1	3
**T11**	10	1	6	4	4
**T12**	5	0	3	2	4
**T13**	1	0	0	1	1
**T14**	6	0	3	1	4
**T15**	3	0	2	1	2
**T16**	3	0	2	1	3
**T17**	18	5	8	6	9
**T18**	7	0	3	1	3
**T19**	3	0	2	1	3
**T20**	2	0	1	1	2
**T21**	10	0	4	2	4
**T22**	9	0	4	3	4
**T23**	5	0	3	1	3
**V1**	4	0	3	1	3
**V2**	8	1	5	3	4
**V3**	1	0	0	1	1
**V4**	4	0	2	1	3
**V5**	47	7	27	10	4
**V6**	2	0	1	1	2
**V7**	7	1	4	2	4
**V8**	17	5	11	2	5
**V9**	1	0	0	1	1
**V10**	2	0	1	1	1
**V11**	2	0	1	1	3
**V12**	2	0	1	1	3
**V13**	3	0	2	1	3
**V14**	2	0	1	1	2
**V15**	7	2	5	1	4
**V16**	7	1	4	1	4
**V17**	3	0	2	1	2
**V18**	3	0	2	1	2
**V19**	2	0	1	1	2

**V20**	3	0	2	1	3
**V21**	2	0	1	1	3
**V22**	2	0	1	2	3
**V23**	3	0	2	1	2
**V24**	3	0	2	1	2
**V25**	11	1	5	3	4
**V26**	6	1	4	1	4
**V27**	5	0	3	1	3
**V28**	2	0	1	1	3
**V29**	5	0	3	1	2
**V30**	4	0	3	2	3
**V31**	5	0	2	2	3
**V32**	15	1	7	5	7
**V33**	31	7	17	6	10
**V34**	2	0	1	1	2
**V35**	2	0	1	1	3
**V36**	2	0	1	1	2
**V37**	2	0	1	1	2
**V38**	1	0	0	1	2
**V39**	8	1	4	1	3
**V40**	14	2	8	5	5

**Gene**	**Samples**	**MLPA analysis (-)**	**I2HCP CNA analysis (-)**	**MLPA analysis (+)**	**I2HCP CNA analysis** [illegible]	
NF1	46	43	43	3	3	(whole gene dele[illegible]
CDKN2A	6	1	1	5	5	
BRCA1	16	16	15 (1 undetermined)	0	0	
BRCA2	16	16	15 (1 undetermined)	0	0	
TP53	16	16	15 (1 undetermined)	0	0	
MUTYH	1	1	1	0	0	
MSH2	25	25	25	0	0	
MSH6	12	12	12	0	0	
MLH1	25	25	25	0	0	
PMS2	1	1	1	0	0	
CDH1	4	3	3	1 (exons 1-2 deleted)	1	(exon 2 deleted, [illegible]
TSC1	1	1	1	0	0	
TSC2	1	1	1	0	0	
APC	1	1	1	0	0	
**TOTAL**	**171**	**162**	**159**	**9**	**9**	

SUPPLEMENTARY TABLES AND FIGURES

Table. S1: List of tested genes. The table includes the genes tested for the five conditions with pre-defined groups. Red dots indicate genes tested in the pre-NGS workflows and black dots indicate genes included in the new gene sets. The list below the table includes the remaining genes present in the I2HCP panel.

Table S2: Training set results. Training set contains 23 samples previously genetically characterized. For each sample, gene analyzed and pathogenic mutation detected in each approach is indicated. In addition, for non-identified information on variant context is provided. Pathogenic mutations are colored depending on mutation type: blue: substitution, light green: small deletion or insertion; dark green: medium-sized deletion or insertion; red: big deletions. HomoP indicates variants are within a homopolymer region. Low coverage indicates coverage <30x.

Table S3: Sequencing and coverage statistics. Sequencing and coverage statistics of samples in the training (T) and validation (V) sets. Coverage statistics were calculated over the ROIs. Cx indicates the percentage of ROI covered by more than x reads. Ux indicates the uniformity, that is, the percentage of reads within +/- x% of the mean coverage.

Table S4: Concordance with previously detected variants. Variants detected in the training (T) and validation (V) sets by I2HCP second approach (V2) in genes previously analyzed by pre-NGS tests. For each variant, dbSNP identifier (rs), frequency, classification (previously known (Known), pathogenic (PAT) or variant of unknown significance (VUS)) are indicated. A short explanation for variants detected by only one method (pre-NGS analysis or I2HCP) is included. In addition, variants also detected by I2HCP first approach (V1) are indicated.

Table S5: Validation set results. Validation set contains 40 samples previously genetically characterized. For each sample, gene analyzed and pathogenic mutation detected are indicated. Variants are colored depending on mutation type: blue: substitution, light green: small deletion or insertion; dark green: medium-sized deletion or insertion; red: big deletions. VUS are underlined.

Table S6: Complementary Sanger sequencing tests required. The table shows the number of ROIs to be Sanger sequenced due to low coverage for each sample of the training (T) and validation (V) sets if tested for one of the five pre-defined groups of genes.

Table S7: Comparison of CNA analyses. One hundred seventy-one copy number alteration (CNA) analyses in 100 patients comprising 14 different genes were performed by MLPA and I2HCP exon-level copy-number calling algorithm. One sample, comprising 3 MLPAs, was non-assessable by I2HCP due to quality criteria.

Figure S1: Bait redesign. Example of one exon of *TP53* where bait redesign and rebalancing were applied. Y-axis indicates the depth of coverage of a region of the *TP53* gene (X-axis, reference genome) for different samples. Grey lines: samples using the first approach with V1 baits. Colored lines: samples from the second approach after bait redesign (V2 baits). Yellow and blue lines represent bait density in V1 and V2 (respectively) over the X-axis. A green doted line denotes the recurrent low coverage region of the *TP53* (grey lines going under the 30x depth coverage, denoted by a pale yellow line) that was clearly improved after bait redesign.

Figure S2: Examples of coverage per sample. Global coverage as in Figure 3c for 12 representative samples of the

validation set.

<u>Figure S3</u>: Regions with recurrent low coverage. A) Plot showing coverage and %GC content as in Figure 3b. Exons with coverage below 30x in at least 1bp in ≥75% of samples are highlighted in red. Detailed coverage of the two highlighted exons with highest mean coverage are shown. B) List of all highlighted exons in A.

<u>Figure S4:</u> Analysis pipeline. Diagram of the I2HCP data analysis pipeline.

<u>Figure S5</u>: **New genetic testing workflow solved uncertain clinical diagnosis**. A patient with clinical suspicion of Neurofibromatosis Type 1 (3 neurofibromas, 1 glioblastoma multiforme, 1 malignant fibrous histiocytoma) (a) was analyzed with the I2HCP. A deletion in *CDKN2A* gene was detected by the exon-level copy-number calling algorithm. (b). Grey dots represent the normalized mean depth of coverage of 27 ROIs close to *CDKN2A* in chromosome 9; black dots represent normalized patient's sample coverage of the same ROIs and red dots indicate *CDKN2A* deleted exons. (c). MLPA analysis validated the loss of *CDKN2A*. Deletion of the whole *CDKN2B* gene was also detected, a locus that was not included in I2HCP. Red dots indicate the deleted region evidenced by MLPA analysis).

<u>Figure S6</u>: **Frequency of pathogenic variants.** The graph depicts the frequency of all pathogenic variants present in the training and validation sets. SNV: single nucleotide variant.
